# Thiophenes—Naturally Occurring Plant Metabolites: Biological Activities and In Silico Evaluation of Their Potential as Cathepsin D Inhibitors

**DOI:** 10.3390/plants11040539

**Published:** 2022-02-17

**Authors:** Sabrin R. M. Ibrahim, Abdelsattar M. Omar, Alaa A. Bagalagel, Reem M. Diri, Ahmad O. Noor, Diena M. Almasri, Shaimaa G. A. Mohamed, Gamal A. Mohamed

**Affiliations:** 1 Department of Chemistry, Preparatory Year Program, Batterjee Medical College, Jeddah 21442, Saudi Arabia; 2 Department of Pharmacognosy, Faculty of Pharmacy, Assiut University, Assiut 71526, Egypt; 3 Department of Pharmaceutical Chemistry, Faculty of Pharmacy, King Abdulaziz University, Jeddah 21589, Saudi Arabia; asmansour@kau.edu.sa; 4 Center for Artificial Intelligence in Precision Medicines, King Abdulaziz University, Jeddah 21589, Saudi Arabia; 5 Department of Pharmaceutical Chemistry, Faculty of Pharmacy, Al-Azhar University, Cairo 11884, Egypt; 6 Department of Pharmacy Practice, Faculty of Pharmacy, King Abdulaziz University, Jeddah 21589, Saudi Arabia; abagalagel@kau.edu.sa (A.A.B.); rdiri@kau.edu.sa (R.M.D.); aonoor@kau.edu.sa (A.O.N.); dalmasri@kau.edu.sa (D.M.A.); 7 Faculty of Dentistry, British University, Suez Desert Road, Cairo 11837, Egypt; shaimaag1973@gmail.com; 8 Department of Natural Products and Alternative Medicine, Faculty of Pharmacy, King Abdulaziz University, Jeddah 21589, Saudi Arabia; gahussein@kau.edu.sa

**Keywords:** thiophenes, Asteraceae, biosynthesis, bioactivities, in silico studies, cathepsin D, spectral data

## Abstract

Thiophenes represent a small family of natural metabolites characterized by one to five thiophene rings. Numerous plant species belonging to the family Asteraceae commonly produce thiophenes. These metabolites possess remarkable bioactivities, including antimicrobial, antiviral, anti-inflammatory, larvicidal, antioxidant, insecticidal, cytotoxic, and nematicidal properties. The current review provides an update, reported over the past seven years, on the natural thiophene derivatives, including their sources, biosynthesis, spectral data, and bioactivities since the last review published in 2015. Additionally, the potential drug targets of the compounds were predicted using the SuperPred webserver, an AI (artificial intelligence) tool. In silico studies were conducted for cathepsin D with thiophene derivatives, including ADMET (drug absorption/distribution/metabolism/excretion/and toxicity) properties prediction, molecular docking for the binding interaction, and molecular dynamics to evaluate the ligand–target interaction stability under simulated physiological conditions.

## 1. Introduction

Heterocyclic compounds play a remarkable role in the field of bioactive metabolites search. It is noteworthy that >75% of clinically utilized drugs possess heterocyclic moiety in their chemical skeleton [1]. Sulfur belongs to chalcogens that are the 16 group elements of the periodic table. Sulfur is a ubiquitous heteroatom in medicinal chemistry that can bond to various atoms, including nitrogen, oxygen, carbon, halides, and phosphorus. Several sulfur-based functionalities have become privileged pharmacophores in synthesizing new derivatives that contribute to drug discovery [2]. In living organisms, it exhibits a remarkably wide range of redox potentials and states, producing many sulfur species that take part in diverse biological processes. Thioethers and thiols can form sulfonium ions by donating electrons to other organic species, revealing their ability to stabilize a negative charge on a neighboring carbon [3]. They can undergo sequential oxidation to sulfoxides and sulfones, which have diverse biological roles. For example, S-adenosylmethionine (SAM, a sulfonium compound) mediates most biochemical methylation reactions in cell metabolism [4].

S-containing species have a strong electron-withdrawing nature, resistance to reduction at sulfur, stability against hydrolysis, and preference for two electrons over radical processes, which makes this group of compounds applicable to many drug research fields [5]. Their diverse pharmacological activities makes them a first choice for incorporation via the hybrid approach, which is used in most of the required medicines available on the market [5]. It was reported that 41 sulfur-containing commercial drugs appeared in the Top 200 Pharmaceuticals by Retail Sales in 2019 worldwide; 20.5% contain a sulfur atom [6].

Natural products have attracted significant attention as a potential source of S-containing compounds for drug discovery. The well-known conotoxin, ecteinascidin 743 (ET-743), and penicillin are examples of natural sulfur-containing clinical drugs. Furthermore, many sulfur-containing drugs are derived from natural products, e.g., phthalascidin and ixabepilone for cancer treatments, rosuvastatin for hyperlipidemia, and dalfopristin and quinupristin for infectious diseases [7].

**Figure 1:**
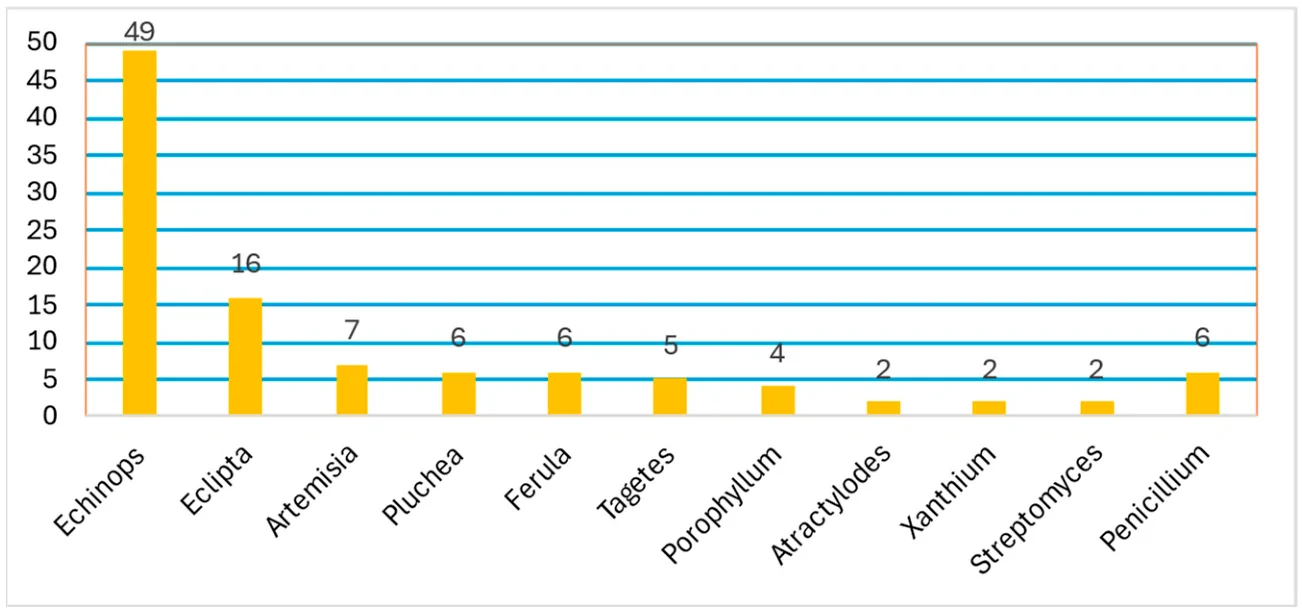
Number of reported thiophenes from various sources.

Thiophenes are among the heterocyclics that have been the focus of research interest over the last decades. They are a class of sulfur-containing molecules usually composed of one to five thiophene units connected at the α-position and often have various alkyl groups at the α’-carbon of the terminal ring [8]. Thiophene derivatives have beneficial applications in the dye, pharmaceutical, and agrochemical industries [9,10]. Interestingly, many of the approved drugs available in the markets have thiophene moiety, including antiasthma, NSAIDs (non-steroidal anti-inflammatory drugs), diuretics, anticancer, and antihistaminic drugs [11,12]. Naturally occurring thiophenes represent rare constituents reported from these metabolites, which have been isolated from various Asteraceae genera: *Echinops*, *Eclipta*, *Pluchea*, *Artemisia*, *Tagetes*, *Porophyllum*, *Atractylodes*, and *Xanthium* [8]. Additionally, some are reported from *Ferula* (family Apiaceae), as well as from actinomycetes (*Streptomyces*) and fungi (*Penicillium*) (Figure 1).

**Figure 2:**
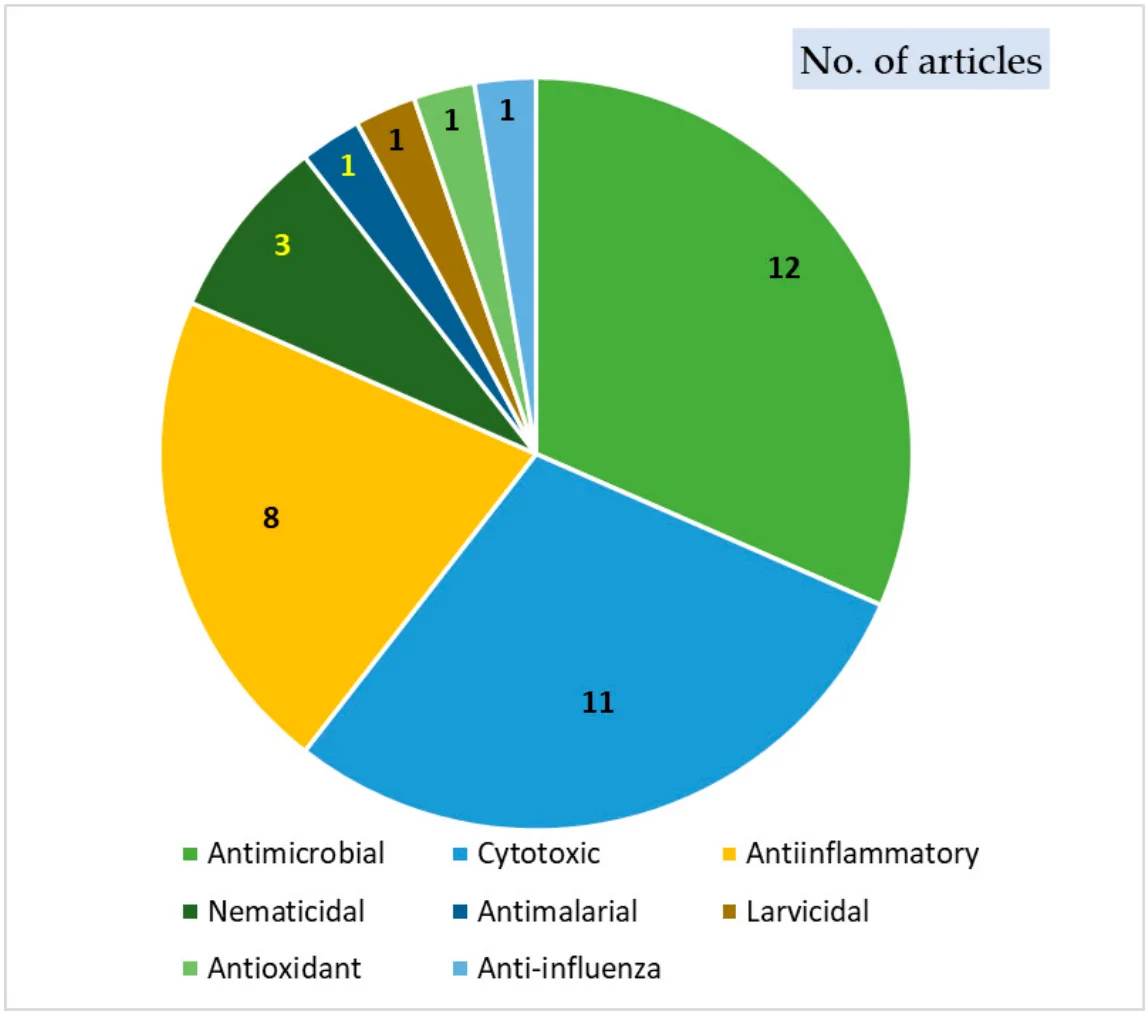
Biological activities of thiophenes.

They are produced as a chemical defense mechanism and are toxic to various pathogens, such as insects, nematodes, bacteria, and fungi [13,14]. Biosynthetically, they are derived from fatty acids or polyacetylenes through acetylene intermediates; therefore, they are classified as acetylenic thiophenes. Indeed, many of the reported derivatives possess an alkyl chain with an acetylenic unit that may contain chiral centers due to introducing a hydroxy group [8]. These metabolites have remarkable biological and pharmacological effectiveness, including antiviral, antimicrobial, antileishmanial, anti-inflammatory, larvicidal, antioxidant, insecticidal, HIV-1 (human immunodeficiency virus-1) protease-inhibitory, cytotoxic, nematicidal, and phototoxic activities [8,15,16,17,18,19,20] (Figure 2).

In our previous review, 96 natural thiophene derivatives from various plant species belonging to the Asteraceae family were listed up to 2015, with a particular focus on their biosynthesis, bioactivities, and physical and spectral data [8]. Recently, several reviews on synthetic thiophene-based derivatives, including their anti-inflammatory and anticancer activity, spectroscopic properties, and synthesis, have been published [21,22,23,24]. On the other hand, there is no available review on naturally occurring thiophene derivatives from plant sources.

**Scheme 1:**
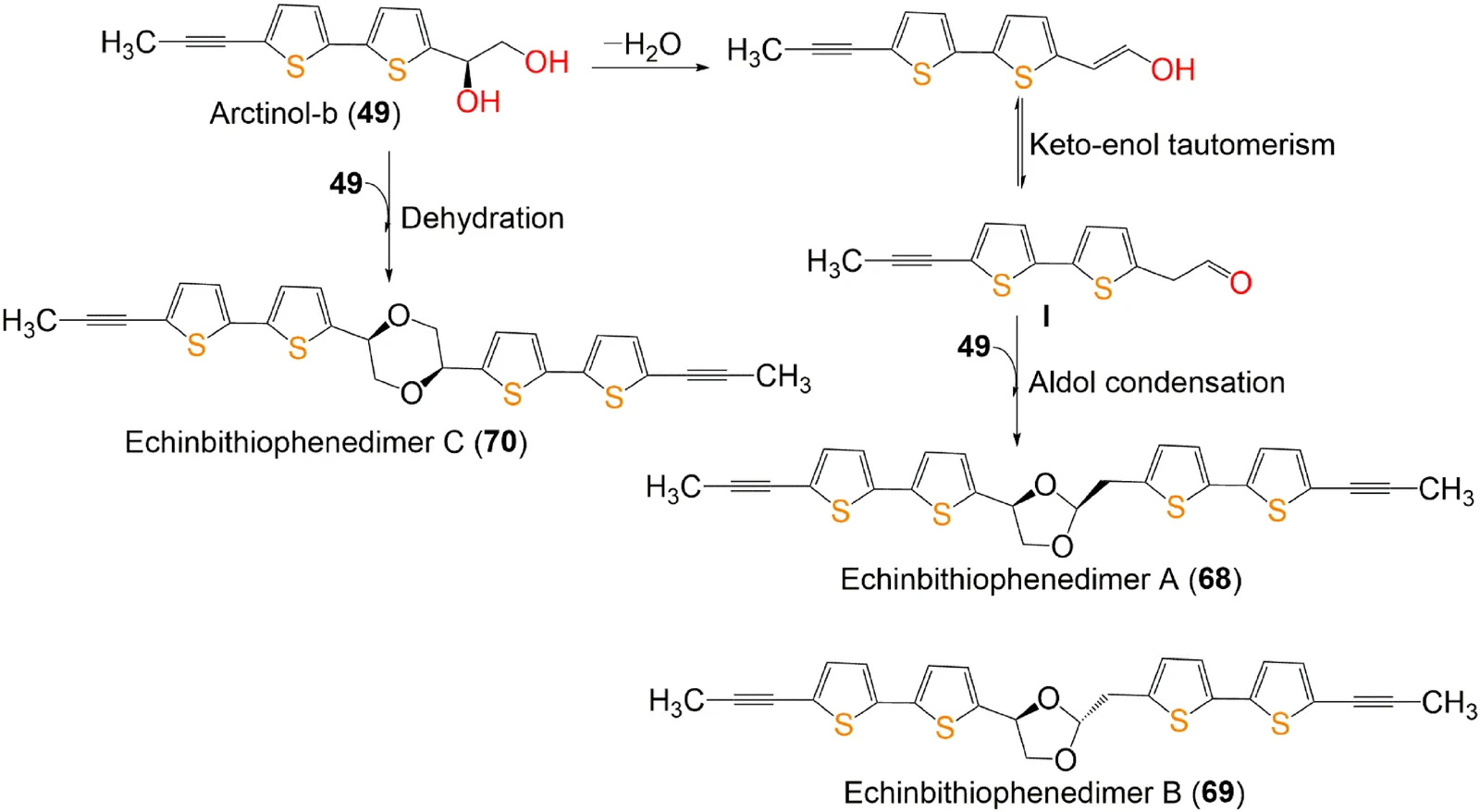
Proposed biosynthetic pathway of dimeric bithiophenes **68**–**70** from arctinol-b (**49**) [17].

**Scheme 2:**
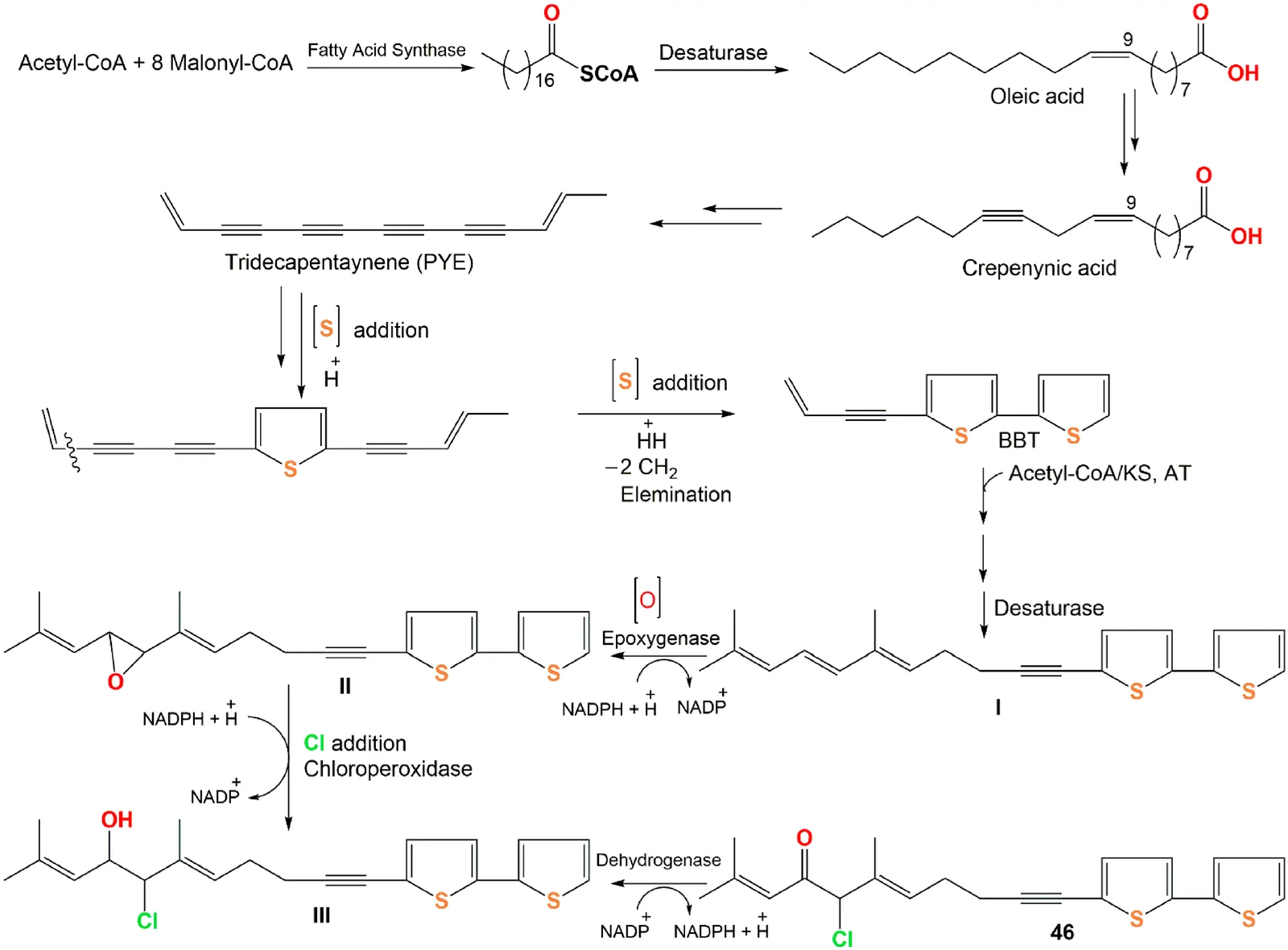
Proposed biosynthetic pathway of **46** [44,52,53,54].

Therefore, the current review aims to provide an update on the naturally reported thiophene derivatives over the past seven years, including their sources, biosynthesis, spectral data, and bioactivities. In total, 96 compounds have been listed and categorized according to the number of rings into mono-, bi-, ter, and quinque-thiophenes and miscellaneous derivatives. Additionally, their source, molecular weights and formulae, location, and fraction/extract from which they were isolated are listed in Table 1. The physical constants and spectral data of the newly reported thiophenes (Table S1) from 2015 to 2021 have been included. Further, their possible biosynthetic pathways are illustrated in Scheme 1 and Scheme 2, and their bioactivities are highlighted in Table 2. We hope that this review can help natural product researchers in the structural characterization of these metabolites and guide medicinal chemists in the synthesis of potentially more active new thiophene derivatives. A systematic search of the published data was performed in various databases, including Web of Science, PubMed, Scopus, and Google scholar. Moreover, published papers by different publishers, such as ACS (American Chemical Society), Elsevier, Bentham, Sage, Wiley, Taylor & Francis, Thieme Medical, and Springer, were surveyed. No language restrictions were applied.

Natural proteins (NPs) are biologically active molecules with a myriad of structural and functional diversity. They enable the innovative design of synthetic compounds used in medicines, along with many more crucial aspects of molecular medicine, including, but not limited to anti-cancer and anti-viral drugs currently in use. Many of them have proven to be incredibly useful in treating a plethora of diseases. Despite its many attributes, the speed and yields of NP-based drug discovery have significantly dropped since the golden period of 1950–1960. AI can aid structure-based drug discovery by predicting the protein targets of the potential NPs, thus assisting in the prediction of compound–target interactions and safety profiles. SuperPred, a prediction webserver for anatomical therapeutic chemical (ATC) codes and compound target prediction, was used to predict the potential target for these thiophene derivatives. Based on the outcomes of the SuperPred prediction, we selected cathepsin D, one of the amplest lysosomal proteases, which is also implicated in the pathogenesis of several diseases: cancer, osteoarthritis, and possibly Alzheimer’s disease. Furthermore, in silico analyses, including ADMET properties prediction, molecular docking for the protein ligands binding interaction, and molecular dynamics to evaluate the ligand–target interaction stability under simulated physiological conditions, were also implemented.

## 2. Structural Characterization of Thiophenes

The structures of the reported thiophenes were elucidated by various spectral tools, such as 1D (one-dimensional) (^1^H and ^13^C) and 2D NMR (two-dimensional nuclear magnetic resonance spectroscopy) techniques, COSY (homonuclear correlation spectroscopy), HSQC (heteronuclear single quantum coherence), HMBC (heteronuclear multiple bond correlation), and NOESY (nuclear Overhauser effect spectroscopy), combined with other methods (UV (ultraviolet), IR (infrared), MS (mass spectrometry), elemental analysis). The reported spectral and physical data of the newly reported thiophenes are listed in Table S1. The relative configuration was determined by NOESY and ROESY (rotating frame Overhauser effect spectroscopy), as well as by [α]_D_ measurement [34]. The exciton-coupled circular dichroism (ECCD) analysis and electronic circular dichroism (ECD) calculations were utilized to assess the absolute configuration by comparing the theoretical and experimental CD spectra [16,17,37,49]. Additionally, the determination of the absolute configuration was carried out using Mosher’s method and analyzing chemical shift differences between (*S*)- and (*R*)-MTPA [16]. The X-ray structure crystallographic analysis of the crystalline derivatives was another tool utilized for the absolute configuration determination [49]. It was found that some compounds had no names; therefore, they are named here using the IUPAC system for nomenclature. Further, some compounds had the same molecular formulae and structures with different nomenclatures. On the other hand, some metabolites had more than one name.

## 3. Biosynthesis of Thiophenes

The detailed biosynthesis of thiophenes was discussed previously [8]. In this work, the recently reported biosynthetic pathways is discussed.

Wu et al. reported the biogenetic pathways of dimeric bithiophenes **68**–**70** (Scheme 1). These compounds had an unparalleled dimeric bithiophene skeleton containing two bithiophene units linked by uncommon cyclic diether units. It was proposed that they may have originated from arctinol-b (**49**). For **68** and **69**, the formation of the 1,3-dioxolane ring may be obtained from an aldol condensation. Firstly, a key intermediate (**I**) is produced from **49** by dehydration and keto–enol tautomerism. After that, an aldol condensation among **49** and **I** would give **68** and **69**. Additionally, an intermolecular dehydration reaction between two **49** molecules forms the 1,4-dioxane unit to give **70** [17].

Compound **46** originates from oleic acid. The latter is changed into PYE (trideca-3,5,7,9,11-pentayn-l-ene) through successive desaturation steps and the shortening of the chain via crepenynic acid [52]. After that, PYE is changed into 5-BBT (5-(but-3-en-1-ynyl)-2,2′-bithiophene) via introducing a sulfur atom and ring formation that is most probably a two-step reaction [44]. Repeated elongation and desaturation of BBT yield **I**. Then, the double bond epoxidation produces oxirane (epoxy) intermediate **II**, subsequent addition of chloride by chloroperoxidase forms **III**, which performs additional dehydrogenation to yield **46** [53,54] (Scheme 2).

## 4. Biological Activities of Thiophenes

The reported thiophenes were investigated for various bioactivities. In this regard, these metabolites are associated with some types of biological actions, including antimicrobial, antiviral, anti-inflammatory, larvicidal, antioxidant, insecticidal, cytotoxic, and nematicidal effects. The results of the most active metabolites are summarized.

### 4.1. Anti-Inflammatory Activity

Inflammation is a host body defense mechanism that enables the body to survive during injury or infection and maintains the homeostasis of tissues in noxious conditions [55].

Endogenous NO (nitric oxide) plays a critical role in maintaining the homeostasis of varied cellular functions. NO local concentrations are highly dynamic, as independent enzymatic pathways regulate the synthesis. NO has been shown to modulate inflammation, decreasing the secretion of pro-inflammatory cytokines in human alveolar macrophages challenged with bacterial lipopolysaccharides (LPS) while not altering the basal cytokine levels. Drugs used for managing inflammatory disorders relieve these ailments, but they may have life-threatening consequences [56]. Therefore, there is great enthusiasm in developing new and safe remedies for treating inflammation from natural sources. The reported studies revealed that the anti-inflammatory activity of thiophenes could be due to the inhibition of of the NF-κB (nuclear factor-κB) pathway, which regulates the expression of pro-inflammatory cytokines and chemokines [57].

The reported studies revealed that thiophenes prohibited TNF-α (tumor necrosis factor-α), IL-6 (interleukin-6), and 5-LOX (5-lipoxygenase), as well as NO production. Thus, their inflammatory activity could be due to the inhibition of NF-κB and NO synthase [58].

**Figure 3:**
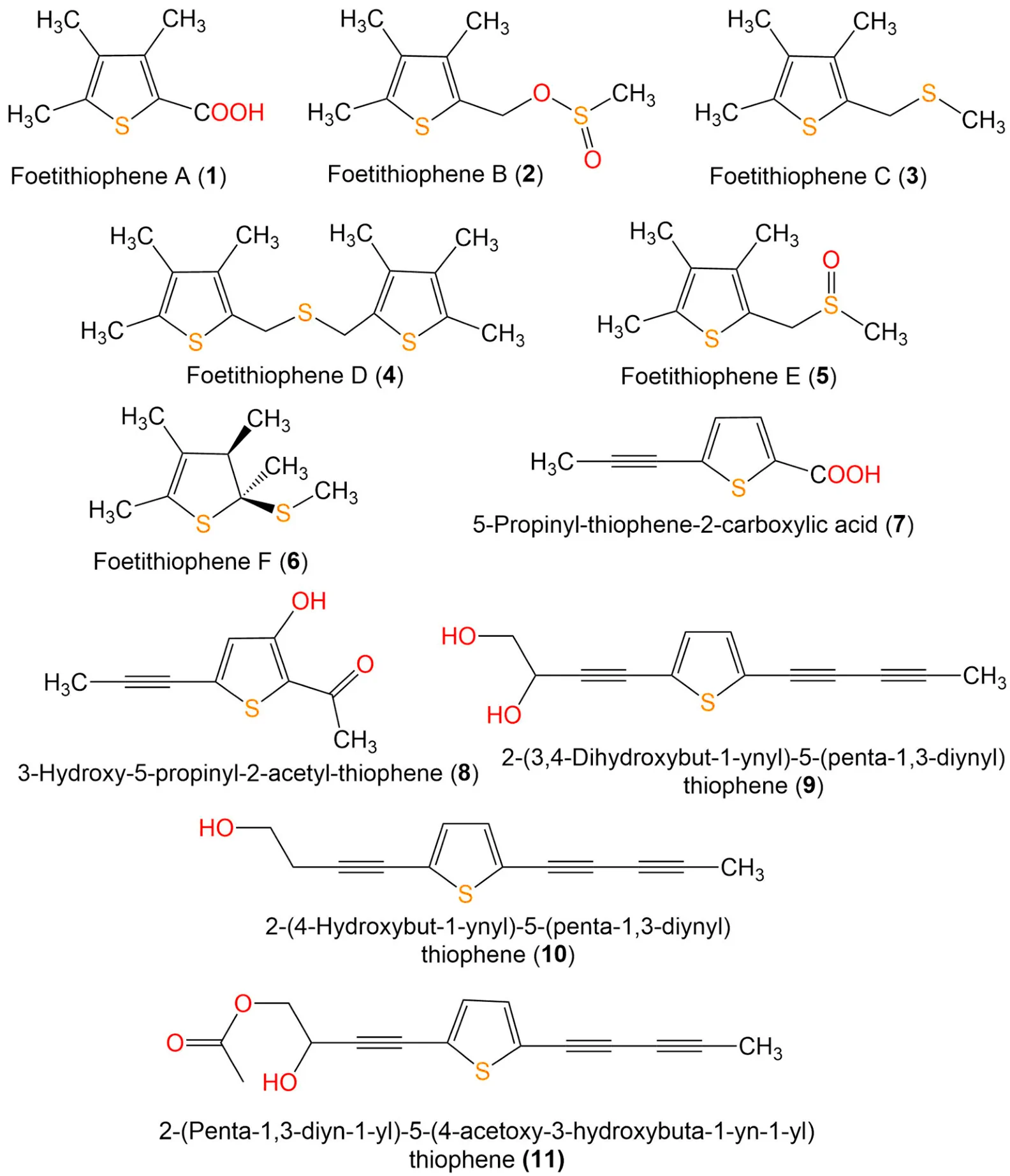
Structures of monothiophenes **1**–**11**.

**Figure 4:**
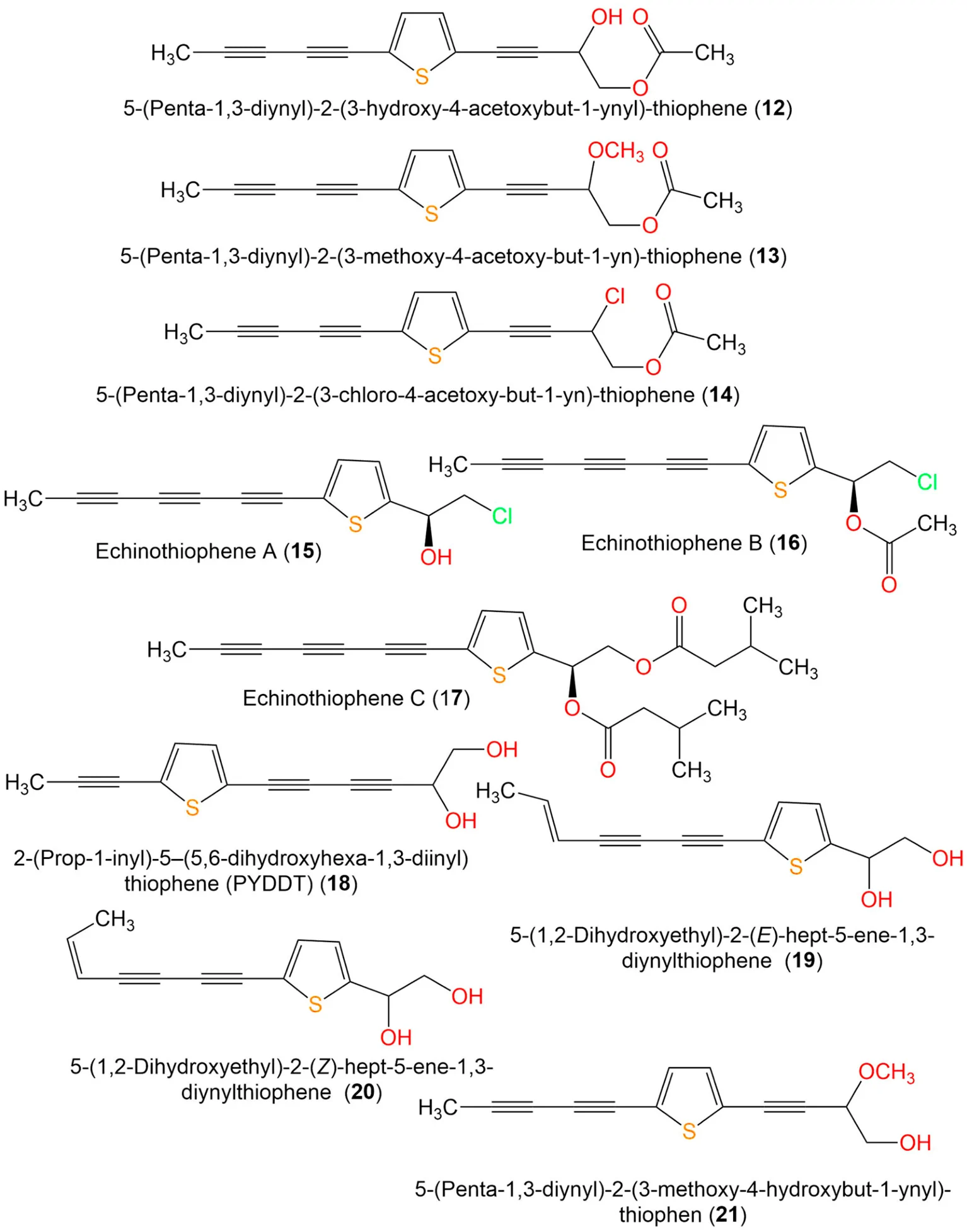
Structures of monothiophenes **12**–**21**.

Zhou et al. reported that **7** and **8** separated from *Artemisia sieversiana* exhibited significant anti-neuroinflammatory activity on the LPS-caused NO production in BV-2 murine microglial cells (half-maximal inhibitory concentrations (IC_50_s) 79.5 and 98.5 µM, respectively), compared to quercetin (IC_50_ 16.3 µM) [26] (Figure 3 and Figure 4).

**Figure 5:**
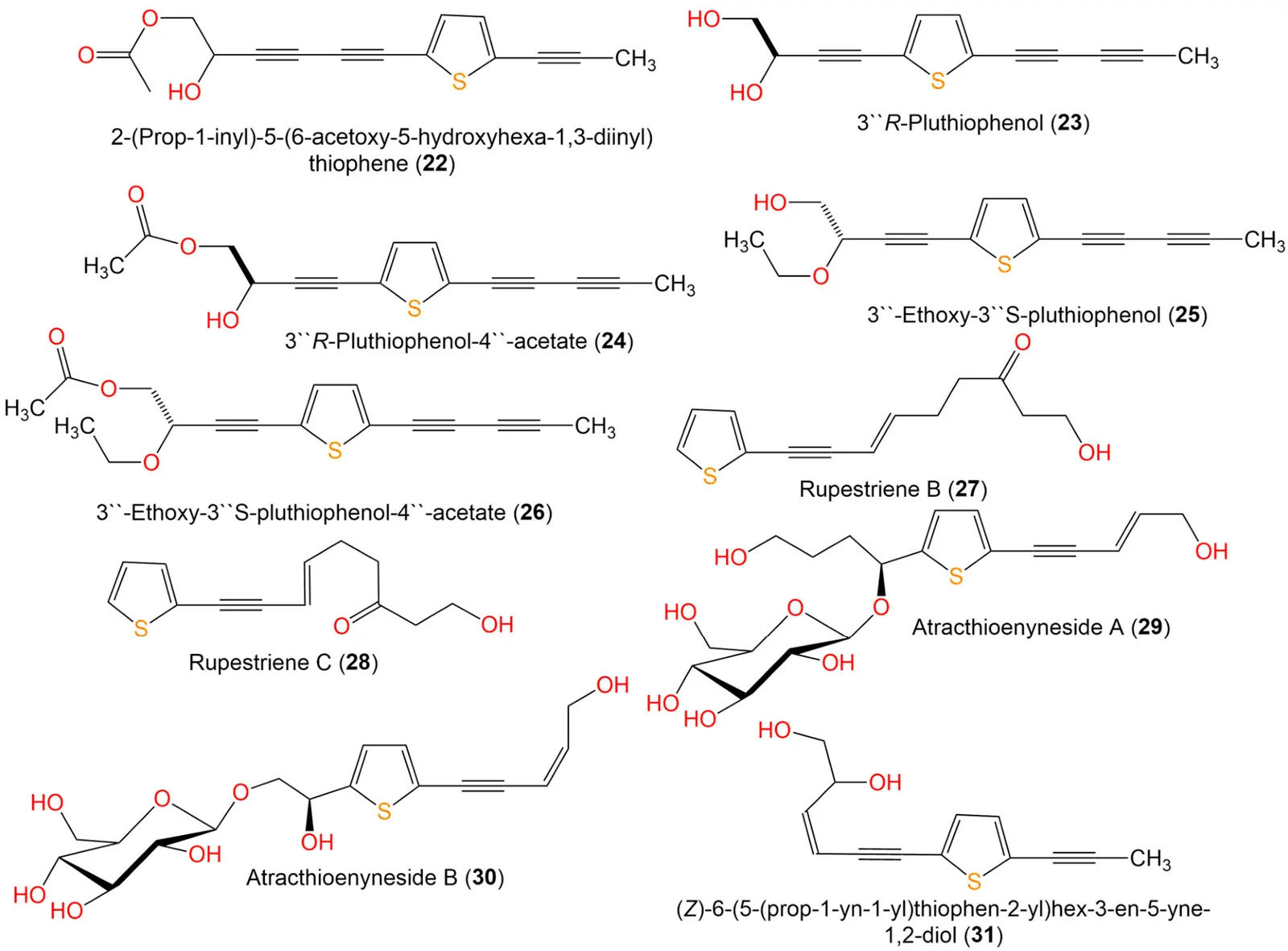
Structures of monothiophenes **22–31**.

In an in vitro anti-inflammatory assay, compounds **23**–**26** obtained from *Pluchea indica* aerial parts possessed significant inhibitory activity against NO production caused by LPS in RAW 264.7 macrophages at a concentration of 40 µM, with % inhibition ranging from 83.4% to 90.1%, compared to dexamethasone (62.2%) [35] (Figure 5).

On the other hand, the two new thiophene polyacetylene glycosides, atracthioenynesides A (**29**) and B (**30**), isolated from *Atractylodes lancea* rhizomes, did not show any activity in LPS-induced NO production in BV-2 cells [37].

**Figure 6:**
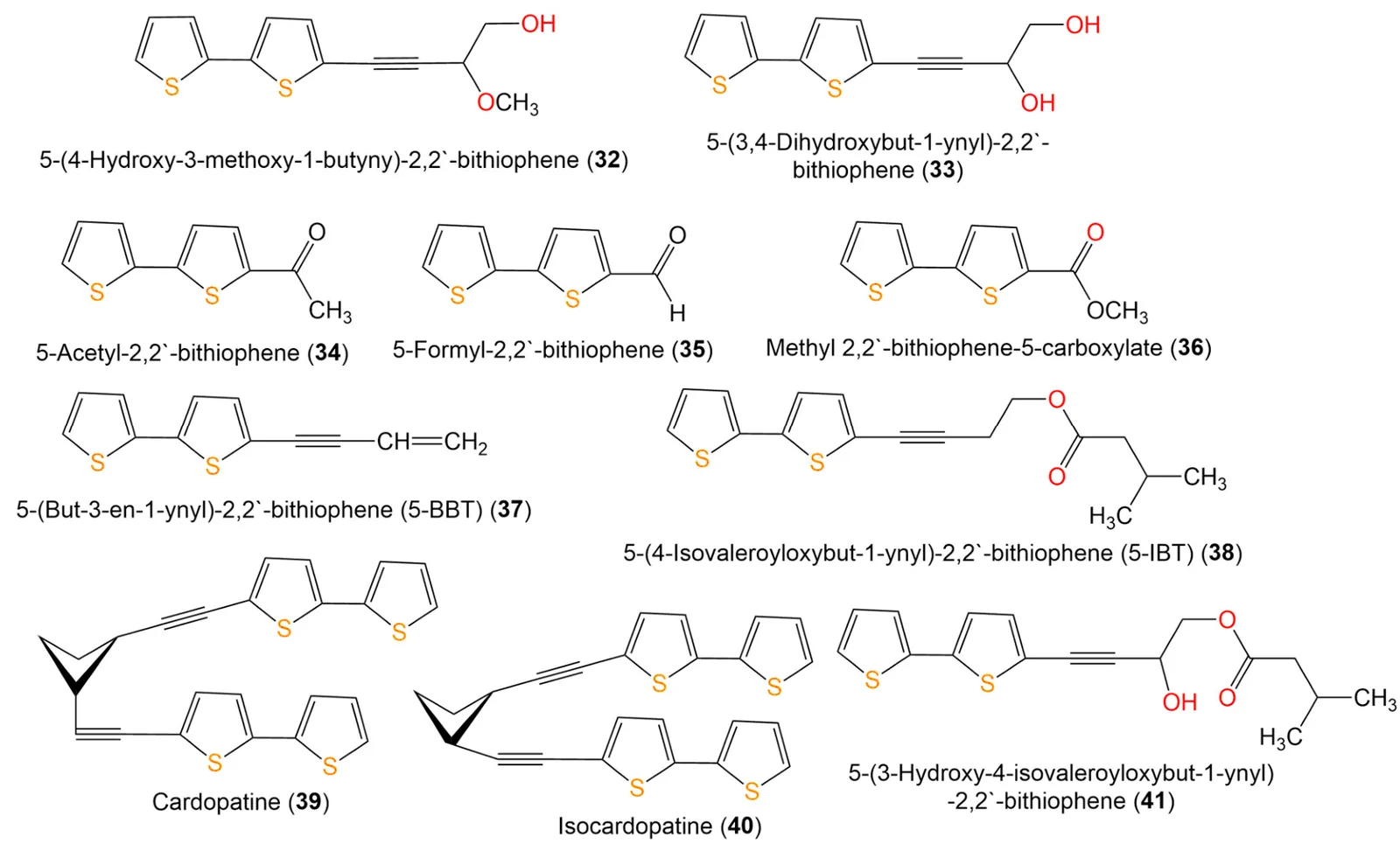
Structures of compounds **32**–**41**.

**Figure 7:**
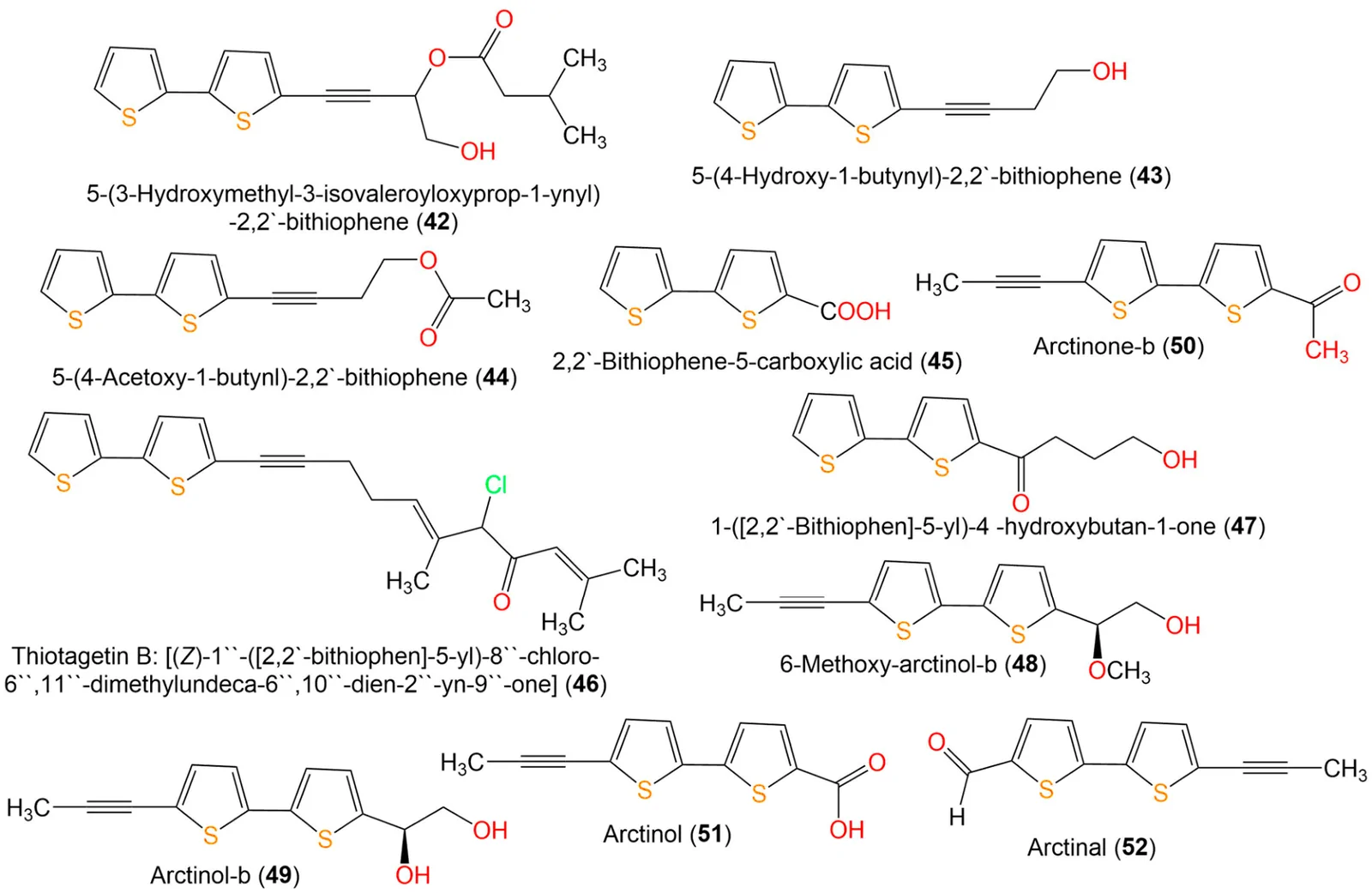
Structures of bithiophenes **42**–**52**.

A new bithiophene, **32**, along with 16 formerly separated thiophenes, **9**, **10**, **33–45**, and **75**, were purified from the EtOAc-soluble fraction of the MeOH extract of *Echinops grijisii* roots using SiO_2_ column chromatography (CC) eluted with n-hexane-EtOAc gradient, as well as HPLC, and identified by IR, UV, NMR, and HRESIMS spectroscopy [28] (Figure 6 and Figure 7).

These compounds were assessed for anti-inflammatory activity versus RAW 264.7 cells. Only **9***,* **33**, and **43** (IC_50_s 2.5, 20.0, and 6.7 µg/mL, respectively) exhibited significant in vitro anti-inflammatory activity against LPS-boosted NO production in RAW 264.7 cells compared to indomethacin (IC_50_ 65.4 µg/mL) in the colorimetric assay [28]. Zhang et al. purified three new derivatives, rupestrienes A–C (**86**, **27**, and **28**), from the EtOH extract of *Artemisia rupestris* using SiO2, RP-18, and Sephadex CC. Rupestrienes B and C (**27** and **28**) displayed significant inhibitory activity (IC_50_ 8.5 and 5.3 μM, respectively) against LPS-caused NO production in BV-2 microglial cells. In contrast, **86** exhibited weak activity (IC_50_ 20.3 μM) compared to quercetin (IC_50_ 4.3 μM) [36]. Jin et al. assessed the inhibitory activity of **19**, **20**, **48**, **49**, **51**, and **55** against NO production boosted by LPS in RAW 264.7 cells. Only **19**, **20**, **48**, and **49** exhibited moderate inhibitory activity (IC_50_ 12.8–48.7 µM), compared to indomethacin and aminoguanidine (IC_50_s 13.2 and 24.2 µM, respectively) (Table 2). On the other hand, **51** and **55** did not exhibit any activity (IC_50_ ˃ 100 µM) [34]. The structure–activity relationship revealed that the monothiophenes with two acetylene units were more potent than the bithiophenes with one acetylene unit. The existence of the Δ^10,11^ cis double bond and 1,2-diol at C-5 enhanced the inhibitory activity [34].

**Table 2:** Biological activities of naturally occurring thiophenes.

Compound Name	Biological Activity	Assay, Organism, or Cell Line	Biological Results		Ref.
			Compound	Positive Control	
Foetithiophene F (**6**)	Antimicrobial	Broth microdilution/*B. cereus*	50 µg/mL (MIC)	Gentamicin 10 µg/mL (MIC)	[25]
5-Propinyl-thiophene-2-carboxylic acid (**7**)	In vitro anti-inflammatory/NO	LPS-stimulated production in BV-2 microglial cells	79.5 µM (IC_50_)	Quercetin 16.3 µM (IC_50_)	[26]
3-Hydroxy-5-propinyl-2-acetyl-thiophene (**8**)	In vitro anti-inflammatory/NO	LPS-stimulated production in BV-2 microglial cells	98.5 µM (IC_50_)	Quercetin 16.3 µM (IC_50_)	[26]
2-(3,4-Dihydroxybut-1-ynyl)-5-(penta-1,3-diynyl)thiophene (**9**)	In vitro anti-inflammatory/NO	LPS-stimulated production in the RAW 264.7 cell line	2.5 µg/mL (IC_50_)	Indomethacin 65.4 µg/mL (IC_50_)	[28]
	Cytotoxicity	Resazurin reduction/CEM/ADR5000	21.09 µM (IC_50_)	Doxorubicin 195.12 µM (IC_50_)	[30]
	Cytotoxicity	Resazurin reduction/CCRF-CEM	46.96 µM (IC_50_)	Doxorubicin 0.20 µM (IC_50_)	[30]
	Antimicrobial	INT/*E. coli*	64.0 µg/mL (MIC)	Chloramphenicol 64.0 µg/mL (MIC)	[29]
	Antimicrobial	INT/*E. aerogenes*	64.0 µg/mL (MIC)	Chloramphenicol 16.0 µg/mL (MIC)	[29]
	Antimicrobial	INT/*K. pneumoniae*	64.0 µg/mL (MIC)	Chloramphenicol 16.0 µg/mL (MIC)	[29]
	Antimicrobial	INT/*P. stuartil*	64.0 µg/mL (MIC)	Chloramphenicol 128.0 µg/mL (MIC)	[29]
	Antimicrobial	INT/*E. cloacae*	256.0 µg/mL (MIC)	Chloramphenicol 256.0 µg/mL (MIC)	[29]
	Antimicrobial	INT/*P. aeruginosa*	256.0 µg/mL (MIC)	Chloramphenicol 16.0 µg/mL (MIC)	[29]
2-(Penta-1,3-diyn-1-yl)-5–(4-acetoxy-3-hydroxybuta-1-yn-1-yl) thiophene (**11**)	CYP2A*6* inhibition	Enzymatic reconstitution	6.43 µM (IC_50_)	Methoxsalen 0.19 µM (IC_50_)	[31]
	CYP2A13 inhibition	Enzymatic reconstitution	6.18 µM (IC_50_)	Methoxsalen 0.43 µM (IC_50_)	[31]
Echinothiophene A (**15**)	Nematicidal	Nematode Mortality/J2s of *Meloidogyne incognita*	0.42 µg/mL (LC_50_) in light	Abamectin 8.73 (LC_50_) in light	[19]
			1.44 µg/mL (LC_50_) in dark	Abamectin 9.38 (LC_50_) in dark	[19]
	Antifungal	Broth microdilution/*Fusarium solani*	64.0 µg/mL (MIC)	Carbendazim 0.5 µg/mL (MIC)	[19]
		Broth microdilution/*F*. *oxysporum* f. sp. *vasinfectum*	16.0 µg/mL (MIC)	Carbendazim 2.0 µg/mL (MIC)	[19]
		*Broth microdilution/F. oxysporum* f. sp. *niveum*	8.0 µg/mL (MIC)	Carbendazim 8.0 µg/mL (MIC)	[19]
		Broth microdilution/*Phytophthora infestans*	128.0 µg/mL (MIC)	Carbendazim 256.0 µg/mL (MIC)	[19]
		Broth microdilution/*Colletotrichum gloeosporioides*	16.0 µg/mL (MIC)	Carbendazim 8.0 µg/mL (MIC)	[19]
		Broth microdilution/*Alternaria alternata*	4.0 µg/mL (MIC)	Carbendazim 16.0 µg/mL (MIC)	[19]
Echinothiophene B (**16**)	Nematicidal	Nematode Mortality/J2s of *Meloidogyne incognita*	2.65 µg/mL (LC_50_) in light	Abamectin 8.73 (LC_50_) in light	[19]
			9.23 µg/mL (LC_50_) in dark	Abamectin 9.38 (LC_50_) in dark	[19]
	Antifungal	Broth microdilution/*Fusarium solani*	32.0 µg/mL (MIC)	Carbendazim 0.5 µg/mL (MIC)	[19]
		Broth microdilution/*F. oxysporum* f. sp. *vasinfectum*	64.0 µg/mL (MIC)	Carbendazim 2.0 µg/mL (MIC)	[19]
		Broth microdilution/*F. oxysporum* f. sp. *niveum*	16.0 µg/mL (MIC)	Carbendazim 8.0 µg/mL (MIC)	[19]
		Broth microdilution/*Phytophthora infestans*	256.0 µg/mL (MIC)	Carbendazim 256.0 µg/mL (MIC)	[19]
		Broth microdilution/*Colletotrichum gloeosporioides*	8.0 µg/mL (MIC)	Carbendazim 8.0 µg/mL (MIC)	[19]
		Broth microdilution/*Alternaria alternataalternata*	8.0 µg/mL (MIC)	Carbendazim 16.0 µg/mL (MIC)	[19]
Echinothiophene C (**17**)	Nematicidal	Nematode Mortality/J2s of *Meloidogyne incognita*	16.55 µg/mL (LC_50_) in light	Abamectin 8.73 (LC_50_) in light	[19]
			18.17 µg/mL (LC_50_) in dark	Abamectin 9.38 (LC_50_) in dark	[19]
	Antifungal	Broth microdilution/*F. oxysporum* f. sp. *vasinfectum*	128.0 µg/mL (MIC)	Carbendazim 2.0 µg/mL (MIC)	[19]
		Broth microdilution/*F. oxysporum* f. sp. *niveum*	256.0 µg/mL (MIC)	Carbendazim 8.0 µg/mL (MIC)	[19]
		Broth microdilution/*Colletotrichum gloeosporioides*	128.0 µg/mL (MIC)	Carbendazim 8.0 µg/mL (MIC)	[19]
		Broth microdilution/*Alternaria alternataalternata*	32.0 µg/mL (MIC)	Carbendazim 16.0 µg/mL (MIC)	[19]
2-(Pro-1-ynyl)-5-(5,6-dihydroxypenta-1,3-diynyl) thiophene (PYDDT) (**18**)	CYP2A6 inhibition	Enzymatic reconstitution	3.90 µM (IC_50_)	Methoxsalen 0.19 µM (IC_50_)	[31]
	CYP2A13 inhibition	Enzymatic reconstitution	2.40 µM (IC_50_)	Methoxsalen 0.43 µM (IC_50_)	[31]
5-(1,2-Dihydroxyethyl)-2-(*E*)-hept-5-ene-1,3-diynylthiophene (**19**)	In vitro anti-inflammatory/NO	LPS-stimulated production in the RAW 264.7 cell line	28.2 µM (IC_50_)	-Indomethacin 13.2 µM (IC_50_) -Aminoguanidine 24.2 µM (IC_50_)	[34]
5-(1,2-Dihydroxy-ethyl)-2-(*Z*)-hept-5-ene-1,3-diynylthiophene (**20**)	In vitro anti-inflammatory/NO	LPS-stimulated production in the RAW 264.7 cell line	12.8 µM (IC_50_)	-Indomethacin 13.2 µM (IC_50_) -Aminoguanidine 24.2 µM (IC_50_)	[34]
2-(Prop-1-inyl)-5-(6-acetoxy-5-hydroxyhexa-1,3-diinyl) thiophene (**22**)	CYP2A6 inhibition	Enzymatic reconstitution	4.44 µM (IC_50_)	Methoxsalen 0.19 µM (IC_50_)	[31]
	CYP2A13 inhibition	Enzymatic reconstitution	2.94 µM (IC_50_)	Methoxsalen 0.43 µM (IC_50_)	[31]
3′′*R*-Pluthiophenol (**23**)	In vitro anti-inflammatory/NO	LPS-stimulated production in RAW 264.*7* macrophages cells	84.5 (NRC % inhibition)	Dexamethasone 62.2 (NRC % inhibition)	[35]
3′′*R*-Pluthiophenol-4′′-acetate (**24**)	In vitro anti-inflammatory/NO	LPS-stimulated production in RAW 264.7 macrophages cells	83.4 (NRC % inhibition)	Dexamethasone 62.2 (NRC % inhibition)	[35]
3′′-Ethoxy-3′′*S*-pluthiophenol (**25**)	In vitro anti-inflammatory/NO	LPS-stimulated production in RAW 264.7 macrophages cells	86.9 (NRC % inhibition)	Dexamethasone 62.2 (NRC % inhibition)	[35]
3′′-Ethoxy-3′′*S*-pluthiophenol-4′′-acetate (**26**)	In vitro anti-inflammatory/*NO*	LPS-stimulated production in RAW 264.7 macrophages cells	90.1 (NRC % inhibition)	Dexamethasone 62.2 (NRC % inhibition)	[35]
Rupestriene B (**27**)	In vitro anti-inflammatory/*NO*	LPS-stimulated production in BV-2 microglial cells	8.5 µM (IC_50_)	Quercetin 4.3 µM (IC_50_)	[36]
Rupestriene C (**28**)	In vitro anti-inflammatory/*NO*	LPS-stimulated production in BV-2 microglial cells	5.3 µM (IC_50_)	Quercetin 4.3 µM (IC_50_)	[36]
5-(3,4-Dihydroxybut-1-ynyl)-2,2′-bithiophene (33)	In vitro anti-inflammatory/*NO*	LPS-stimulated production in the RAW 264.7 cell line	20.0 µg/mL (IC_50_)	Indomethacin 65.4 µg/mL (IC_50_)	[28]
5-(But-3-en-1-ynyl)-2,2′-bithiophene (5-BBT) (37)	Fungicidal	Broth microdilution/*C. albicans* ATCC 10231	7.81 µg/mL (MFC) in light	Amphotericin B 0.50 µg/mL (MFC) Fluconazole ˃ 64 µg/mL (MFC) Itraconazole ˃ 16 µg/mL (MFC)	[40]
	Larvicidal	Larval mortality/Aedes albopictus	0.34 µg/mL (LC_50_)	Rotenone 3.75 µg/mL (LC_50_)	[41]
	Larvicidal	Larval mortality/Aedes albopictus	0.72 µg/mL (LC_95_)	Rotenone 9.45 µg/mL (LC_95_)	[41]
	Larvicidal	Larval mortality/Anopheles sinensis	1.36 µg/mL (LC_50_)	Rotenone 1.25 µg/mL (LC_50_)	[41]
	Larvicidal	Larval mortality/Anopheles sinensis	1.93 µg/mL (LC_95_)	Rotenone 2.24 µg/mL (LC_95_)	[41]
	Larvicidal	Larval mortality/Culex pipiens pallens	0.12 µg/mL (LC_50_)	Rotenone 1.88 µg/mL (LC_50_)	[41]
	Fungicidal	Larval mortality/Culex pipiens pallens	0.18 µg/mL (LC_95_)	Rotenone 3.74 µg/mL (LC_95_)	[41]
	Fungicidal	Broth microdilution/*C. albicans* ATCC 10231	62.50 µg/mL (MFC) in low oxygen and light	-	[59]
	Fungicidal	Broth microdilution/*C. albicans* ATCC 10231	7.81 µg/mL (MFC) in normal oxygen and light	-	[59]
5-(4-Isovaleroyloxybut-1-ynyl)-2,2′-bithiophene (5-IBT) (**38**)	Larvicidal	Larval mortality/*Aedes albopictus*	0.45 µg/mL (LC_50_)	Rotenone 3.75 µg/mL (LC_50_)	[41]
	Larvicidal		0.66 µg/mL (LC_95_)	Rotenone 9.45 µg/mL (LC_95_)	[41]
	Larvicidal	Larval mortality/*Anopheles sinensis*	5.36 µg/mL (LC_50_)	Rotenone 1.25 µg/mL (LC_50_)	[41]
	Larvicidal		11.26 µg/mL (LC_95_)	Rotenone 2.24 µg/mL (LC_95_)	[41]
	Larvicidal	Larval mortality/*Culex pipiens* pallens	0.33 µg/mL (LC_50_)	Rotenone 1.88 µg/mL (LC_50_)	[41]
	Larvicidal		0.54 µg/mL (LC_95_)	Rotenone 3.74 µg/mL (LC_95_)	[41]
5-(4-Hydroxy-1-butynyl)-2,2′-bithiophene (**43**)	In vitro anti-inflammatory/NO	LPS-stimulated production in the RAW 264.7 cell line	6.7 µg/mL (IC_50_)	Indomethacin 65.4 µg/mL (IC_50_)	[28]
	Fungicidal	Broth microdilution/*C. albicans* ATCC 10231	3.90 µg/mL (MFC) in light	-Amphotericin B 0.50 µg/mL (MFC) -Fluconazole ˃ 64 µg/mL (MFC) -Itraconazole ˃ 16 µg/mL (MFC)	[40]
	Antimicrobial	Broth microdilution/*S. aureus* ATCC 2592	8.0 µg/mL (MIC)	Levofloxacin 8.0 μg/mL (MIC)	[27]
	Antimicrobial	Broth microdilution/*E. coli* ATCC 25922	64.0 µg/mL (MIC)	Levofloxacin 16.0 μg/mL (MIC)	[27]
	Antimicrobial	Broth microdilution/*C. albicans* ATCC2002	64.0 µg/mL (MIC)	Levofloxacin 64.0 μg/mL (MIC)	[27]
	Fungicidal	Broth microdilution/*C. albicans* ATCC 10231	250.0 µg/mL (MFC) in low oxygen and light	-	[59]
	Fungicidal	Broth microdilution/*C. albicans* ATCC 10231	3.90 µg/mL (MFC) in normal oxygen and light	-	[59]
	Anti-inflammatory	Colorimetric/5-LOX	41.82 µM (IC_50_)	Indomethacin 0.89 µM (IC_50_)	[43]
5-(4-Acetoxy-1-butynl)-2,2′-bithiophene (**44**)	Fungicidal	Broth microdilution/*C. albicans* ATCC 10231	7.81 µg/mL (MFC) in light	-Amphotericin B 0.50 µg/mL (MFC) -Fluconazole ˃ 64 µg/mL (MFC) -Itraconazole ˃ 16 µg/mL (MFC)	[40]
	Fungicidal	Broth microdilution/*C. albicans* ATCC 10231	62.50 µg/mL (MFC) in low oxygen and light	-	[59]
	Fungicidal	Broth microdilution/*C. albicans* ATCC 10231	7.81 µg/mL (MFC) in normal oxygen and light	-	[59]
6-Methoxy-arctinol-b (**48**)	In vitro anti-inflammatory/NO	LPS-stimulated production in the RAW 264.7 cell line	30.6 µM (IC_50_)	-Indomethacin 13.2 µM (IC_50_) -Aminoguanidine 24.2 µM (IC_50_)	[34]
	Nematicidal	Nematode Mortality/J2s of *Meloidogyne incognita*	5.83 µg/mL (LC_50_) in light	Abamectin 8.73 (LC_50_) in light	[19]
			7.05 µg/mL (LC_50_) in dark	Abamectin 9.38 (LC_50_) in dark	[19]
	Antifungal	Broth microdilution/*Fusarium solani*	128.0 µg/mL (MIC)	Carbendazim 0.5 µg/mL (MIC)	[19]
		Broth microdilution/*F. oxysporum* f. sp. *vasinfectum*	256.0 µg/mL (MIC)	Carbendazim 2.0 µg/mL (MIC)	[19]
		Broth microdilution/*F. oxysporum* f. sp. *niveum*	128.0 µg/mL (MIC)	Carbendazim 8.0 µg/mL (MIC)	[19]
		Broth microdilution/*Colletotrichum gloeosporioides*	32.0 µg/mL (MIC)	Carbendazim 8.0 µg/mL (MIC)	[19]
		Broth microdilution/*Alternaria alternataalternata*	32.0 µg/mL (MIC)	Carbendazim 16.0 µg/mL (MIC)	[19]
Arctinol-b (**49**)	In vitro anti-inflammatory/NO	LPS-stimulated production in the RAW 264.7 cell line	48.7 µM (IC_50_)	-Indomethacin 13.2 µM (IC_50_) -Aminoguanidine 24.2 µM (IC_50_)	[34]
	Antimicrobial	Broth microdilution/*S. aureus* ATCC 2592	8.0 µg/mL (MIC)	Levofloxacin 8.0 μg/mL (MIC)	[27]
	Antimicrobial	Broth microdilution/*E. coli* ATCC 25922	64.0 µg/mL (MIC)	Levofloxacin 16.0 μg/mL (MIC)	[27]
	Antimicrobial	Broth microdilution/*C. albicans* ATCC2002	64.0 µg/mL (MIC)	Levofloxacin 64.0 μg/mL (MIC)	[27]
	Nematicidal	Nematode Mortality/J2s of *Meloidogyne incognita*	13.48 µg/mL (LC_50_) in light	Abamectin 8.73 (LC_50_) in light	[19]
			14.72 µg/mL (LC_50_) in dark	Abamectin 9.38 (LC_50_) in dark	[19]
	Antifungal	Broth microdilution/*F. oxysporum* f. sp. *vasinfectum*	256.0 µg/mL (MIC)	Carbendazim 2.0 µg/mL (MIC)	[19]
		Broth microdilution/*F. oxysporum* f. sp. *niveum*	64.0 µg/mL (MIC)	Carbendazim 8.0 µg/mL (MIC)	[19]
		Broth microdilution/*Phytophthora infestans*	128.0 µg/mL (MIC)	Carbendazim 256.0 µg/mL (MIC)	[19]
		Broth microdilution/*Colletotrichum gloeosporioides*	32.0 µg/mL (MIC)	Carbendazim 8.0 µg/mL (MIC)	[19]
		Broth microdilution/*Alternaria alternata*	64.0 µg/mL (MIC)	Carbendazim 16.0 µg/mL (MIC)	[19]
Arctinone-b (**50**)	Nematicidal	Nematode Mortality/J2s of *Meloidogyne incognita*	1.14 µg/mL (LC_50_) in light	Abamectin 8.73 (LC_50_) in light	[19]
			2.00 µg/mL (LC_50_) in dark	Abamectin 9.38 (LC_50_) in dark	[19]
	Antifungal	Broth microdilution/*F. oxysporum* f. sp. *vasinfectum*	256.0 µg/mL (MIC)	Carbendazim 2.0 µg/mL (MIC)	[19]
		Broth microdilution/*Colletotrichum gloeosporioides*	64.0 µg/mL (MIC)	Carbendazim 8.0 µg/mL (MIC)	[19]
		Broth microdilution/*Alternaria alternata*	128.0 µg/mL (MIC)	Carbendazim 16.0 µg/mL (MIC)	[19]
Arctinol (**51**)	Nematicidal	Nematode Mortality/J2s of *Meloidogyne incognita*	15.90 µg/mL (LC_50_) in light	Abamectin 8.73 (LC_50_) in light	[19]
			17.82 µg/mL (LC_50_) in dark	Abamectin 9.38 (LC_50_) in dark	[19]
	Antifungal	Broth microdilution/*F*. *oxysporum* f. sp. *vasinfectum*	256.0 µg/mL (MIC)	Carbendazim 2.0 µg/mL (MIC)	[19]
		Broth microdilution/*F. oxysporum* f. sp. *niveum*	128.0 µg/mL (MIC)	Carbendazim 8.0 µg/mL (MIC)	[19]
		Broth microdilution/*Phytophthora infestans*	128.0 µg/mL (MIC)	Carbendazim 256.0 µg/mL (MIC)	[19]
		Broth microdilution/*Colletotrichum gloeosporioides*	32.0 µg/mL (MIC)	Carbendazim 8.0 µg/mL (MIC)	[19]
		Broth microdilution/*Alternaria alternata*	16.0 µg/mL (MIC)	Carbendazim 16.0 µg/mL (MIC)	[19]
Arctinal (**52**)	Antimicrobial	Broth microdilution/*S. aureus* ATCC 2592	32.0 µg/mL (MIC)	Levofloxacin 8.0 μg/mL (MIC)	[19]
	Antimicrobial	Broth microdilution/*E. coli* ATCC 25922	64.0 µg/mL (MIC)	Levofloxacin 16.0 μg/mL (MIC)	[19]
	Nematicidal	Nematode Mortality/J2s of *Meloidogyne incognita*	2.62 µg/mL (LC_50_) in light	Abamectin 8.73 (LC_50_) in light	[19]
			8.75 µg/mL (LC_50_) in dark	Abamectin 9.38 (LC_50_) in dark	[19]
	Antifungal	Broth microdilution/*F*. *oxysporum* f. sp. *vasinfectum*	64.0 µg/mL (MIC)	Carbendazim 2.0 µg/mL (MIC)	[19]
		Broth microdilution/*F. oxysporum* f. sp. *niveum*	128.0 µg/mL (MIC)	Carbendazim 8.0 µg/mL (MIC)	[19]
		Broth microdilution/*Colletotrichum gloeosporioides*	32.0 µg/mL (MIC)	Carbendazim 8.0 µg/mL (MIC)	[19]
		Broth microdilution/*Alternaria alternata*	64.0 µg/mL (MIC)	Carbendazim 16.0 µg/mL (MIC)	[19]
Arctinol A (**53**)	Antimicrobial	Broth microdilution/*S. aureus* ATCC 2592	8.0 µg/mL (MIC)	Levofloxacin 8.0 μg/mL (MIC)	[27]
	Antimicrobial	Broth microdilution/*E. coli* ATCC 25922	64.0 µg/mL (MIC)	Levofloxacin 16.0 μg/mL (MIC)	[27]
5′-(3,4-Dihydroxybut-1-yn-1-yl)-[2,2′-bithiophene]-5-carbaldehyde (**57**)	Antimicrobial	Broth microdilution/*S. aureus* ATCC 2592	128.0 µg/mL (MIC)	Levofloxacin 8.0 μg/mL (MIC)	[27]
	Antimicrobial	Broth microdilution/*E. coli* ATCC 25922	256.0 µg/mL (MIC)	Levofloxacin 16.0 μg/mL (MIC)	[27]
	Antimicrobial	Broth microdilution/*C. albicans* ATCC2002	256.0 µg/mL (MIC)	Levofloxacin 64.0 μg/mL (MIC)	[27]
4-Hydroxy-1-(5′-methyl-[2,2′-bithiophen]-5-yl)butan-1-one (**58**)	Antimicrobial	Broth microdilution/*S. aureus* ATCC 2592	8.0 µg/mL (MIC)	Levofloxacin 8.0 μg/mL (MIC)	[27]
	Antimicrobial	Broth microdilution/*E. coli* ATCC 25922	32.0 µg/mL (MIC)	Levofloxacin 16.0 μg/mL (MIC)	[27]
	Antimicrobial	*Broth microdilution/C. albicans* ATCC2002	32.0 µg/mL (MIC)	Levofloxacin 64.0 μg/mL (MIC)	[27]
5′-(3,4-Dihydroxybut-1-yn-1-yl)-[2,2′-bithiophene]-5-carboxylic acid (**59**)	Antimicrobial	*Broth microdilution/S. aureus* ATCC 2592	256.0 µg/mL (MIC)	Levofloxacin 8.0 μg/mL (MIC)	[27]
Echinothiophene D (**61**)	Nematicidal	Nematode Mortality/J2s of *Meloidogyne incognita*	2.57 µg/mL (LC_50_) in light	Abamectin 8.73 (LC_50_) in light	[19]
			1.80 µg/mL (LC_50_) in dark	Abamectin 9.38 (LC_50_) in dark	[19]
	Antifungal	Broth microdilution/*Fusarium solani*	32.0 µg/mL (MIC)	Carbendazim 0.5 µg/mL (MIC)	[19]
		Broth microdilution/*F*. *oxysporum* f. sp. *vasinfectum*	128.0 µg/mL (MIC)	Carbendazim 2.0 µg/mL (MIC)	[19]
		Broth microdilution/*F. oxysporum* f. sp. *niveum*	32.0 µg/mL (MIC)	Carbendazim 8.0 µg/mL (MIC)	[19]
		Broth microdilution/*Phytophthora infestans*	256.0 µg/mL (MIC)	Carbendazim 256.0 µg/mL (MIC)	[19]
		Broth microdilution/*Colletotrichum gloeosporioides*	8.0 µg/mL (MIC)	Carbendazim 8.0 µg/mL (MIC)	[19]
		Broth microdilution/*Alternaria alternata*	16.0 µg/mL (MIC)	Carbendazim 16.0 µg/mL (MIC)	[19]
Echinothiophene E (**62**)	Nematicidal	Nematode Mortality/J2s of *Meloidogyne incognita*	8.28 µg/mL (LC_50_) in light	Abamectin 8.73 (LC_50_) in light	[19]
			9.12 µg/mL (LC_50_) in dark	Abamectin 9.38 (LC_50_) in dark	[19]
	Antifungal	Broth microdilution/*Fusarium solani*	64.0 µg/mL (MIC)	Carbendazim 0.5 µg/mL (MIC)	[19]
		Broth microdilution/*F*. *oxysporum* f. sp. *vasinfectum*	32.0 µg/mL (MIC)	Carbendazim 2.0 µg/mL (MIC)	[19]
		Broth microdilution/*F. oxysporum* f. sp. *niveum*	128.0 µg/mL (MIC)	Carbendazim 8.0 µg/mL (MIC)	[19]
		Broth microdilution/*Phytophthora infestans*	256.0 µg/mL (MIC)	Carbendazim 256.0 µg/mL (MIC)	[19]
		Broth microdilution/*Colletotrichum gloeosporioides*	32.0 µg/mL (MIC)	Carbendazim 8.0 µg/mL (MIC)	[19]
		Broth microdilution/*Alternaria alternata*	16.0 µg/mL (MIC)	Carbendazim 16.0 µg/mL (MIC)	[19]
Echinothiophene F (**63**)	Nematicidal	Nematode Mortality/J2s of *Meloidogyne incognita*	20.13 µg/mL (LC_50_) in light	Abamectin 8.73 (LC_50_) in light	[19]
			18.41 µg/mL (LC_50_) in dark	Abamectin 9.38 (LC_50_) in dark	[19]
	Antifungal	Broth microdilution/*F. oxysporum* f. sp. *niveum*	128.0 µg/mL (MIC)	Carbendazim 8.0 µg/mL (MIC)	[19]
		Broth microdilution/*Colletotrichum gloeosporioides*	256.0 µg/mL (MIC)	Carbendazim 8.0 µg/mL (MIC)	[19]
		Broth microdilution/*Alternaria alternata*	64.0 µg/mL (MIC)	Carbendazim 16.0 µg/mL (MIC)	[19]
2-Prop-1-inyl-5′-(2-hydroxy-3-chloropropyl) dithiophene (**64**)	Nematicidal	Nematode Mortality/J2s of *Meloidogyne incognita*	0.91 µg/mL (LC_50_) in light	Abamectin 8.73 (LC_50_) in light	[19]
			0.86 µg/mL (LC_50_) in dark	Abamectin 9.38 (LC_50_) in dark	[19]
	Antifungal	Broth microdilution/*Fusarium solani*	64.0 µg/mL (MIC)	Carbendazim 0.5 µg/mL (MIC)	[19]
		Broth microdilution/*F*. *oxysporum* f. sp. *vasinfectum*	32.0 µg/mL (MIC)	Carbendazim 2.0 µg/mL (MIC)	[19]
		Broth microdilution/*F. oxysporum* f. sp. *niveum*	4.0 µg/mL (MIC)	Carbendazim 8.0 µg/mL (MIC)	[19]
		Broth microdilution/*Phytophthora infestans*	32.0 µg/mL (MIC)	Carbendazim 256.0 µg/mL (MIC)	[19]
		Broth microdilution/*Colletotrichum gloeosporioides*	4.0 µg/mL (MIC)	Carbendazim 8.0 µg/mL (MIC)	[19]
		Broth microdilution/*Alternaria alternata*	4.0 µg/mL (MIC)	Carbendazim 16.0 µg/mL (MIC)	[19]
Ecliprostin A (**65**)	Antibacterial	Broth microdilution/*S. aureus*	25.0 µM (MIC)	Penicillin 0.156 µM (MIC)	[18]
Ecliprostin B (**66**)	Antibacterial	Broth microdilution/*S. aureus*	6.25 µM (MIC)	Penicillin 0.156 µM (MIC)	[18]
Ecliprostin C (**67**)	Antibacterial	Broth microdilution/*S. aureus*	25.0 µM (MIC)	Penicillin 0.156 µM (MIC)	[18]
Echinbithiophene dimer A (**68**)	Nematicidal	Nematode Mortality/J2s of *Meloidogyne incognita*	16.53 µg/mL (LC_50_) in light	Ethoprophos 36.15 (LC_50_) in light *α*-Terthienyl 0.62 (LC_50_) in light	[17]
			18.17 µg/mL (LC_50_) in dark	Ethoprophos 31.94 (LC_50_) in dark *α*-Terthienyl 2.23 (LC_50_) in dark	[17]
	Antifungal	Broth microdilution/*Alternaria alternata;*	16.0 µg/mL (MIC)	Carbendazim 16.0 µg/mL (MIC)	[17]
		Broth microdilution/*Pyricularia oryzae*	16.0 µg/mL (MIC)	Carbendazim 8.0 µg/mL (MIC)	[17]
		Broth microdilution/*Fusarium oxysporum*	32.0 µg/mL (MIC)	Carbendazim 8.0 µg/mL (MIC)	[17]
		Broth microdilution/*Colletotrichum gloeosporioides*	64.0 µg/mL (MIC)	Carbendazim 2.0 µg/mL (MIC)	[17]
		Broth microdilution/*Phytophthora infestans*	128.0 µg/mL (MIC)	Carbendazim 256.0 µg/mL (MIC)	[17]
Echinbithiophene dimer B (**69**)	Nematicidal	Nematode Mortality/J2s of *Meloidogyne incognita*	13.88 µg/mL (LC_50_) in light	Ethoprophos 36.15 (LC_50_) in light *α*-Terthienyl 0.62 (LC_50_) in light	[17]
			16.28 µg/mL (LC_50_) in dark	Ethoprophos 31.94 (LC_50_) in dark *α*-Terthienyl 2.23 (LC_50_) in dark	[17]
	Antifungal	Broth microdilution/*Alternaria alternata*	16.0 µg/mL (MIC)	Carbendazim 16.0 µg/mL (MIC)	[17]
		Broth microdilution/*Pyricularia oryzae*	16.0 µg/mL (MIC)	Carbendazim 8.0 µg/mL (MIC)	[17]
		Broth microdilution/*Fusarium oxysporum*	16.0 µg/mL (MIC)	Carbendazim 8.0 µg/mL (MIC)	[17]
		Broth microdilution/*Colletotrichum gloeosporioides*	32.0 µg/mL (MIC)	Carbendazim 2.0 µg/mL (MIC)	[17]
		Broth microdilution/*Phytophthora infestans*	128.0 µg/mL (MIC)	Carbendazim 256.0 µg/mL (MIC)	[17]
Echinbithiophenedimer C (**70**)	Nematicidal	Nematode Mortality/J2s of *Meloidogyne incognita*	8.73 µg/mL (LC_50_) in light	Ethoprophos 36.15 (LC_50_) in light *α*-Terthienyl 0.62 (LC_50_) in light	[17]
			9.39 µg/mL (LC_50_) in dark	Ethoprophos 31.94 (LC_50_) in dark *α*-Terthienyl 2.23 (LC_50_) in dark	[17]
	Antifungal	Broth microdilution/*Alternaria alternata;*	8.0 µg/mL (MIC)	Carbendazim 16.0 µg/mL (MIC)	[17]
		Broth microdilution/*Pyricularia oryzae*	8.0 µg/mL (MIC)	Carbendazim 8.0 µg/mL (MIC)	[17]
		Broth microdilution/*Fusarium oxysporum*	32.0 µg/mL (MIC)	Carbendazim 8.0 µg/mL (MIC)	[17]
		Broth microdilution/*Colletotrichum gloeosporioides*	32.0 µg/mL (MIC)	Carbendazim 2.0 µg/mL (MIC)	[17]
		Broth microdilution/*Phytophthora infestans*	128.0 µg/mL (MIC)	Carbendazim 256.0 µg/mL (MIC)	[17]
4-(5′-(hydroxymethyl)-[2,2′-bithiophene]-5-yl)but-3-yn-1-ol) (Thio1) (**74**)	Anthelmintic	Larval development test/*Haemonchus contortus*	0.3243 mg/mL (EC_50_)	Levamisole 1.88 mg/mL (EC_50_)	[46]
	Anthelmintic	Fecal egg count reduction test/*Haemonchus contortus*	0.1731 mg/mL (EC_50_)	Levamisole 1.88 mg/mL (EC_50_)	[46]
2,2′:5′,2′′-Terthiophene (α-Terthienyl) (**75**)	Cytotoxicity	MTT/SKOV3	77.23 µM (IC_50_)	Cisplatin 11.25 µM (IC_50_)	[39]
	Fungicidal	Broth microdilution/*C. albicans* ATCC 10231	0.24 µg/mL (MFC) in light	Amphotericin B 0.50 µg/mL (MFC) Fluconazole ˃ 64 µg/mL (MFC) Itraconazole ˃ 16 µg/mL (MFC)	[40]
	Fungicidal	Broth microdilution/*C. albicans* ATCC 10231	7.81 µg/mL (MFC) in low oxygen and light	-	[59]
	Fungicidal	Broth microdilution/*C. albicans* ATCC 10231	0.24 µg/mL (MFC) in normal oxygen and light	-	[59]
	Larvicidal	Larval mortality/*Aedes albopictus*	1.41 µg/mL (LC_50_)	Rotenone 3.75 µg/mL (LC_50_)	[41]
	Larvicidal		2.19 µg/mL (LC_95_)	Rotenone 9.45 µg/mL (LC_95_)	[41]
	Larvicidal	Larval mortality/*Anopheles sinensis*	1.79 µg/mL (LC_50_)	Rotenone 1.25 µg/mL (LC_50_)	[41]
	Larvicidal		2.54 µg/mL (LC_95_)	Rotenone 2.24 µg/mL (LC_95_)	[41]
	Larvicidal	Larval mortality/*Culex pipiens* pallens	1.38 µg/mL (LC_50_)	Rotenone 1.88 µg/mL (LC_50_)	[41]
	Larvicidal		2.15 µg/mL (LC_95_)	Rotenone 3.74 µg/mL (LC_95_)	[41]
	Nematicidal	Nematode Mortality/J2s of *Meloidogyne incognita*	0.56 µg/mL (LC_50_) in light	Abamectin 8.73 (LC_50_) in light	[19]
			1.77 µg/mL (LC_50_) in dark	Abamectin 9.38 (LC_50_) in dark	[19]
5-Formyl-2,2′:5′,2′′-terthiophene (Ecliptal) (**76**)	Cytotoxicity	MTT/SKOV3	24.57 µM (IC_50_)	Cisplatin 11.25 µM (IC_50_)	[39]
	Cytotoxicity	MTT/Hec1A	12.00 µM (IC_50_)	Cisplatin 120.42 µM (IC_50_)	[47]
	Cytotoxicity	MTT/Ishikawa	2.20 µM (IC_50_)	Cisplatin 10.11 µM (IC_50_)	[47]
	Anti-inflammatory	Colorimetric/5-LOX	26.18 µM (IC_50_)	Indomethacin 0.89 µM (IC_50_)	[43]
	Antibacterial	Broth microdilution/*S. aureus*	25.0 µM (MIC)	Penicillin G 0.156 µM (MIC)	[38]
5-Hydroxymethyl-2,2′:5′,2′′-terthiophene (α-Terthienylmethanol) *(77)*	Cytotoxicity	MTT/SKOV3	7.73 µM (IC_50_)	Cisplatin 11.25 µM (IC_50_)	[39]
	Cytotoxicity	MTT/Hec1A	0.38 µM (IC_50_)	Cisplatin 120.42 µM (IC_50_)	[47]
	Cytotoxicity	MTT/Ishikawa	0.35 µM (IC_50_)	Cisplatin 10.11 µM (IC_50_)	[47]
	Cytotoxicity	MTT/A2780	1.18 µM (IC_50_)	Cisplatin 10.80 µM (IC_50_)	[20]
	Cytotoxicity	MTT/SKOV3	15.51 µM (IC_50_)	Cisplatin 43.05 µM (IC_50_)	[20]
	Cytotoxicity	MTT/OVCAR3	0.20 µM (IC_50_)	Cisplatin 35.46 µM (IC_50_)	[20]
	Cytotoxicity	MTT/ES2	18.82 µM (IC_50_)	Cisplatin 29.58 µM (IC_50_)	[20]
	Antibacterial	Broth microdilution/*S. aureus*	25.0 µM (MIC)	Penicillin G 0.156 µM (MIC)	[38]
5-Hydroxymethyl-(2,2′:5′,2′′)-terthienyl angelate (**79**)	Cytotoxicity	MTT/Hec1A	129.85 µM (IC_50_)	Cisplatin 120.42 µM (IC_50_)	[47]
	Cytotoxicity	MTT/Ishikawa	6.87 µM (IC_50_)	Cisplatin 10.11 µM (IC_50_)	[47]
5-Hydroxymethyl-(2,2′:5′,2′′)-terthienyl tiglate (**80**)	Cytotoxicity	MTT/Hec1A	2.66 µM (IC_50_)	Cisplatin 120.42 µM (IC_50_)	[47]
	Cytotoxicity	MTT/Ishikawa	9.68 µM (IC_50_)	Cisplatin 10.11 µM (IC_50_)	[47]
5-Methoxy-(2,2′:5′,2′′)-terthiophene (**81**)	Cytotoxicity	MTT/Hec1A	1.38 µM (IC_50_)	Cisplatin 120.42 µM (IC_50_)	[47]
	Cytotoxicity	MTT/Ishikawa	7.12 µM (IC_50_)	Cisplatin 10.11 µM (IC_50_)	[47]
3′-Hydroxy-2,2′:5′,2′′-terthiophene-3′-O-β-D-glucopyranoside (**82**)	Cytotoxicity	*MTT*/SKOV3	58.20 µM (IC_50_)	Cisplatin 11.25 µM (IC_50_)	[39]
Thiotagetin A (**83**)	Cytotoxicity	MTT/KB	2.03 μg/mL (ED_50_)	Adriamycin 0.26 μg/mL (ED_50_)	[48]
	Cytotoxicity	MTT/MCF-7	3.88 μg/mL (ED_50_)	Adriamycin 0.07 μg/mL (ED_50_)	[48]
Rupestriene A (**86**)	In vitro anti-inflammatory/*NO*	LPS-stimulated production in BV-2 microglial cells	20.3 µM (IC_50_)	Quercetin 4.3 µM (IC_50_)	[36]
	Neuraminidase inhibitory activity	Fluorescence-based assay	351.15 µM (IC_50_)	Oseltamivir acid 77.91 µM (IC_50_)	[15]
7-[1-(Thiophene-5-yl)-1-formamido]-3-propylenyl-3-cephem-4-carboxylic acid (CAx1) (**87**)	Antibacterial	Broth microdilution/*S. aureus* MTCC 740	0.2 µg/mL (MIC) 2.0 µg/mL (MBC)	Penicillin 32.0 µg/mL (MIC) 64.0 µg/mL (MBC)	[40]
	Antibacterial	Broth microdilution/*B. subtilis* MTCC 736	0.25 µg/mL (MIC) 0.5 µg/mL (MBC)	Penicillin 0.5 µg/mL (MIC) 4.0 µg/mL (MBC)	[50]
	Antibacterial	Broth microdilution/*E. coli* MTCC 739	4.0 µg/mL (MIC) 8.0 µg/mL (MBC)	Penicillin 4.0 µg/mL (MIC) 16.0 µg/mL (MBC)	[50]
	Antibacterial	Broth microdilution/*K. pneumonia* MTCC 661	4.0 µg/mL (MIC) 16.0 µg/mL (MBC)	Penicillin 16.0 µg/mL (MIC) 64.0 µg/mL (MBC)	[50]
2,5-Bis(5-tert-butyl-2-benzoxazolyl)thiophene (**88**)	Antimicrobial	Broth microdilution/*E. faecalis* ATCC29212	256.0 µg/mL (MIC)	Streptomycin 256.0 μg/mL (MIC)	[51]
Thiocarboxylic A (**89**)	Antimicrobial	Broth microdilution/*E. coli* ATCC35218	1.7 µg/mL (MIC)	Streptomycin 2.3 µg/mL (MIC)	[16]
		Broth microdilution/*S. aureus* ATCC25923	1.7 µg/mL (MIC)	Streptomycin 0.1 µg/mL (MIC)	[16]
		Broth microdilution/*C. albicans* ATCC10231	3.3 µg/mL (MIC)	Amphotericin B 0.1 µg/mL (MIC)	[16]
Thiocarboxylic B (**90**)	Antimicrobial	Broth microdilution/*E. coli* ATCC35218	0.9 µg/mL (MIC)	Streptomycin 2.3 µg/mL (MIC)	[16]
		Broth microdilution/*S. aureus* ATCC25923	1.9 µg/mL (MIC)	Streptomycin 0.1 µg/mL (MIC)	[16]
		Broth microdilution/*C. albicans* ATCC10231	3.9 µg/mL (MIC)	Amphotericin B 0.1 µg/mL (MIC)	[16]
Thiocarboxylic C1 (**91**)	Antimicrobial	Broth microdilution/*E. coli* ATCC35218	7.0 µg/mL (MIC)	Streptomycin 2.3 µg/mL (MIC)	[16]
		Broth microdilution/*S. aureus* ATCC25923	3.5 µg/mL (MIC)	Streptomycin 0.1 µg/mL (MIC)	[16]
		Broth microdilution/*C. albicans* ATCC10231	7.0 µg/mL (MIC)	Amphotericin B 0.1 µg/mL (MIC)	[16]
Thiocarboxylic C2 (**92**)	Antimicrobial	Broth microdilution/*E. coli* ATCC35218	7.0 µg/mL (MIC)	Streptomycin 2.3 µg/mL (MIC)	[16]
		Broth microdilution/*S. aureus* ATCC25923	3.5 µg/mL (MIC)	Streptomycin 0.1 µg/mL (MIC)	[16]
		Broth microdilution/*C. albicans* ATCC10231	7.0 µg/mL (MIC)	Amphotericin B 0.1 µg/mL (MIC)	[16]
Thiocarboxylic D1 (**93**)	Antimicrobial	Broth microdilution/*E. coli* ATCC35218	3.5 µg/mL (MIC)	Streptomycin 2.3 µg/mL (MIC)	[16]
		Broth microdilution/*S. aureus* ATCC25923	3.5 µg/mL (MIC)	Streptomycin 0.1 µg/mL (MIC)	[16]
		Broth microdilution/*C. albicans* ATCC10231	7.0 µg/mL (MIC)	Amphotericin B 0.1 µg/mL (MIC)	[16]
Thiocarboxylic D2 (**94**)	Antimicrobial	Broth microdilution/*E. coli* (ATCC35218)	3.5 µg/mL (MIC)	Streptomycin 2.3 µg/mL (MIC)	[16]
		Broth microdilution/*S. aureus* ATCC25923	3.5 µg/mL (MIC)	Streptomycin 0.1 µg/mL (MIC)	[16]
		Broth microdilution/*C. albicans* ATCC10231	7.0 µg/mL (MIC)	Amphotericin B 0.1 µg/mL (MIC)	[16]
Rupestriene D (**95**)	Neuraminidase inhibitory activity	Fluorescence-based assay	986.54 µM (IC_50_)	Oseltamivir acid 77.91 µM (IC_50_)	[15]
Rupestriene E (**96**)	Neuraminidase inhibitory activity	Fluorescence-based assay	365.40 µM (IC_50_)	Oseltamivir acid 77.91 µM (IC_50_)	[15]

Compounds **43**, **46**, and **76**, separated from aerial parts of *Tagetes minuta*, significantly decreased NFκB p65, TNF-α, and IL-6 levels compared to indomethacin in an ELISA (enzyme-linked immunosorbent assay) [44]. In 2020, Ibrahim et al. reported that **43** and **76**, isolated from *T. minuta*, displayed moderate anti-inflammatory activity (IC_50_ 41.82 and 26.18 µM, respectively) in a 5-LOX colorimetric assay in comparison with indomethacin (IC_50_ 0.89 µM) [43].

### 4.2. Cytotoxic Activity

Cancer is a crucial cause of death globally, accounting for ≈10 million deaths in 2020 [48,60]. There are many available medications for treating various types of cancer. However, none of them are entirely safe and effective. Many of the reported thiophenes have been assessed for cytotoxic effectiveness against various cancer cell lines.

Four new derivatives, foetithiophenes C-F (**3**–**6**), along with foetithiophenes A (**1**) and B (**2**), were obtained from the MeOH extract of *Ferula foetida* roots using SiO_2_ CC and RP-HPLC. Unfortunately, they showed no cytotoxic activity (IC_50_ ˃ 100 µmM) against K562 and MCF-7 cell lines in an Alamar Blue assay [25].

Additionally, **9** exhibited more promising cytotoxic activity (IC_50_ 21.09 µM) than doxorubicin (IC_50_ 195.12 µM) against CEM/ADR5000 (human T-cell lymphoblast-like cell line). However, it was weakly active against CCRF-CEM (human leukemic cell line, IC_50_ 46.96 µM) in the resazurin reduction cytotoxic assay [30].

Compounds **11**, **18**, and **22**, isolated from *Pluchea indica* aerial parts, were assayed for inhibitory activity on coumarin 7-hydroxylation induced by CYP2A6 (cytochrome P450 2A6) and CYP2A13 (cytochrome P450 2A13) enzymes, using an enzymatic reconstitution assay [31]. The human liver cytochrome P450 (CYP) 2A13 and 2A6 enzymes had a crucial function in nicotine metabolism and the activation of tobacco-specific nitrosamine carcinogens. Their prohibition could represent a strategy for smoking abstinence and decreasing risks of lung cancer and respiratory complaints. It was found that **18**, **11**, and **22** irreversibly prohibited CYP2A6- and CYP2A13-induced coumarin 7-hydroxylation (IC_50_ values 3.90 and 2.40 µM, respectively, for **18**; IC_50_ 6.43 and 6.18 µM, respectively for **11**; and IC_50_ 4.44 and 2.94 µM, respectively, for **22**). These metabolites could aid in smoking stoppage and lessened risks of lung cancer and respiratory illnesses [31].

Xu et al. reported that the treatment of SW620 (human colon cancer) cells with PYDDT (2-(pro-1-ynyl)-5-(5,6-dihydroxypenta-1,3-diynyl) thiophene) (**18**) led to the induction of mitochondrial-mediated apoptosis, characterized by clavage of PARP (poly ADP ribose polymerase) cleavage, activation of caspase-3 and 9, release of cytochrome c from mitochondria, potential loss of mitochondrial membrane, Bcl-2 (B-cell lymphoma 2) downregulation, and Bax mitochondrial translocation. A mechanism study revealed that PYDDT induced SW620 apoptosis through a JNK (c-Jun N-terminal kinase)/ROS (reactive oxygen species)-mediated mitochondrial pathway [33].

Ecliprostins A–C (**65**–**67**) new thiophene derivatives were separated from *Eclipta prostrata*. In contrast, ecliprostins A (**65**) and B (**66**) featured a bithiophenyl acetylenic skeleton, incorporating an isovalerate unit, whereas ecliprostin C (**67**) was a dimer of **65**. They exerted no noticeable cytotoxicity versus Hela and MDA-MB-231 cell lines (Conc. 30 µM) [18].

Compounds **33**, **75**–**78**, and **82** were purified from the EtOH extract of *Eclipta prostrata* aerial parts using SiO_2_ CC (silica gel column chromatography) and purified using a reversed-phase CC. In the MTT assay, ***77*** exhibited the most potent cytotoxicity on SKOV3 cells (IC_50_ 7.73 µM), outperforming cisplatin (IC_50_ 11.25 µM). The terthiopenes **75**, **76**, and **82** showed significant cytotoxicity (IC_50_ values ranging from 24.57 to 77.23 µM). However, **33** and **78** were ineffective (IC_50_ values ˃ 100 µM) [39].

Additionally, Preya et al. reported that ***77*** isolated *Eclipta prostrata* was a more potent cell growth inhibitor (IC_50_s 0.20–18.82 µM) than cisplatin (IC_50_ 10.80 to 43.05 µM) against a panel of human ovarian cancer cell lines; OVCAR3, SKOV3, A2780, and ES2 in the MTT (3-(4,5-dimethylthiazol-2-yl)-2,5-diphenyl tetrazolium bromide) assay. It caused changes in S phase-linked proteins (cyclins A and D2 and cyclin-dependent kinase 2) and induced an intracellular increase in ROS that increased the levels of p-H2AX (H2A histone family member X), resulting in DNA (deoxyribonucleic acid) damage [14,20]. A mechanism study indicated that **77** caused S-phase cell cycle arrest by inducing ROS stress and DNA damage. Therefore, **77** could be a potential therapeutic lead for treating ovarian cancer.

Sibiricumthionol (**84**) and (+)-xanthienopyran (**85**) were purified from *Xanthium sibiricum* fruits extract using SiO_2_, RP-18 (reversed phase-18), and HPLC (high-performance liquid chromatography) that were characterized by spectroscopic, X-ray, and ECCD analyses, as well as ECD calculations. These metabolites were inactive (IC_50_ ˃ 10 µM) against HCT-116, BGC-823, HepG2, NCI-H1650, and A2780 cell lines in the MTT assay [49].

Compounds **76**, **77**, and **79**–**81** isolated from *Eclipta prostrate* showed prominent cytotoxic effectiveness against Hec1A (IC_50_ ranging from 0.38 to 129.85 µM) and Ishikawa (IC_50_ ranging from 0.35 to 9.68 µM) cells compared to cisplatin (IC_50_ 120.4 and 10.11 µM, respectively). Notably, **77** had a potent effect on Ishikawa and Hec1A cells (IC_50_ 0.35 and 0.38 µM, respectively) [37,47]. The inhibitory effect of **77** was mediated by the induction of apoptosis, triggering caspase activation and cytochrome c release into the cytosol. Additionally, it increased the ROS intracellular level and decreased GSH (glutathione). Therefore, its apoptotic effect was attributed to the generation of reactive oxygen species via NADPH (nicotinamide adenine dinucleotide phosphate) oxidase in human endometrial cancer cells [47].

Thiotagetin A (**83**) purified from *Tagetes minuta* possessed cytotoxic activity against MCF-7 and KB (ED_50_s 3.88 and 2.03 μg/mL, respectively), compared to adriamycin (0.07 and 0.26 μg/mL, respectively) in the MTT assay [48].

### 4.3. Antimicrobial Activity

Infectious diseases continue to be a serious worldwide health concern. Multidrug-resistant (MDR) pathogens significantly increased morbidity and mortality rates [61]. The continuous emergence of MDR pathogens drastically reduced the efficacy of the utilized antibiotics resulting in a growth rate of therapeutic failure [62]. Accordingly, new and effective antimicrobial agents to tackle microbial infections are needed [50].

Chitsazian-Yazdi et al. assayed the antimicrobial activity of **1**–**6** in broth microdilution method against *B. cereus* PTCC-1247, *C. albicans* ATCC-10231, and *E. coli* ATCC-8739. Whereas only **6** displayed the most potent activity (MIC 50 µg/mL) against *B*. *cereus,* compared to gentamicin (MIC 10 µg/mL) [25].

Mbaveng et al. purified **9** from the CH_2_Cl_2_ fraction of *Echinops giganteus* roots. It showed moderate and selective activities against *E. coli* ATCC-8739, *E. aerogenes* ATCC-13048 and -EA27, *K. pneumonia* ATCC11296, *P. stuartii* ATCC29916, *E. cloacae* BM47, and *P. aeruginosa* PA01 (MIC < 100 µg/mL) in the rapid INT (p-iodonitrotetrazolium) chloride assay [29].

In 2017, Postigo et al. reported the separation and structural elucidation of **37**, **43**, **44**, and **75** from the *n*-hexane extract of *Porophyllum obscurum* using preparative CTL (centrifugal thin layer) and TL (thin-layer) chromatography. These compounds were evaluated for their fungicidal activity against *C. albicans* ATCC-10231 and 25 clinical strains of *Candida* spp. isolates, the causative agents of oropharyngeal candidiasis, using broth microdilution. They exhibited fungicidal effectiveness with minimum fungicidal concentrations (MFC) ranging from 0.24 to 7.81 μg/mL under UV-A irradiation, whereas **32** with (MFC 0.24 μg/mL) and **43** with (MFC 3.90 μg/mL) were the most active metabolites [40]. In 2019, Postigo et al. evaluated their photoinactivation against *C. albicans* in parallel under darkness and light conditions. The results revealed that these thiophenes exhibited the highest activity under normal-light/oxygen atmosphere (MFCs ranged from 0.24 to 7.81 μg/mL). However, their effects decreased >200 times (MFCs ranged from 7.81 to 250 μg/mL) under low-oxygen conditions. On the other hand, all tested thiophenes had no antifungal activity in the dark under both oxygen conditions (MFC > 250 μg/mL). It was found that **75** was the most active photosensitizer and was the only one that generated a single oxygen at the MFC. Furthermore, it did not elevate sensitivities to oxidative and osmotic stressors and did not produce leakage or apoptosis [59]. Therefore, their antifungal mechanism was proposed to be photodynamic, considering that the absence of oxygen had a passive effect on the antifungal photosensitizing activity. Therefore, these features could encourage further assessments to confirm their potential application as photosensitizers in photodynamic antimicrobial therapy against fungal infections [59].

**Figure 8:**
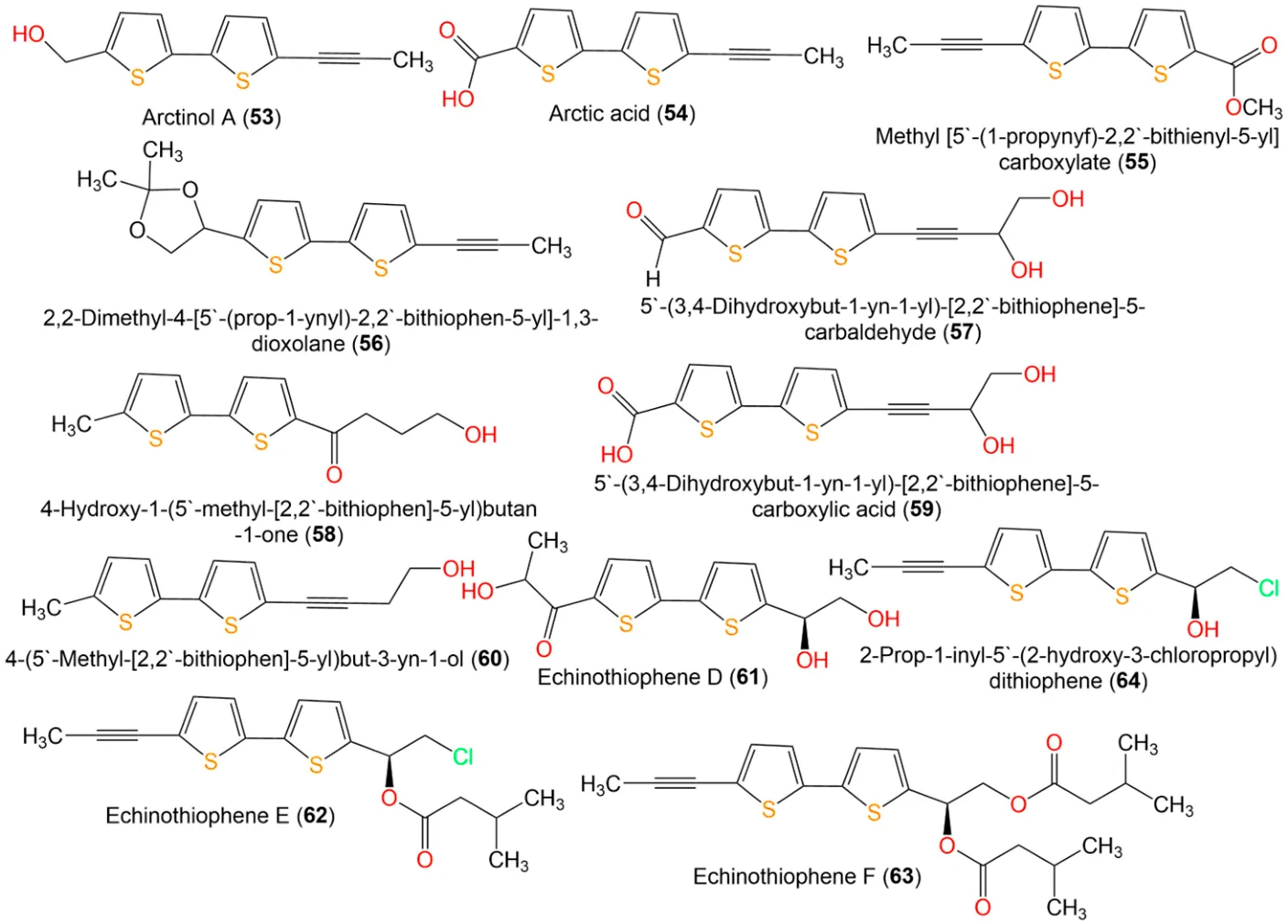
Structures of bithiophenes **53–63**.

Li et al. performed a broth microdilution assay to evaluate the antimicrobial activity of **7**, **9**, **33**, **34**, **43**, **45**, **47**, **49**, **52**–**54**, and **57**–**60** (Figure 8) isolated from *E. ritro* versus *E. coli*, *S. aureus*, and *C. albicans*. Compounds **43**, **49**, **53**, and **58** exhibited antibacterial activity against *S. aureus* comparable to that of levofloxacin (MIC (minimum inhibitory concentration) 8 µg/mL). Additionally, **43**, **49**, **52**, **53**, and **58** possessed activity against *E. coli* (MIC values of 32–64 µg/mL). On the other hand, **43**, **49**, and **58** displayed antifungal activity against *C. albicans* (MIC values of 32–64 µg/mL) that was similar or two-fold more active than that of levofloxacin (MIC 64 µg/mL) [27].

Liu et al. reported that **15**, **16**, **48**, **51**, **61**, **62**, and **64** possessed antifungal activity comparable to or greater than that of carbendazim against *Fusarium solani*, *Colletotrichum gloeosporioides*, *F*. *oxysporum* f. sp. *vasinfectum*, *Phytophthora infestans*, *Alternaria alternata*, and *F. oxysporum* f. sp. *niveum*, whereas **17**, **48**, **50**, **52**, and **63** exhibited weak antifungal activity (MICs from 32 to >256 µg/mL). It is noteworthy that **15** (MICs 4 and 8 µg/mL, respectively) had elevated inhibitory activity against *A*. *alternata* and *F. oxysporum* f. sp. *niveum* compared to **16**, **17**, and **62** (MICs from 8 to >256 µg/mL), indicating that acylation weakened the activity. Further, the effect of **15** and **16** versus all fungi was more than that of **17**, suggesting that chlorine could enhance activity [19].

Compounds **65**–**67** showed moderate growth inhibition against *S. aureus* (MICs 25.0, 6.25, and 25.0 µM, respectively) in the broth microdilution assay, compared to penicillin (MIC 0.156 µM) [18], whilst they did not have significant activity against *Vibrio vulnificus* and *E. coli* [18].

**Figure 9:**
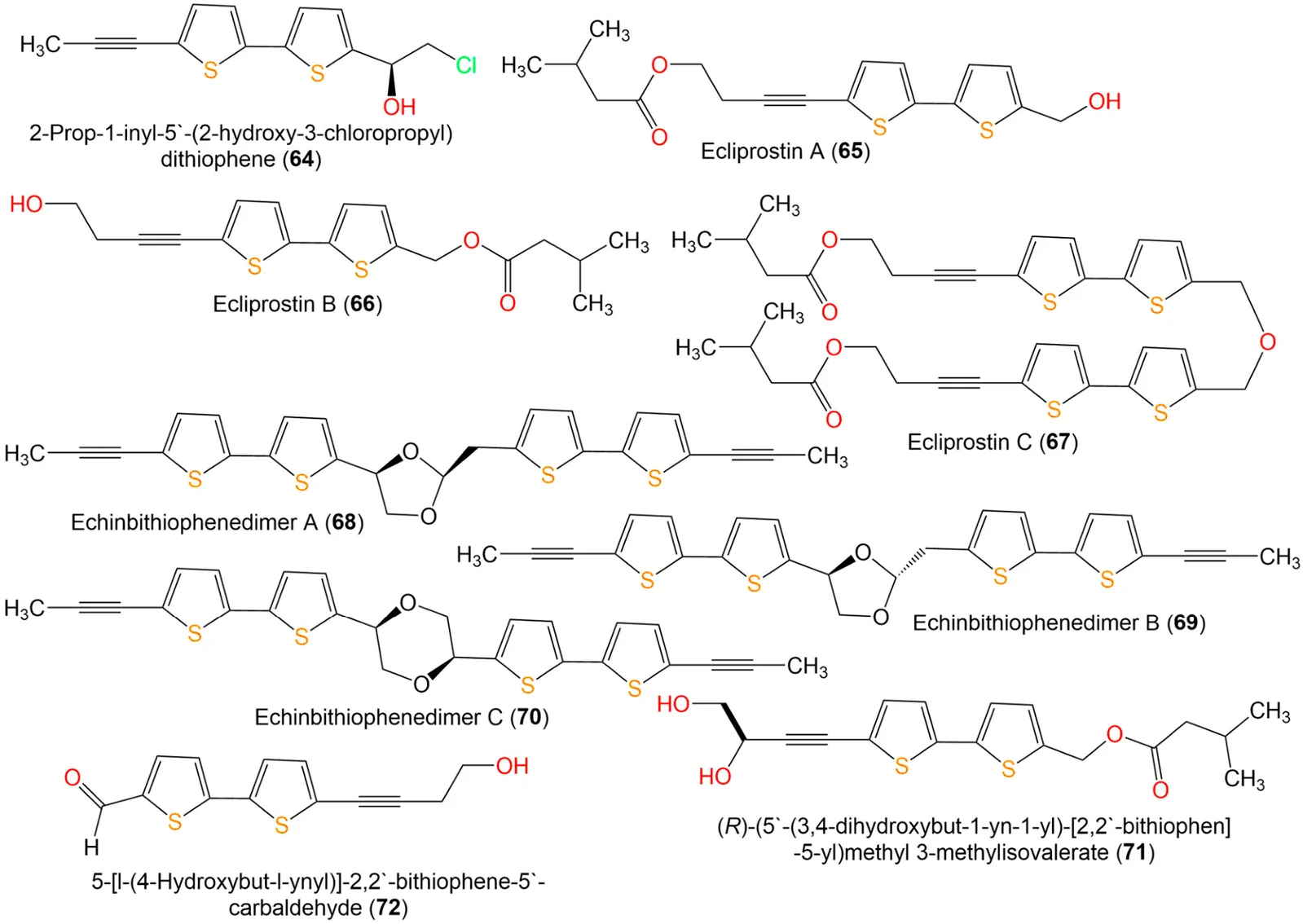
Structures of bithiophenes **64–72**.

Echinbithiophenedimers A–C (**68**–**70**) novel dimeric bithiophenes, besides **37** and **49**, were separated from *Echinops latifolius* using SiO_2_, Sephadex CC, and PTLC (Figure 9). Their antifungal activity against soil-borne fungi; *Pyricularia oryzae*, *Alternaria alternata*, *Colletotrichum gloeosporioides*, *Fusarium oxysporum*, and *Phytophthora infestans* were assessed in light and dark by the micro-broth dilution method. Compounds **68**–**70** had significant antifungal capacities against *P*. *oryzae* and *A. alternata* (MICs 8–16 µg/mL), whereas **70** (MIC 8 µg/mL) displayed better antifungal activity against *A. alternata* than carbendazim (MIC 16 µg/mL). Additionally, they revealed more antifungal activity (MIC 28 µg/mL) against *P. infestans* than carbendazim (MIC 256 µg/mL). It was found that an increased number of thiophene rings enhanced the activity [17].

**Figure 10:**
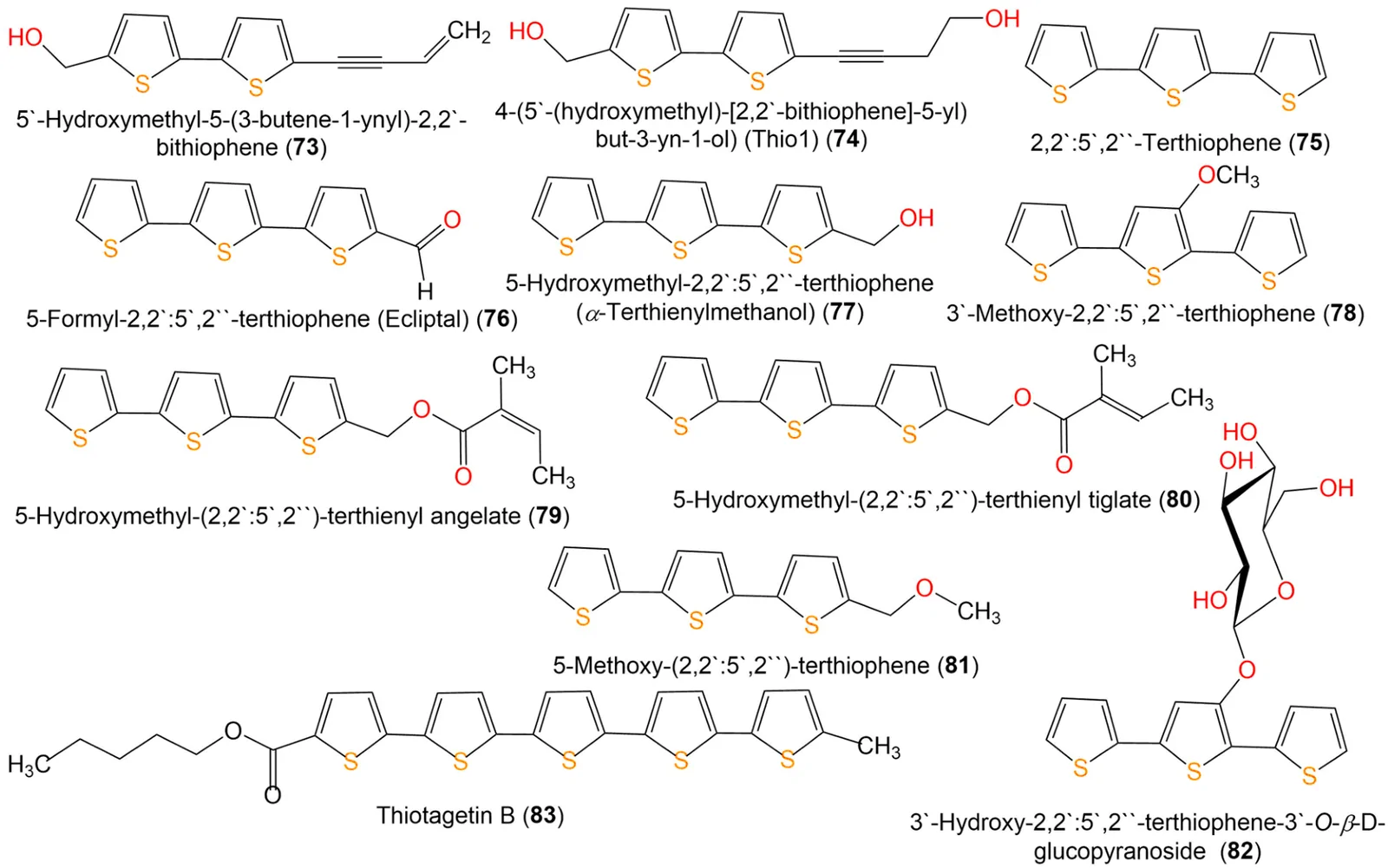
Structures of bithiophenes (**73** and **74**), terthiophenes (**75**–**82**), and quinquethiophene **83**.

Yu et al. purified two new thiophenes derivatives, **31** and **71**, together with **9**, **33**, **48**, **49**, **71**–**73**, **77**, and **82** from *Eclipta prostrata* by SiO_2_, Sephadex CC, and RP-HPLC [38]. Only **77** and **82** exerted mild antibacterial activity against *S. aureus* (MIC 25 µM) in the broth microdilution method, compared to penicillin G (MIC 0.156 µM) (Figure 10) [38].

Compound **87** was biosynthesized using endolithic *Streptomyces* sp. AL51. This compound exhibited remarkable antibacterial activity against both Gram-positive and Gram-negative bacteria in the microplate broth-dilution method. It displayed higher activity than penicillin against Gram-positive *S. aureus*, *B. subtilis*, *E. coli*, and *Klebsiella pneumonia,* with MIC/MBC (minimum bactericidal concentration) values of 0.2/2.0, 0.25/0.5, 4.0/8.0, and 4.0/16.0 µg/mL, respectively, compared to penicillin (MIC/MBC 32.0/64.0, 0.5/4.0, 4.0/16.0, and 16.0/64.0 µg/mL, respectively) [50].

Cao et al. purified **88** from the culture broth of the marine-derived actinomycete *Streptomyces* sp. G278. Compound **88** selectively inhibited *Enterococcus faecalis*, exhibiting activity comparable to that of streptomycin (MIC 256 μg/mL) [51].

**Figure 11:**
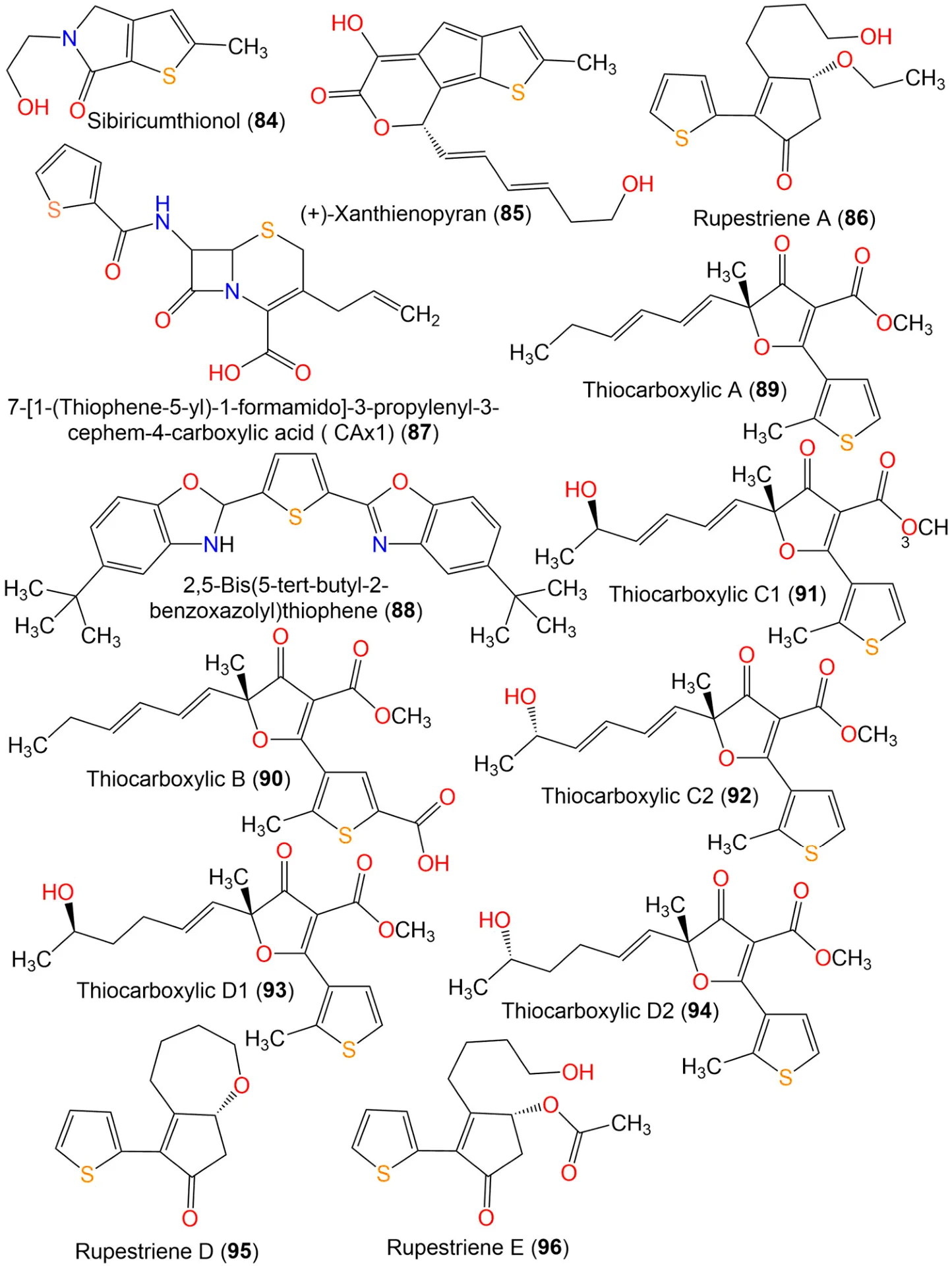
Structures of miscellaneous thiophenes **84–96**.

Six novel thiophene-furan-carboxylic acids, **89**–**94**, were isolated from the soil-derived fungus *Penicillium* sp. Sb62, representing the first class of natural furan-carboxylic acids containing a thiophene moiety (Figure 11). They exhibited antimicrobial activity against *E. coli*, *S. aureus*, and *C. albicans,* with MIC values of 0.9–7.0, 1.7–3.5, and 3.3–7.0 μg/mL, respectively, in the broth microdilution assay. It was observed that the absence of methoxy or hydroxy substituents on the side chain enhanced the activity, comparable to that of **89** and **90**. In contrast, the configuration of the methoxy or hydroxy groups on the side chain had a little effect on activity, as observed for compounds **91**, **92**, **93**, and **94** [16].

### 4.4. Antimalarial Activity

Malaria represents a significant parasitic disease worldwide, which is accountable for the death of at least half a million people yearly [63]. Globally, the estimated malaria cases in 2020 were 241 million in 85 malaria-endemic countries [64]. There is currently a significant increase in resistance to the available antimalarial drugs, necessitating the search for new drugs to combat malaria [65].

Bitew et al. evaluated the antimalarial activity of **9** and **14** isolated from the CH_2_Cl_2_ fraction of *Echinops hoehnelii* roots utilizing the standard suppressive test in *Plasmodium berghei*-affected mice. Compounds **9** and **14** at doses of 50 and 100 mg/kg decreased parasitemia levels by 43.2% and 50.2% and by 18.8% and 32.7%, respectively, compared to chloroquine. It was suggested that the ester functional group showed a two-fold decrease in the activity compared to **14** [32].

### 4.5. Larvicidal Activity

Currently used larvicides are synthetic pesticides with high toxic effects on humans and other non-targeted organisms. Several reports revealed that thiophenes demonstrated toxic effects on insects, especially larval mosquitoes. It has been proposed that thiophenes show promise as natural larvicides for mosquito control.

Zhao et al. reported that *E. grijsii* essential oil exhibited larvicidal activity against the fourth instar larvae of *Anopheles sinensis*, *Culex pipiens pallens*, and *Aedes albopictus* (LC_50_s (lethal concentrations 50%) values of 3.43, 1.47, and 2.65 µg/mL, respectively) in the larval mortality bioassay compared to rotenone. Further, the purified metabolites 5-BBT (5-(but-3-en-1-ynyl)-2,2′-bithiophene) (**37**), 5-IBT (5-(4-isovaleroyloxybut-1-ynyl)-2,2′-bithiophene) (**38**), and α-T (α-terthienyl) (**75**) possessed remarkable larvicidal effectiveness (LC_50_ values of 0.34, 0.45, and 1.41 µg/mL, respectively for *Ae. albopictus*, LC_50_ values of 1.36, 5.36, and 1.79 µg/mL, respectively for *An. sinensis*, and LC_50_ values of 0.12, 0.33, and 1.38 µg/mL, respectively for *C. pipiens pallens*) compared to rotenone (LC_50_ 3.75, 1.25, and 1.88 µg/mL, respectively) [41].

### 4.6. Nematicidal Activity

Nematodes and plant pathogenic fungi cause diseases that can lessen the yield and quality of several crops [66]. Chemical control utilizing synthetic-produced pesticides is a commonly used way to manage these diseases. The potential adverse effects of synthetic chemicals on non-target organisms and the development of pesticide resistance has driven the development of eco-friendly and safe pesticides [67]. Discovering efficient and less toxic natural pesticides has become a major focus in the modern pesticide industry [68].

Compounds **15**, **16**, **48**, **50**, **52**, **61**, **62**, and **64** showed more potent nematicidal effect against J2s (second-stage juveniles) of *Meloidogyne incognita* (LC_50_ values ranging from 0.42 to 8.28 μg/mL in light and from 0.86 to 9.23 μg/mL in dark) than abamectin (LC_50_ values 9.38 μg/mL in dark and 8.73 μg/mL in light). Noticeably, **61** and **64** exhibited better activity under dark conditions than under light conditions compared to control. Particularly, **64** was the most powerful metabolite against J2s (LC_50_ values of 0.91 and 0.86 μg/mL, under light and dark conditions, respectively) [13]. Compounds **48**, **49**, **51**, and **61**–**64** were regarded as non-phototoxic metabolites. It was found that the thiophene unit was fundamental for the activity. However, an increase in the number of acetylenes and chlorine enhanced the effect [13,19]. Compounds **68**–**70** were evaluated for their nematicidal activity against the J2s of *Meloidogyne incognita* under dark and light conditions in nematode mortality bioassays. They showed potent nematicidal activity (LC_50_ 9.39–18.17 µg/mL/dark and 8.73–16.53 µg/mL/light) compared to ethoprophos (LC_50_ values of 31.94 µg/mL/dark and 36.15 µg/mL/light). However, they had weaker nematicidal influences than α-terthienyl (phototoxic thiophene), suggesting that they were non-phototoxic. Furthermore, **70** exhibited more powerful activity (LC_50_ values of 8.73 and 9.39 µg/mL under light and dark, respectively) than its monomeric bithiophene **49**, revealing that the dimeric bithiophene framework with a 1,4-dioxane moiety in **70** enhanced the nematicidal activity [17].

Compound **74**, previously reported from the aerial parts of *Tagetes patula,* was synthesized by Politi et al. It exhibited a marked in vitro anthelmintic effect against *Haemonchus contortus*, exhibiting 100% efficacy in the larval development and egg hatch tests, with EC_50_ (effective concentration 50%) values of 0.3243 mg/mL and 0.1731 mg/mL, respectively, compared to levamisole (EC_50_ 1.88 mg/mL) [46].

### 4.7. Antioxidant and Anti-Influenza Activities

Compounds **43**, **46**, and **76** exhibited moderate antioxidant activity with % DPPH scavenging activity ranging from 41.87% to 45.17% at 100 µM [44].

Two new thiophene derivatives, rupestriene D (**95**) and rupestriene E (**96**), along with rupestriene A (**86**), were isolated from the whole plants of *Artemisia rupestris* using SiO_2_ CC and RP-HPLC. They exhibited neuraminidase inhibitory activity, with IC_50_ values ranging from 351.15 to 986.54 µM in the fluorescence-based assay compared to oseltamivir acid (IC_50_ 77.91 µM). Compounds **86** and **96** were more potent than **95**, indicating that a free OH group at the C-3 side chain might enhance the activity [15].

## 5. AI Target-Based Prediction vs. Virtual Screening and MD (Molecular Dynamics) for Thiophene Derivatives

Cathepsin D is one of the most abundant lysosomal proteases. It is implicated in protein turnover and favored apoptosis in proteostasis disruption [69,70]. The disturbance in its regulation can lead to various health disorders. Its excessive levels outside the cell membrane and lysosomes result in the growth of tumors, migration, invasion, and angiogenesis [71,72]. Many of the available inhibitors have non-specific inhibitory effects that may cause serious side effects [73]. Therefore, the currently tested thiophene derivatives as cathepsin D inhibitors could provide marked diagnostic benefits and a new therapeutic approach.

In order to detect the suitable protein targets for the thiophene derivatives, ligand-based tools were utilized for in silico target prediction [74]. In the current study, SuperPred, a prediction webserver, was used for the anatomical therapeutic chemical (ATC) code and target prediction of these compounds [75]. Based on the analysis of the results for all the predicted targets, cathepsin D with PDB (protein data bank) code 4OD9 was selected, as it is considered a common target for most of the thiophene derivatives with high probability and model accuracy percent (Table 3). All the listed compounds were docked, using extra precision for maximum accuracy; the docking method was validated by redocking the inhibitor N-(3,4-dimethoxybenzyl)-Nalpha-{N-[(3,4-dimethoxyphenyl)acetyl]carbamimidoyl}-D-phenylalaninamide (2*RZ*), which co-crystallized with 4OD9, and the RMSD values were within an acceptable range. All the redocked inhibitors revealed the same binding interaction with the active site as in the original pose. Further, the in silico ADMET properties of the investigated compounds were predicted. Eventually, MD simulations were conducted to assess the ligand/target interaction under simulated physiological circumstances for compound **30**, which showed high docking scores.

### 5.1. In Silico ADMET Properties of Selected Ligands

The reported 96 thiophene derivatives were processed using the LigPrep of the Schrodinger suite [76]. The OPLS3 force field generated 3D (three-dimensional) models with ionization states at 7.0 ± 0.2 pH. The QikProp module of the Schrodinger suite was utilized for predicting the ADME properties [77]. The predicted ADMET properties are summarized in Table 4. The ADMET analysis describes and determines the biological function, drug-likeness, physicochemical characters, and expected toxicity of the compounds. This is translated in terms of evaluating the usefulness of the molecules. The examined descriptors, such as drug likeness, solvent accessible surface area, dipole moment, molecular weight, hydrogen bond acceptor, and donor traits, aqueous solubility, octanol–water coefficient, number of likely metabolic reactions, brain/blood partition coefficient, human oral absorption, binding to human serum albumin, central nervous system activity, IC_50_ value for blockage of HERG K^+^ (human ether-a-go-go-related gene potassium) channels, and number of reactive functional groups were predicted for the reported thiophene derivatives. Most of the predicted values obtained for the compounds are in the recommended range, except for some highlighted parameters with yellow color.

### 5.2. Ligand and Protein Preparation

**Figure 12:**
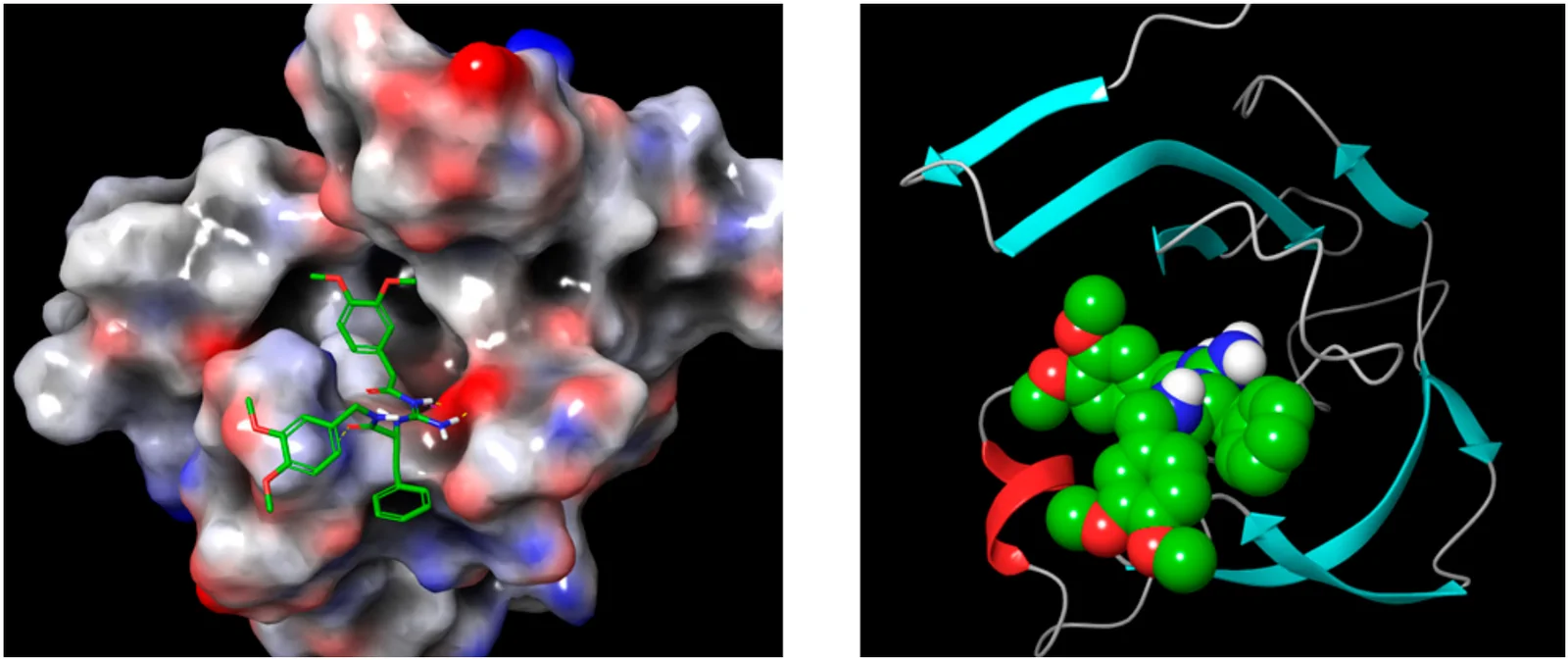
N-(3,4-dimethoxybenzyl)-Nalpha-{N-[(3,4-dimethoxyphenyl)acetyl] carbamimidoyl}-D-phenylalaninamide (2RZ) complexed with cathepsin D PDB: 4OD9 after preparation, using the Protein Preparation Wizard (Schrodinger), in which hydrogen bonds were optimized, and the whole complex was minimized and represented as a 3D structure.

Using LigPrep, 2D structures were converted to 3D, and tautomerization and ionization gave 146 minimized 3D structures that were utilized for docking with the cathepsin D crystal structure (PDB: 4OD9). The 4OD9 prepared by the protein preparation wizard tool minimized the geometry and optimized the H-bond network. Specifying the proper force field treatment and the formal charge was accomplished by the addition of correct ionization states and missing hydrogen atoms (Figure 12).

### 5.3. Molecular Docking Studies

After designating the grid box in the prepared protein through Glide’s Receptor-Grid Generation tool in Maestro [78], the obtained 3D molecular structures were docked into the binding site of the cathepsin D co-crystallized inhibitor. Table 5 shows the results of the docked ligands, which were selected based on their most negative docking scores. These scores demonstrated the relative binding affinities and conformations of the best-bonded ligands. Compounds **29** and **30** displayed the highest negative docking scores of −9.439 and −9.178 kcal/mol in complex with 4OD9, respectively, while the reference inhibitors (2RZ) showed a score of −6.895 kcal/mol in complex with the same protein.

**Figure 13:**
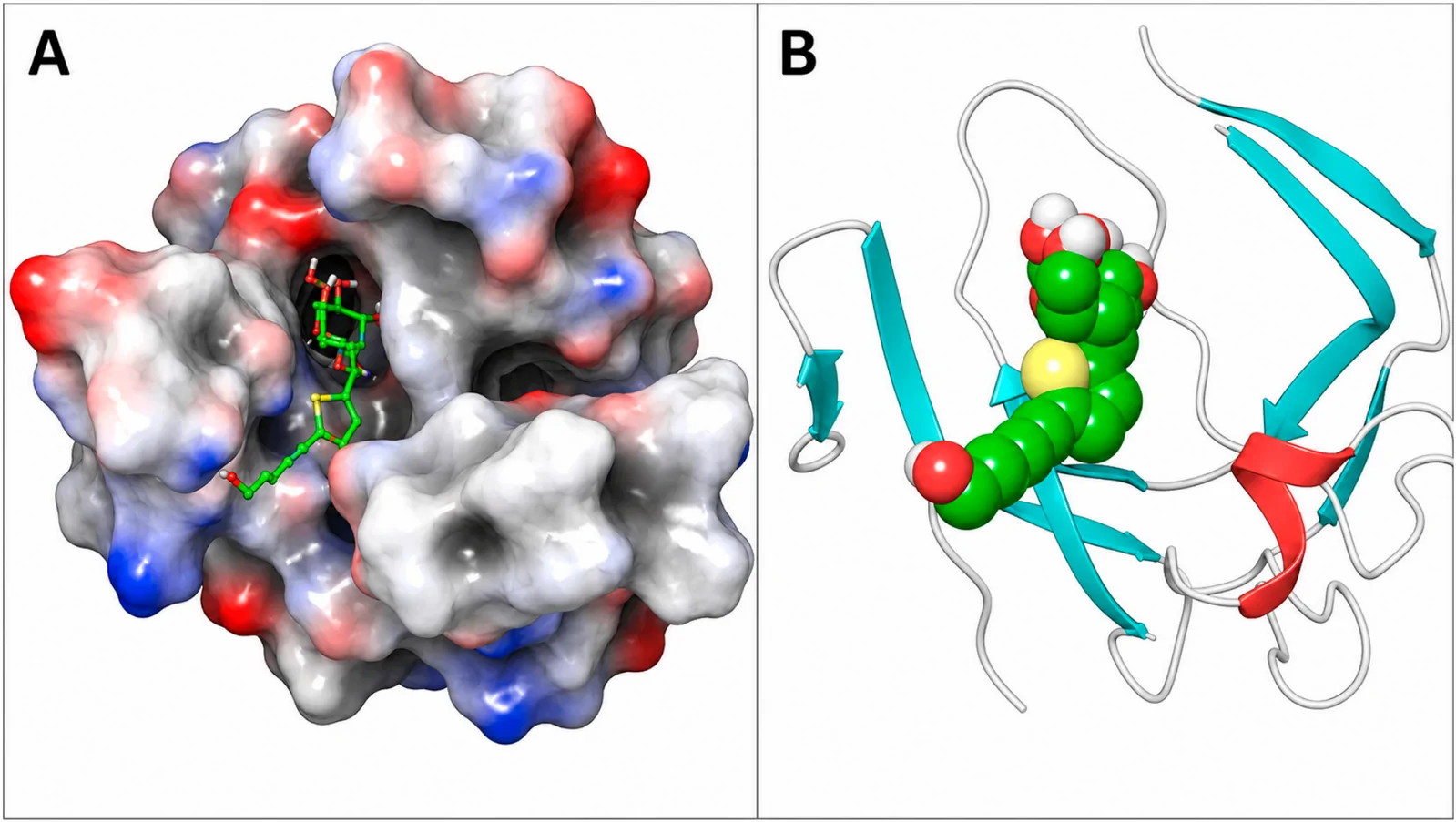
Cathepsin D in complex with compound **29**, represented as a 3D molecular surface and ribbon structure. In the molecular surface representation, red and blue regions indicate negatively and positively charged electrostatic surface regions, respectively, while white/grey regions indicate neutral surface areas. The ligand is shown in green, with heteroatoms colored by atom type. In the ribbon representation, the protein secondary-structure elements are shown as colored ribbons, and compound **29** is shown in space-filling representation.

**Figure 14:**
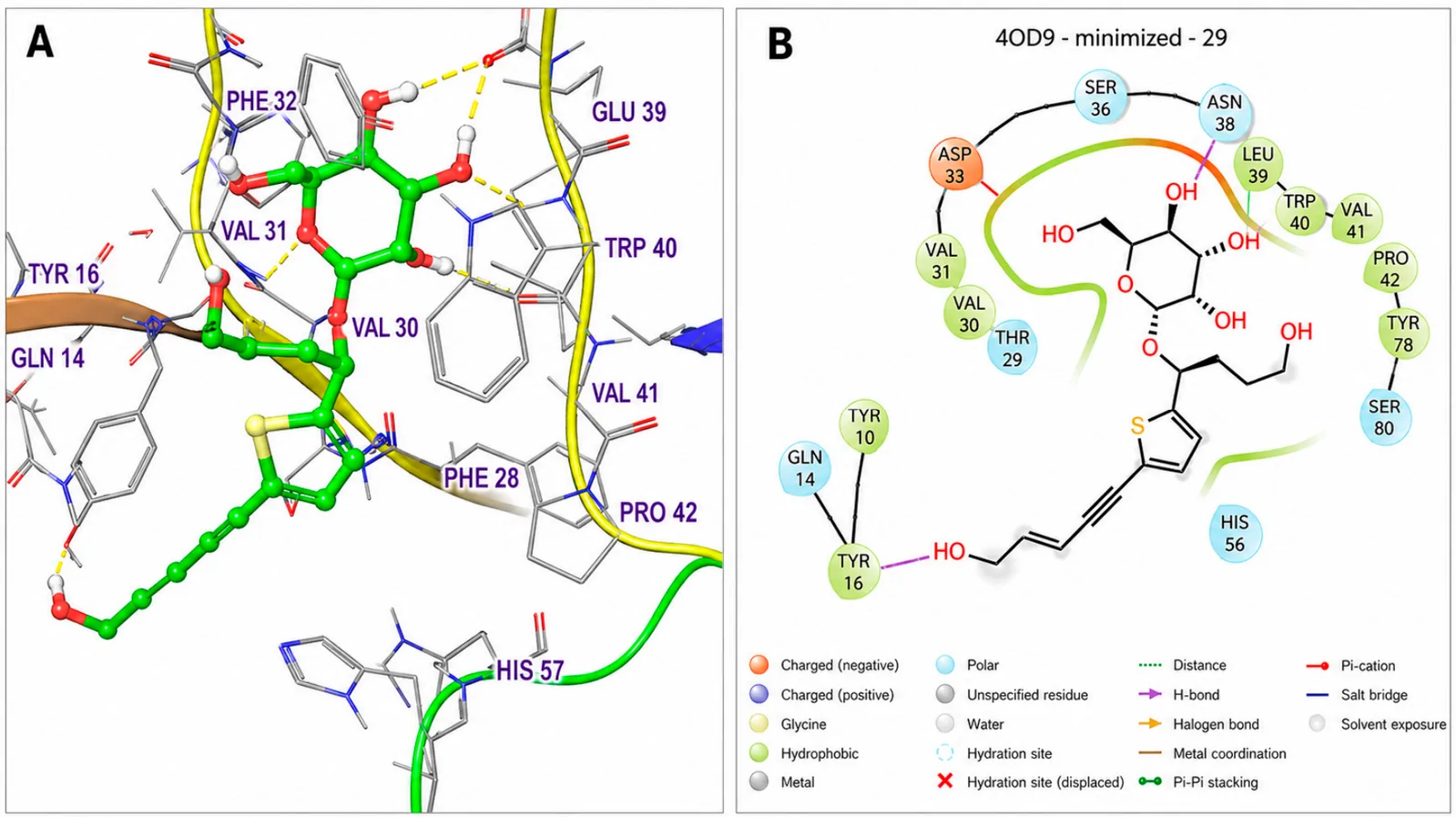
Putative binding mode of compound **29** in the binding site of cathepsin D (**PDB ID: 4OD9**). (**A**) Three-dimensional docking pose showing compound 29 as green sticks, the binding-site residues as grey sticks, and hydrogen bonds as yellow dotted lines. (**B**) Two-dimensional ligand–protein interaction diagram for compound **29**. Residue colors and interaction symbols are defined in the figure legend; only the residue classes and interaction types displayed in the diagram should be interpreted for this binding pose.

**Figure 15:**
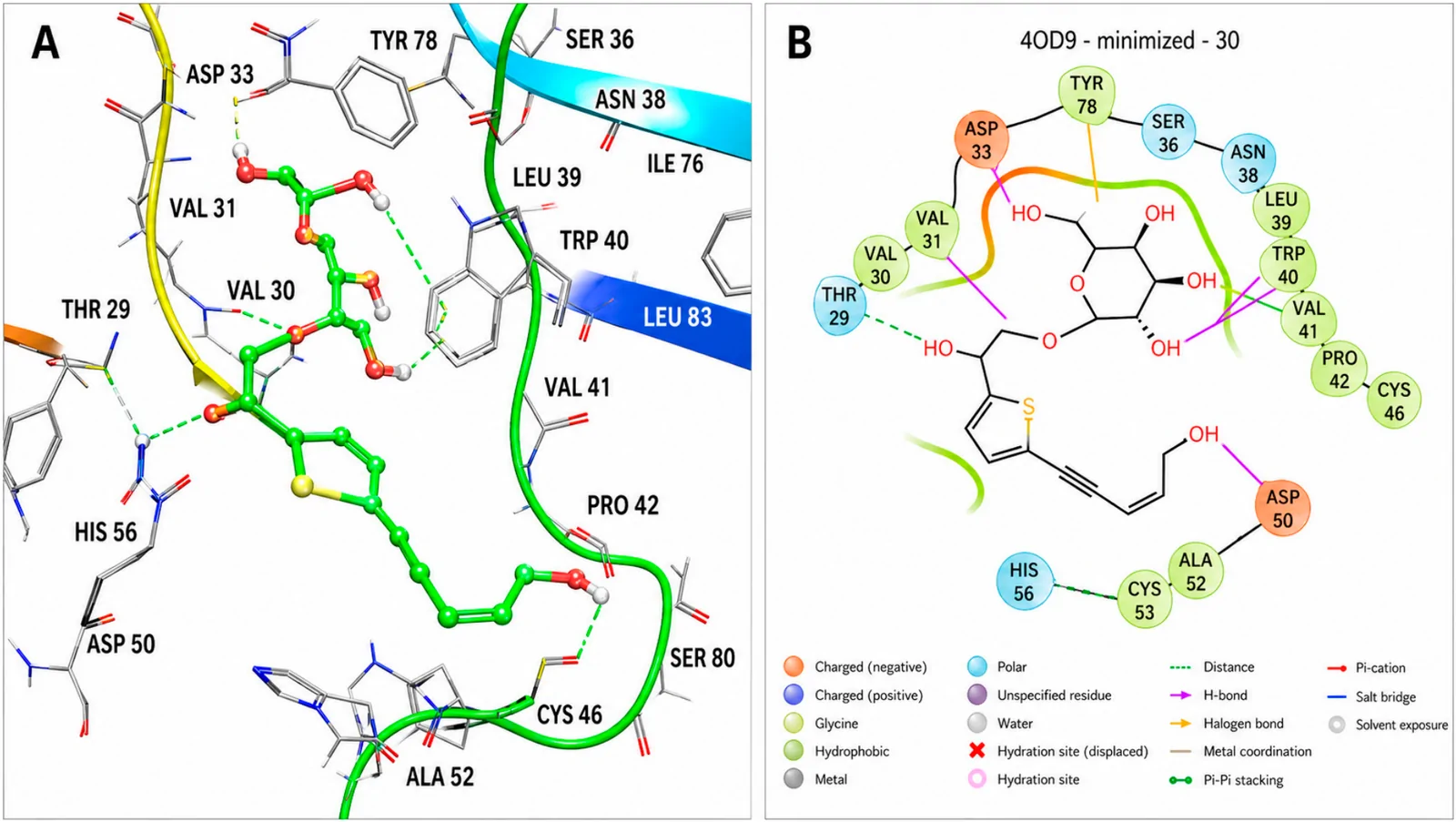
Putative binding mode of compound **30** in the binding site of cathepsin D (**PDB ID: 4OD9**). (**A**) Three-dimensional docking pose showing compound **30** as green sticks, the binding-site residues as grey sticks, and hydrogen bonds as yellow dotted lines. (**B**) Two-dimensional ligand–protein interaction diagram for compound 30. Residue colors and interaction symbols are defined in the figure legend; only the residue classes and interaction types displayed in the diagram should be interpreted for this binding pose.

**Figure 16:**
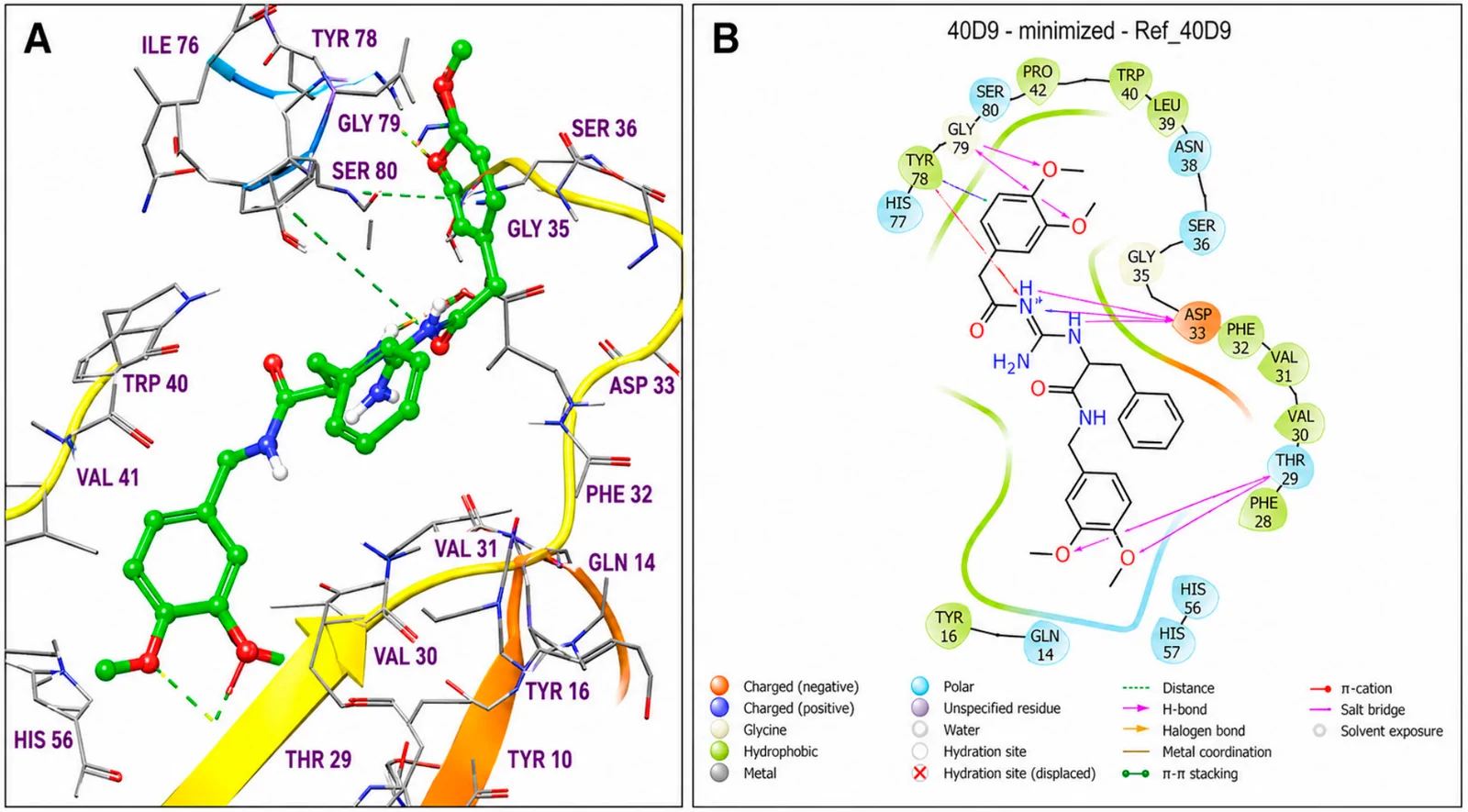
Putative binding mode of the reference inhibitor in the binding site of cathepsin D (**PDB ID: 4OD9**). (**A**) Three-dimensional docking pose showing the reference inhibitor as green sticks, the binding-site residues as grey sticks, and hydrogen bonds as yellow dotted lines. (**B**) Two-dimensional ligand–protein interaction diagram of the reference inhibitor. Residue colors and interaction symbols are defined in the figure legend; only the residue classes and interaction types displayed in the diagram should be interpreted for this binding pose.

Analysis of the docking of **29** and **30** compared with the redocked reference 2RZ indicated that they interacted through hydrogen bonds (Figure 13, Figure 14, Figure 15 and Figure 16) with the binding site residues of cathepsin D (4OD9). The binding site residues VAL 31, ASN 38, and TRP 40 of cathepsin D formed hydrogen bonds with the different hydroxyl groups of the sugar unit. TRY 16 interacted with the terminal hydroxy group of **29**. Whilst the binding site residues VAL 31, ASP 33, and TRP 40 of cathepsin D formed hydrogen bonds with the various OH groups of sugar unit, and ASP 50 interacted with the terminal hydroxy group of **30**.

### 5.4. Molecular Dynamics Simulation

**Figure 17:**
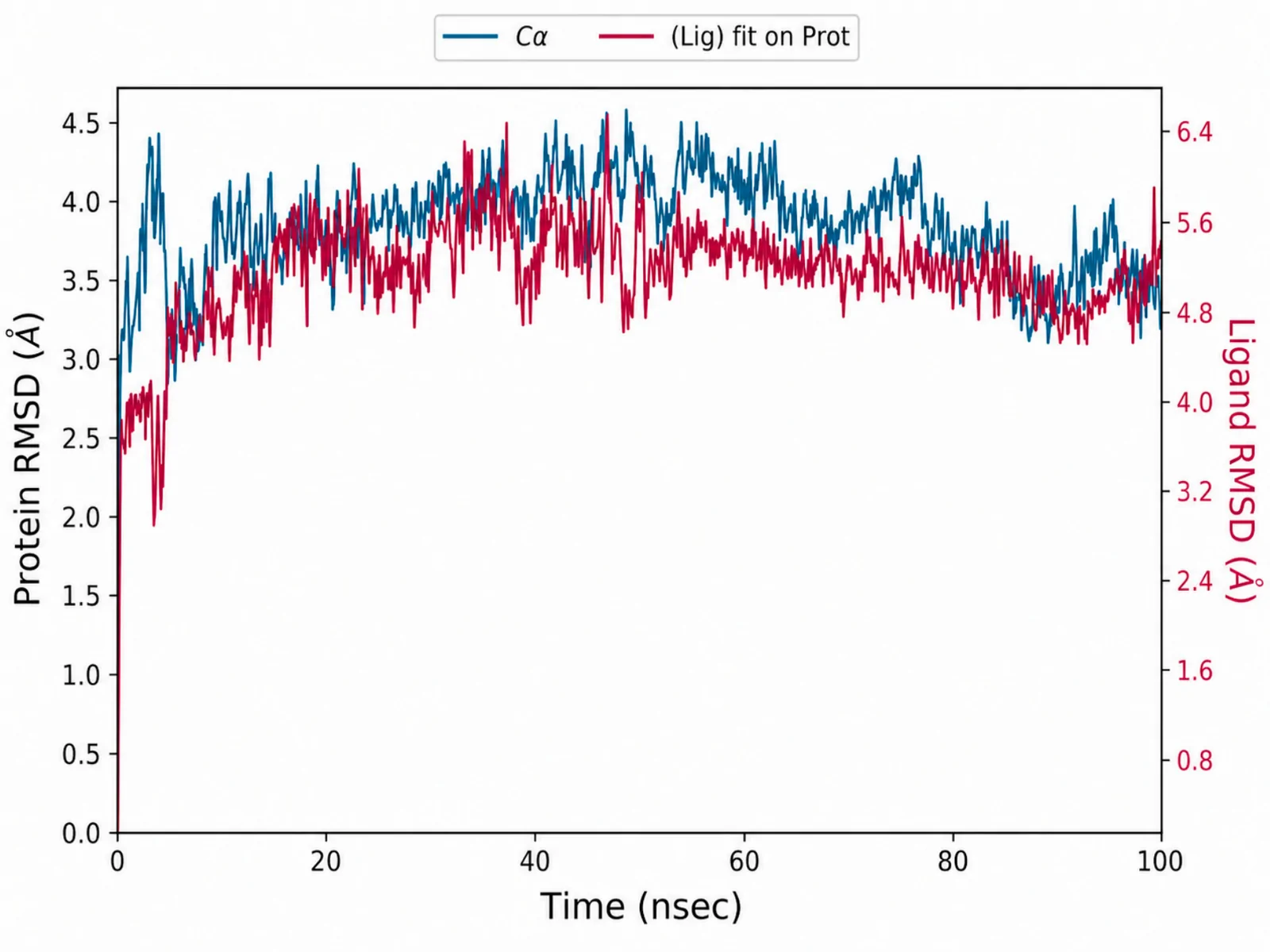
RMSD analysis of compound 30 complexed with cathepsin D (PDB Code) of the MD simulation trajectory. The RMSD plot was obtained for compound **30** complexed with cathepsin D (PDB ID 4OD9). The 100 ns simulation time reaffirms the stability of the complex without any significant changes in the structure.

**Figure 18:**
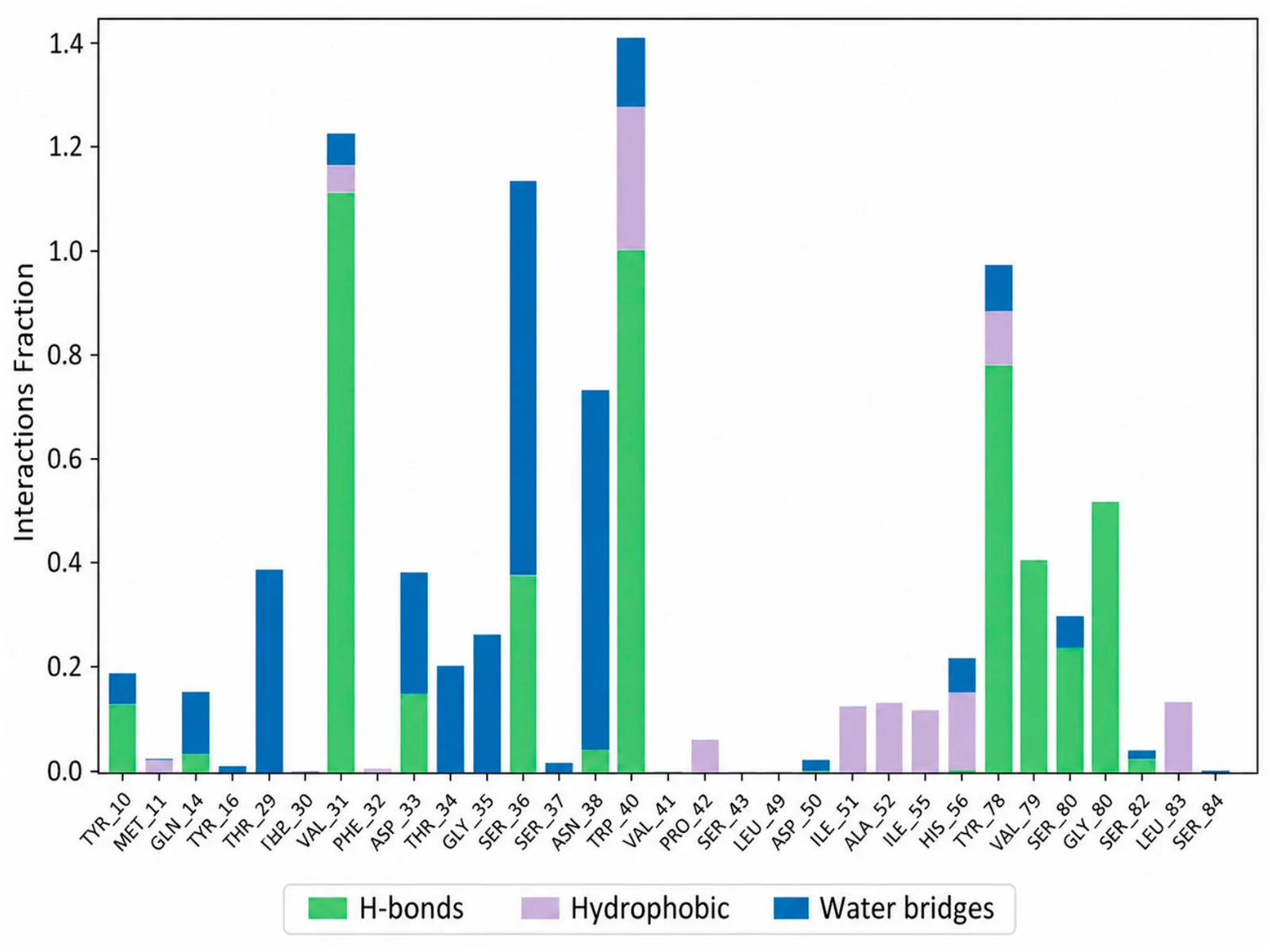
Cathepsin D interactions with compound **30** throughout the 100 ns molecular dynamics simulation. The stacked bars show the fraction of simulation time during which each residue interacted with the ligand. Interaction classes are color coded as follows: hydrogen bonds, hydrophobic contacts, ionic interactions, and water bridges, as indicated in the figure legend. The values are normalized over the trajectory; for example, a value of 0.7 indicates that the interaction was present for 70% of the simulation time. Values greater than 1.0 may occur when a residue forms multiple interactions of the same type with the ligand. If an interaction class is listed in the legend but no bar of that color is visible, no persistent interaction of that class was observed for the displayed residues.

The docking operation is a static view for the molecule’s binding in the active site of the specific protein. MD simulation computes the time versus atoms motions. By using Desmond software [79,80,81], the stability and frequency of compound **30** complex with cathepsin D with PDB codes 4OD9, MD simulations were performed for 100 ns. The complex structure was optimized at pH 7.0 ± 2.0. Complex stability was evaluated by analyzing the interaction map and the RMSD (root mean square deviation) plots of the ligand and protein. The RMSD plot in Figure 17 for the compound **30** complexed with cathepsin D indicated that the complexes remained relatively stable over 100 ns, with protein Cα RMSD fluctuating around ~2–3 Å and ligand heavy-atom RMSD mostly in the 2–4 Å range; hence, it can be regarded as non-significant. Since the RMSD plots of compound **30** and protein backbone overlapped, the stable complex formation can be inferred. Figure 18 shows the schematic of detailed ligand–atom interactions of compound **30** with cathepsin D. The docked poses were maintained through the simulation time of 100 ns, i.e., molecular interactions with residues VAL 31, SER 36, ASN 38, TRP 40, and TYR 78.

**Figure 19:**
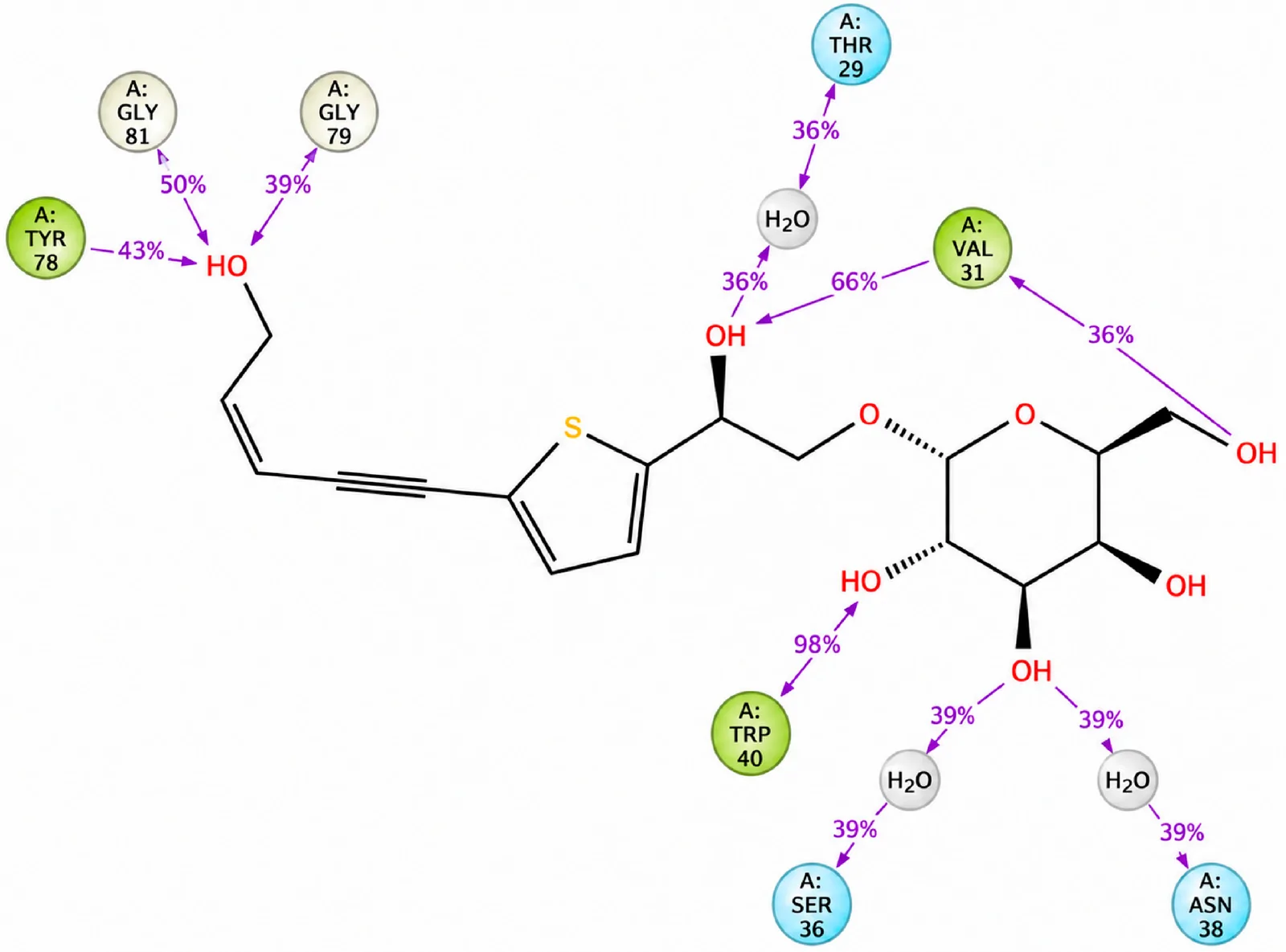
Two-dimensional schematic diagram showing the detailed atomic interactions of compound **30** with cathepsin D during the molecular dynamics simulation. Arrows indicate ligand–protein or water-mediated interactions, and the percentages beside the arrows indicate the fraction of the 100 ns trajectory during which each interaction was maintained. Only interactions occurring for more than 30.0% of the simulation time are displayed. Values above 100% can occur when the same residue forms multiple interactions of the same type with the ligand atom during the trajectory.

Figure 19 represents the ligand–protein interactions, which are characterized into four types: ionic, hydrophobic, hydrogen bonds, and water bridges. Each interaction type includes more specified subtypes, which can be investigated via the ‘Simulation Interactions Diagram’ panel [82,83,84,85,86]. The stacked bar charts were normalized throughout the trajectory: for example, a value of 0.7 suggests that 70% of the simulation time, the specific interaction, is maintained. Values over 1.0 are possible, as some protein residue may make multiple contacts of the same subtype with the ligand. Hydrogen bonding with residues VAL 31, TRP 40, and TYR 78 was retained for more than 80% of the simulation time.

### 5.5. Materials and Methods

#### 5.5.1. Preparation of PDB Structures

The PDB structure (PDB IDs: 4OD9) was downloaded from the Protein Data Bank [69], prepared and optimized utilizing the “Protein preparation wizard” [70] tool of Schrödinger suite [76,87]. For this reason, the bond orders for known HET groups and untemplated residues were identified, and hydrogens were added. Then, bonds to metals were broken, zero-order bonds were assigned among metals and nearby atoms, and formal charges were corrected for metals and neighboring atoms. From HET groups, water molecules beyond 5 Å were deleted. Disulfide bonds were generated. For ligands, metal HET states and cofactors were generated at 7.0 ± 2.0 pH using LigPrep [76]. Finally, H-bonds were optimized at pH 7.0 using PROPKA [88], the water molecules beyond 3 Å were removed from the HET groups, and restrained minimization was performed using the OPLS4 force field.

#### 5.5.2. ADME Properties Prediction

The drug likeness and ADME properties of the chosen compounds were estimated via the Maestro Schrodinger QikProp module in terms of metabolism, distribution, excretion, absorption, etc. [77].

#### 5.5.3. Receptor Grid Generation and Docking

Glide [78] was utilized for both grid generation and ligand docking. For docking of the 96 thiophene derivatives, a grid was generated using PDB: 4OD9, with the binding region specified by selecting 2RZ. The non-polar atoms were set for the VdW radii scaling factor by 1.0, and the partial charge cut-off of 0.25 was applied. Ligand docking was performed using the “ligand docking” tool of the Schrödinger suite [78,85]. The selected protocol was standard precision (SP), the ligand sampling method was flexible, and all the other settings were default.

#### 5.5.4. MD Simulations of Compound 30 in Complex with 4OD9

MD simulations were run using the Schrödinger suite [88]. The system of compound **30** in complex with 4OD9 was retrieved from the docking results and first tuned through the “System Builder” tool. The TIP3P solvent model and then an orthorhombic box was chosen. The system was neutralized by adding Na^+^ ions, and the box size was set to 10 Å. The MD calculations were run for 100 ns per trajectory, and the number of atoms, pressure, and temperature were kept constant (NPT ensemble). In contrast, the pressure was set to 1.01325 bar and the temperature to 300.0 K, and the OPLS4 force field was used.

## 6. Conclusions

Natural products are characterized by enormous scaffold diversity and structural complexity, which contribute to drug discovery. Sulfur-containing natural metabolites are a large class of significant functional molecules with potent biological activities and pharmacological properties; some of them have been developed into essential drugs. In the current work, 96 naturally occurring thiophenes have been reported from 2015 till now. Most of them had one to three thiophene rings. However, dimeric bithiophenes with four thiophene rings and quinquethiophenes with five thiophene rings were extremely rare. These metabolites have been mainly evaluated for their cytotoxic, antimicrobial, anti-inflammatory, and nematicidal activities. On the other hand, there are limited reports on their antimalarial, larvicidal, antioxidant, and anti-influenza activities. Some of them showed remarkable cytotoxic effects. Therefore, they could be a potential therapeutic agent for treating various cancers. However, in vivo studies and their detailed mechanism of action need to be determined.

**Figure 20:**
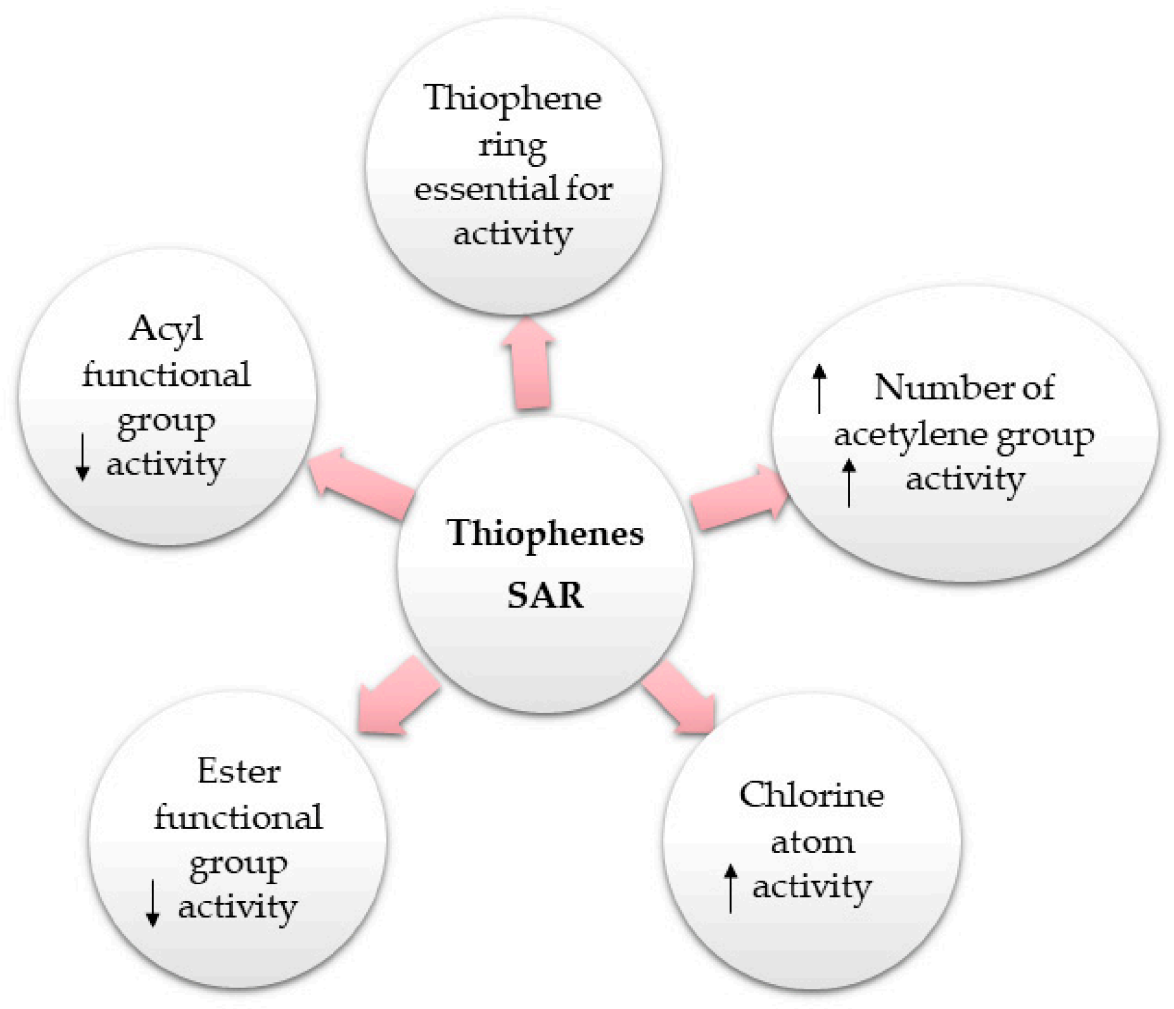
Summary of the reported structure–activity relationship (SAR) of thiophenes. ↑: increase; ↓: decrease [19,32,34,41].

Furthermore, they showed marked antifungal activity against various plant pathogenic fungi and had a remarkable nematicidal effect. Hence, they could provide worthy insights for discovering effective, eco-friendly nematicides and fungicides. More studies on formulation development are needed to upgrade stability and efficacy and cut down costs. Additionally, field assessment and research on the effects of these compounds on non-target organisms are compulsory. Their antifungal mechanism was proposed to be photodynamic. However, further assessments are needed to confirm their potential for application as photosensitizers in photodynamic antimicrobial chemotherapy against fungal infections. The structure–activity relationship study revealed that the presence of the acetylene group in the side chain increased the larvicidal and antimalarial activity. However, the attachment of the acetylenic side chain to an ester and acyl functional groups lowered the activity [19,32,34,41]. Additionally, increasing the number of acetylene groups in the side chain increased the anti-inflammatory, nematocidal, and antifungal activities [19,32,34,41]. Further, the attachment of this chain with the chlorine group enhanced the activity [19] (Figure 20). Estimation of other potential bioactivities and derivatization of these metabolites, along with mechanistic and in vivo studies, should be the target of future research. Precursor-based combinatorial biosynthesis (PCB) could be used for enhancing the production and structural modification of these metabolites. In this technique, a precursor analog has to be fed to the producing microorganism, resulting in the production of novel thiophenes with a potential pharmaceutical significance that is effective and ecologically friendly [89].

Based on the in silico, including ADMET properties prediction, molecular docking for the protein–ligands binding interaction, and molecular dynamics, these metabolites were identified as potential inhibitors for the cathepsin D and will serve as potential leads for the treatment of several diseases that are affected by the dysregulation of this enzyme. The results of the studies described in this review are undoubtedly significant. They represent an initial step in the search for new drug candidates and in evaluating the strength of the tested metabolites concerning the currently used drugs. Furthermore, this review provides an overview of the research progress on naturally isolated thiophenes, with particular emphasis on their bioactivities, which could attract the attention of many natural product researchers and encourage further investigation of their mechanism, efficacy, and safety through in vivo studies. The structural diversity of thiophenes could be of considerable synthetic interest as novel chemical entities for drug discovery. Furthermore, this work may encourage further research on the isolation, characterization, and bio-evaluation of these thiophenes, which may provide more candidates for the pharmaceutical industry. Additionally, it aims to provide a reference for researchers to facilitate the rapid identification of isolated thiophenes through comparison of their physical and spectral data. Future investigations, including combinatorial chemistry and drug design, will inevitably reveal new avenues for the advancement of drug discovery.

## Figures and Tables

**Table 1:** Naturally occurring mono-, bi-, ter-, and quinquethiophenes and miscellaneous derivatives (name, source, molecular weights and formulae, and location).

Compound Name	Source	Extract/Fraction	Mol. Wt.	Mol. Formula	Location	Ref.
I. Monothiophenes						
Foetithiophene A (**1**)	*Ferula foetida*, roots (Apiaceae)	MeOH/EtOAc-soluble fraction	170	C_8_H_10_O_2_S	Dorouneh village mountains, Khorasan Razavi, Iran	[25]
Foetithiophene B (**2**)	*Ferula foetida*, roots (Apiaceae)	MeOH/EtOAc-soluble fraction	218	C_9_H_14_O_2_S_2_	Dorouneh village mountains, Khorasan Razavi, Iran	[25]
Foetithiophene C (**3**)	*Ferula foetida*, roots (Apiaceae)	MeOH/EtOAc-soluble fraction	186	C_9_H_14_S_2_	Dorouneh village mountains, Khorasan Razavi, Iran	[25]
Foetithiophene D (**4**)	*Ferula foetida*, roots (Apiaceae)	MeOH/EtOAc-soluble fraction	310	C_16_H_22_S_3_	Dorouneh village mountains, Khorasan Razavi, Iran	[25]
Foetithiophene E (**5**)	*Ferula foetida*, roots (Apiaceae)	MeOH/EtOAc-soluble fraction	202	C_9_H_14_OS_2_	Dorouneh village mountains, Khorasan Razavi, Iran	[25]
Foetithiophene F (**6**)	*Ferula foetida*, roots (Apiaceae)	MeOH/EtOAc-soluble fraction	188	C_9_H_16_S_2_	Dorouneh village mountains, Khorasan Razavi, Iran	[25]
5-Propinyl-thiophene-2-Carboxylic acid = Junipic acid (**7**)	*Artemisia sieversiana*, aerial parts (Asteraceae)	EtOH/CH_3_Cl-soluble fraction	166	C_8_H_6_O_2_S	Qinghai, China	[26]
	*Echinops ritro*, whole plant (Asteraceae)	EtOH/EtOAc-soluble fraction	-	-	Qinghe County, Xinjiang, China	[27]
3-Hydroxy-5-propinyl-2-acetyl-thiophene (**8**)	*Artemisia sieversiana*, aerial parts (Asteraceae)	EtOH/CH_3_Cl-soluble fraction	180	C_9_H_8_O_2_S	Qinghai, China	[26]
2-(3,4-Dihydroxybut-1-ynyl)-5-(penta-1,3-diynyl)thiophene = 2-(Penta-1,3-diynyl)-5-(3,4-dihydroxybut-1-ynyl)-thiophene = 4-(5-(Penta-1,3-diyn-1-yl)thiophen-2-yl)but-3-yne-1,2-diol = 2-(Penta-1,3-diynyl)-5-(3,4-dihydroxybut-1-ynyl)-thiophene = 5-(Penta-1,3-diynyl)-2-(3,4-dihydroxybut-1-ynyl)-thiophene (**9**)	*Echinops grijisii*, roots (Asteraceae)	MeOH/EtOAc-soluble fraction	230	C_13_H_10_O_2_S	Nantou, Taiwan	[28]
	*Echinops giganteus*, roots (Asteraceae)	MeOH/CH_2_Cl_2_-soluble fraction	-	-	Dschang, Western Region of Cameroon	[29]
	*Echinops giganteus*, rhizomes and aerial parts (Asteraceae)	MeOH/*n*-hexane-soluble fraction	-	-	Dschang, Western Region of Cameroon	[30]
	*Echinops ritro*, whole plant (Asteraceae)	EtOH/EtOAc-soluble fraction	-	-	Qinghe County, Xinjiang, China	[27]
2-(4-Hydroxybut-1-ynyl)-5-(penta-1,3-diynyl)thiophene (**10**)	*Echinops grijisii*, roots (Asteraceae)	MeOH/EtOAc-soluble fraction	214	C_13_H_10_OS	Nantou, Taiwan	[28]
2-(Penta-1,3-diyn-1-yl)-5–(4-acetoxy-3-hydroxybuta-1-yn-1-yl) thiophene (**11**)	*Pluchea indica*, aerial parts (Asteraceae)	EtOH/*n*-hexane-soluble fraction	256	C_15_H_12_O_2_S	Bon subdistrict, Khlung, Chantaburi, Thailand	[31]
5-(Penta-1,3-diynyl)-2-(3-hydroxy-4-acetoxybut-1-ynyl)-thiophene (**12**)	*Echinops hoehnelii*, roots (Asteraceae)	MeOH/CH_2_Cl_2_-soluble fraction	272	C_15_H_12_O_3_S	Bale Mountains National Park, Ethiopia	[32]
5-(Penta-1,3-diynyl)-2-(3-methoxy-4-acetoxy-but-1-yn)-thiophene (**13**)	*Echinops hoehnelii*, roots (Asteraceae)	MeOH/CH_2_Cl_2_-soluble fraction	286	C_16_H_14_O_3_S	Bale Mountains National Park, Ethiopia	[32]
5-(Penta-1,3-diynyl)-2-(3-chloro-4-acetoxy-but-1-yn)-thiophene (**14**)	*Echinops hoehnelii*, roots (Asteraceae)	MeOH/CH_2_Cl_2_-soluble fraction	290	C_15_H_11_ClO_2_S	Bale Mountains National Park, Ethiopia	[32]
Echinothiophene A (**15**)	*Echinops grijisii*, roots (Asteraceae)	EtOH/CH_2_Cl_2_-soluble fraction	248	C_13_H_9_ClOS	Zhangjiakou Hebei, China	[19]
Echinothiophene B (**16**)	*Echinops grijisii*, roots (Asteraceae)	EtOH/CH_2_Cl_2_-soluble fraction	290	C_15_H_11_ClO_2_S	Zhangjiakou Hebei, China	[19]
Echinothiophene C (**17**)	*Echinops* grijisii, roots (Asteraceae)	EtOH/CH_2_Cl_2_-soluble fraction	398	C_23_H_26_O_4_S	Zhangjiakou Hebei, China	[19]
2-(Pro-1-ynyl)-5-(5,6-dihydroxypenta-1,3-diynyl) thiophene (PYDDT) = 2-(Prop-1-ynyl)-5(5,6-dihydroxyhexa-1, 3-diynyl)-thiophene = PITC-2 = R/J/3 (**18**)	*Echinops grijisii*, roots (Asteraceae)	EtOH/CH_2_Cl_2_-soluble fraction	230	C_13_H_10_O_2_S	Bozhou north of Anhui, China	[33]
	*Pluchea indica*, aerial parts (Asteraceae)	EtOH/*n*-hexane-soluble fraction	-	-	Bon subdistrict, Khlung, Chantaburi, Thailand	[31]
5-(1,2-Dihydroxyethyl)-2-(*E*)-hept-5-ene-1,3-diynylthiophene (**19**)	*Echinops latifolius*, roots (Asteraceae)	MeOH/CH_2_Cl_2_-soluble fraction	232	C_13_H_12_O_2_S	Seoul, Korea	[34]
5-(1,2-Dihydroxy-ethyl)-2-(*Z*)-hept-5-ene-1,3-diynylthiophene (**20**)	*Echinops latifolius*, roots (Asteraceae)	MeOH/CH_2_Cl_2_-soluble fraction	232	C_13_H_12_O_2_S	Seoul, Korea	[34]
5-(Penta-1,3-diynyl)-2-(3-methoxy-4-hydroxybut-1-ynyl)-thiophen (**21**)	*Echinops hoehnelii*, roots (Asteraceae)	MeOH/CH_2_Cl_2_-soluble fraction	244	C_14_H_12_O_2_S	Bale Mountains National Park, Ethiopia	[32]
2-(Prop-1-inyl)-5-(6-acetoxy-5-hydroxyhexa-1,3-diinyl) thiophene (**22**)	*Pluchea indica*, aerial parts (Asteraceae)	EtOH/*n*-hexane-soluble fraction	256	C_15_H_12_O_2_S	Bon subdistrict, Khlung, Chantaburi, Thailand	[31]
53′′*R*-Pluthiophenol (**23**)	*Pluchea indica*, aerial parts (Asteraceae)	EtOH/*n*-hexane-soluble fraction	230	C_13_H_10_O_2_S	Hepu, Guangxi, China	[35]
3′′*R*-Pluthiophenol-4′′-acetate (**24**)	*Pluchea indica*, aerial parts (Asteraceae)	EtOH/*n*-hexane-soluble fraction	272	C_15_H_12_O_3_S	Hepu, Guangxi, China	[35]
3′′-Ethoxy-3′′*S*-pluthiophenol (**25**)	*Pluchea indica*, aerial parts (Asteraceae)	EtOH/*n*-hexane-soluble fraction	258	C_15_H_14_O_2_S	Hepu, Guangxi, China	[3]
3′′-Ethoxy-3′′*S*-pluthiophenol-4′′-acetate (**26**)	*Pluchea indica*, aerial parts (Asteraceae)	EtOH/*n*-hexane-soluble fraction	300	C_17_H_16_O_3_S	Hepu, Guangxi, China	[35]
Rupestriene B (**27**)	*Artemisia rupestris*, whole plant (Asteraceae)	EtOH/CH_3_Cl-soluble fraction	234	C_13_H_14_O_2_S	Xinjiang Uygur Autonomous, China	[36]
Rupestriene C (**28**)	*Artemisia rupestris*, whole plant (Asteraceae)	EtOH/CH_3_Cl-soluble fraction	234	C_13_H_14_O_2_S	Xinjiang Uygur Autonomous, China	[36]
Atracthioenyneside A (**29**)	*Atractylodes lancea*, rhizomes (Asteraceae)	EtOH/n-BuOH extract	414	C_19_H_26_O_8_S	Huanggang, Hubei, China	[37]
Atracthioenyneside B (**30**)	*Atractylodes lancea*, rhizomes (Asteraceae)	EtOH/n-BuOH extract	386	C_17_H_22_O_8_S	Huanggang, Hubei, China	[37]
(*Z*)-6-(5-(Prop-1-yn-1-yl)thiophen-2-yl)hex-3-en-5-yne-1,2-diol (**31**)	*Eclipta prostrata*, aerial parts (Asteraceae)	EtOH/PE-soluble fraction	232	C_13_H_12_O_2_S	Mount Kunyu area, Shandong, China	[38]
II. Bithiophenes						
5-(4-Hydroxy-3-methoxy-1-butyny)-2,2′-bithiophene (**32**)	*Echinops grijisii*, roots (Asteraceae)	MeOH/EtOAc-soluble fraction	264	C_13_H_12_O_2_S_2_	Nantou, Taiwan	[28]
5-(3,4-Dihydroxybut-1-ynyl)-2,2′-bithiophene = 4-([2,2′-bithiophen]-5-yl) but-3-yne-1,2-diol (**33**)	*Echinops grijisii*, roots (Asteraceae)	MeOH/EtOAc-soluble fraction	250	C_12_H_10_O_2_S_2_	Nantou, Taiwan	[28]
	*Eclipta prostrata*, aerial parts (Asteraceae)	MeOH/EtOAc-soluble fraction	-	-	Seoul, Korea	[39]
	*Echinops ritro*, whole plant (Asteraceae)	EtOH/EtOAc-soluble fraction	-	-	Qinghe County, Xinjiang, China	[27]
5-Acetyl-2,2′-bithiophene *=* Ethanone (**34**)	*Echinops grijisii*, roots (Asteraceae)	MeOH/EtOAc-soluble fraction	208	C_10_H_8_OS_2_	Nantou, Taiwan	[28]
	*Echinops ritro*, whole plant (Asteraceae)	EtOH/EtOAc-soluble fraction	-	-	Qinghe County, Xinjiang, China	[27]
5-Formyl-2,2′-bithiophene (**35**)	*Echinops grijisii*, roots (Asteraceae)	MeOH/EtOAc-soluble fraction	194	C_9_H_6_OS_2_	Nantou, Taiwan	[28]
Methyl 2,2′-bithiophene-5-carboxylate (**36**)	*Echinops grijisii*, roots (Asteraceae)	MeOH/EtOAc-soluble fraction	224	C_10_H_8_O_2_S_2_	Nantou, Taiwan	[28]
5-(But-3-en-1-ynyl)-2,2′-bithiophene (5-BBT) (**37**)	*Echinops grijisii*, roots (Asteraceae)	MeOH/EtOAc-soluble fraction	216	C_12_H_8_S_2_	Nantou, Taiwan	[28]
	*Porophyllum obscurum*, aerial parts (Asteraceae)	*n*-Hexane extract	-	-	Las Chacras, province of San Luis, Argentina	[40]
	*Echinops grijisii*, roots (Asteraceae)	Essential oil, hydrodistillation	-	-	Nanjing, Jiangsu, China	[41]
	*Echinops albicaulis*, aerial parts (Asteraceae)	MeOH/CH_2_Cl_2_ soluble fraction	-	-	Malaysary gorge, Kazakhstan	[42]
5-(4-Isovaleroyloxybut-1-ynyl)-2,2′-bithiophene (5-IBT) (**38**)	*Echinops grijisii*, roots (Asteraceae)	MeOH/EtOAc-soluble fraction	318	C_17_H_18_O_2_S_2_	Nantou, Taiwan	[28]
	*Echinops grijisii*, roots (Asteraceae)	Essential oil, hydrodistillation	-	-	Nanjing, Jiangsu, China	[41]
Cardopatine (**39**)	*Echinops grijisii*, roots (Asteraceae)	MeOH/EtOAc-soluble fraction	432	C_24_H_16_S_4_	Nantou, Taiwan	[28]
Isocardopatine (**40**)	*Echinops grijisii*, roots (Asteraceae)	MeOH/EtOAc-soluble fraction	432	C_24_H_16_S_4_	Nantou, Taiwan	[28]
5-(3-Hydroxy-4-isovaleroyloxybut-1-ynyl)-2,2′-bithiophene (**41**)	*Echinops grijisii*, roots (Asteraceae)	MeOH/EtOAc-soluble fraction	334	C_17_H_18_O_3_S_2_	Nantou, Taiwan	[28]
5-(3-Hydroxymethyl-3-isovaleroyloxyprop-1-ynyl)-2,2′-bithiophene (**42**)	*Echinops grijisii*, roots (Asteraceae)	MeOH/EtOAc-soluble fraction	334	C_17_H_18_O_3_S_2_	Nantou, Taiwan	[28]
5-(4-Hydroxy-1-butynyl)-2,2′-bithiophene *=* 4-([2,2′-bithiophen]-5-yl)but-3-yn-1-ol = 4-(5′-(hydroxymethyl)-[2,2′-bithiophene]-5-yl)but-3-yn-1-ol) (**43**)	*Echinops grijisii*, roots (Asteraceae)	MeOH/EtOAc-soluble fraction	234	C_12_H_10_OS_2_	Nantou, Taiwan	[28]
	*Porophyllum obscurum*, aerial parts (Asteraceae)	*n*-Hexane extract	-	-	Las Chacras, province of San Luis, Argentina	[40]
	*Echinops ritro*, whole plant (Asteraceae)	EtOH/EtOAc-soluble fraction	-	-	Qinghe County, Xinjiang, China	[27]
	*Tagetes minuta*, aerial parts (Asteraceae)	MeOH/*n*-hexane-soluble fraction	-	-	Al-Baha city, Saudi Arabia	[43]
5-(4-Acetoxy-1-butynl)-2,2′-bithiophene (**44**)	*Echinops grijisii*, roots (Asteraceae)	MeOH/EtOAc-soluble fraction			Nantou, Taiwan	[28]
	*Porophyllum obscurum*, aerial parts (Asteraceae)	*n*-Hexane extract	-	-	Las Chacras, province of San Luis, Argentina	[40]
2,2′-Bithiophene-5-carboxylic acid (**45**)	*Echinops grijisii*, roots (Asteraceae)	MeOH/EtOAc-soluble fraction	209	C_9_H_6_O_2_S_2_	Nantou, Taiwan	[28]
	*Echinops ritro*, whole plant (Asteraceae)	EtOH/EtOAc-soluble fraction	-	-	Qinghe County, Xinjiang, China	[27]
Thiotagetin B: [(*Z*)-1′′-([2,2′-bithiophen]-5-yl)-8′′-chloro-6′′,11′′-dimethylundeca-6′′,10′′-dien-2′′-yn-9′′-one] (**46**)	*Tagetes minuta*, aerial parts (Asteraceae)	MeOH/*n*-hexane-soluble fraction	388	C_21_H_21_ClOS_5_	Al-Baha city, Saudi Arabia	[44]
1-([2,2′-Bithiophen]-5-yl)-4 -hydroxybutan-1-one (**47**)	*Echinops ritro*, whole plant (Asteraceae)	EtOH/EtOAc-soluble fraction	252	C_12_H_12_O_2_S_2_	Qinghe County, Xinjiang, China	[27]
6-Methoxy-arctinol-b (**48**)	*Echinops latifolius*, roots (Asteraceae)	MeOH/CH_2_Cl_2_-soluble fraction	278	C_14_H_14_O_2_S_2_	Seoul, Korea	[34]
Arctinol-b (**49**)	*Echinops latifolius*, roots (Asteraceae)	MeOH/CH_2_Cl_2_-soluble fraction	264	C_13_H_12_O_2_S_2_	Seoul, Korea	[34]
	*Echinops ritro*, whole plant (Asteraceae)	EtOH/EtOAc-soluble fraction	-	-	Qinghe County, Xinjiang, China	[27]
Arctinone-b (**50**)	*Echinops grijisii*, roots (Asteraceae)	EtOH/CH_2_Cl_2_-soluble fraction	246	C_13_H_10_OS_2_	Zhangjiakou Hebei, China	[19]
Arctinol (**51**)	Echinops latifolius, roots (Asteraceae)	MeOH/CH_2_Cl_2_-soluble fraction	248	C_12_H_8_O_2_S_2_	Seoul, Korea	[34]
Arctinal (**52**)	*Echinops ritro*, whole plant (Asteraceae)	EtOH/EtOAc-soluble fraction	232	C_12_H_8_OS_2_	Qinghe County, Xinjiang, China	[27]
Arctinol A (**53**)	*Echinops ritro*, whole plant (Asteraceae)	EtOH/EtOAc-soluble fraction	234	C_12_H_10_OS_2_	Qinghe County, Xinjiang, China	[27]
Arctic acid (**54**)	*Echinops ritro*, whole plant (Asteraceae)	EtOH/EtOAc-soluble fraction	248	C_12_H_8_O_2_S_2_	Qinghe County, Xinjiang, China	[27]
Methyl [5′-(1-propynyf)-2,2′-bithienyl-5-yl] carboxylate (**55**)	*Echinops latifolius*, roots (Asteraceae)	MeOH/CH_2_Cl_2_-soluble fraction	262	C_13_H_10_O_2_S_2_	Seoul, Korea	[34]
2,2-Dimethyl-4-[5′-(prop-1-ynyl)-2,2′-bithiophen-5-yl]-1,3-dioxolane (**56**)	*Echinops spinosissimus* subsp. *Spinosus*, roots (Asteraceae)	EtOH/CH_2_Cl_2_-soluble fraction	304	C_16_H_16_O_2_S_2_	Morocco	[45]
5′-(3,4-Dihydroxybut-1-yn-1-yl)-[2,2′-bithiophene]-5-carbaldehyde = 5-[l-(4-hydroxybut-l-ynyl)]-2,2′-bithiophene-5′-carbaldehyde (**57**)	*Echinops ritro*, whole plant (Asteraceae)	EtOH/EtOAc-soluble fraction	278	C_13_H_10_O_3_S_2_	Qinghe County, Xinjiang, China	[27]
4-Hydroxy-1-(5′-methyl-[2,2′-bithiophen]-5-yl)butan-1-one (**58**)	*Echinops ritro*, whole plant (Asteraceae)	EtOH/EtOAc-soluble fraction	266	C_13_H_14_O_2_S_2_	Qinghe County, Xinjiang, China	[27]
5′-(3,4-Dihydroxybut-1-yn-1-yl)-[2,2′-bithiophene]-5-carboxylic acid (**59**)	*Echinops ritro*, whole plant (Asteraceae)	EtOH/EtOAc-soluble fraction	294	C_13_H_10_O_4_S_2_	Qinghe County, Xinjiang, China	[27]
4-(5′-Methyl-[2,2′-bithiophen]-5-yl)but-3-yn-1-ol (**60**)	*Echinops ritro*, whole plant (Asteraceae)	EtOH/EtOAc-soluble fraction	248	C_13_H_12_OS_2_	Qinghe County, Xinjiang, China	[27]
Echinothiophene D (**61**)	*Echinops grijisii*, roots (Asteraceae)	EtOH/CH_2_Cl_2_-soluble fraction	298	C_13_H_14_O_4_S_2_	Zhangjiakou Hebei, China	[19]
Echinothiophene E (**62**)	*Echinops grijisii*, roots (Asteraceae)	EtOH/CH_2_Cl_2_-soluble fraction	366	C_18_H_19_ClO_2_S_2_	Zhangjiakou Hebei, China	[19]
Echinothiophene F (**63**)	*Echinops grijisii*, roots (Asteraceae)	EtOH/CH_2_Cl_2_-soluble fraction	432	C_23_H_28_O_4_S_2_	Zhangjiakou Hebei, China	[19]
2-Prop-1-inyl-5′-(2-hydroxy-3-chloropropyl) dithiophene (**64**)	*Echinops grijisii*, roots (Asteraceae)	EtOH/CH_2_Cl_2_-soluble fraction	282	C_13_H_11_ClOS_2_	Zhangjiakou Hebei, China	[19]
Ecliprostin A (**65**)	*Eclipta prostrata*, aerial parts (Asteraceae)	EtOH/EtOAc-soluble fraction	348	C_18_H_20_O_3_S_2_	Mount Kunyu area, Shandong, China	[18]
Ecliprostin B (**66**)	*Eclipta prostrata*, aerial parts (Asteraceae)	EtOH/EtOAc-soluble fraction	348	C_18_H_20_O_3_S_2_	Mount Kunyu area, Shandong, China,	[18]
Ecliprostin C (**67**)	*Eclipta prostrata*, aerial parts (Asteraceae)	EtOH/EtOAc-soluble fraction	678	C_36_H_38_O_5_S_4_	Mount Kunyu area, Shandong, China	[18]
Echinbithiophenedimer A (**68**)	*Echinops latifolius*, roots (Asteraceae)	EtOH/*n*-hexane:acetone (8:1) soluble fraction	492	C_26_H_20_O_2_S_4_	Mentougou, Beijing, China	[17]
Echinbithiophenedimer B (**69**)	*Echinops latifolius*, roots (Asteraceae)	EtOH/*n*-hexane:acetone (8:1) soluble fraction	492	C_26_H_20_O_2_S_4_	Mentougou, Beijing, China	[17]
Echinbithiophenedimer C (**70**)	*Echinops latifolius*, roots (Asteraceae)	EtOH/*n*-hexane:acetone (8:1) soluble fraction	492	C_26_H_20_O_2_S_4_	Mentougou, Beijing, China	[17]
(*R*)-(5′-(3,4-dihydroxybut-1-yn-1-yl)-[2,2′-bithiophen]-5-yl)methyl 3-methylisovalerate (**71**)	*Eclipta prostrata*, aerial parts (Asteraceae)	EtOH/PE-soluble fraction	364	C_18_H_20_O_4_S_2_	Mount Kunyu area, Shandong, China	[38]
5-[l-(4-hydroxybut-l-ynyl)]-2,2′-bithiophene-5′-carbaldehyde (**72**)	*Eclipta prostrata*, aerial parts (Asteraceae)	EtOH/PE-soluble fraction	262	C_13_H_10_O_2_S_2_	Mount Kunyu area, Shandong, China	[38]
5′-Hydroxymethyl-5-(3-butene-1-ynyl)-2,2′-bithiophene (**73**)	*Eclipta prostrata*, aerial parts (Asteraceae)	EtOH/PE-soluble fraction	246	C_13_H_10_OS_2_	Mount Kunyu area, Shandong, China	[38]
4-(5′-(hydroxymethyl)-[2,2′-bithiophene]-5-yl)but-3-yn-1-ol) (Thio1) (**74**)	*Tagetes patula*, aerial parts (Asteraceae)	Synthesis	264	C_13_H_12_O_2_S_2_	-	[46]
III. Terthiophene						
2,2′:5′,2′′-Terthiophene (α-Terthienyl) (α-T) (**75**)	*Echinops grijisii*, roots (Asteraceae)	MeOH/EtOAc-soluble fraction	248	C_12_H_8_S_3_	Nantou, Taiwan	[28]
	*Eclipta prostrata*, aerial parts (Asteraceae)	MeOH/EtOAc-soluble fraction	-	-	Seoul, Korea	[39]
	*Porophyllum obscurum*, aerial parts (Asteraceae)	*n*-Hexane extract	-	-	Las Chacras, province of San Luis, Argentina	[40]
	*Echinops grijisii*, roots (Asteraceae)	Essential oil, hydrodistillation	-	-	Nanjing, Jiangsu, China	[41]
	*Echinops albicaulis*, aerial parts (Asteraceae)	MeOH/CH_2_Cl_2_ soluble fraction	-	-	Malaysary gorge, Kazakhstan	[42]
5-Formyl-2,2′:5′,2′′-terthiophene (Ecliptal) (**76**)	*Eclipta prostrata*, aerial parts (Asteraceae)	MeOH/EtOAc-soluble fraction	276	C_13_H_8_OS_3_	Seoul, Korea	[39]
	*Eclipta prostrata*, whole plant (Asteraceae)	EtOH/*n*-hexane-soluble fraction	-	-	Seoul, Korea	[47]
	Tagetes minuta, aerial parts (Asteraceae)	MeOH/*n*-hexane-soluble fraction	-	-	Al-Baha city, Saudi Arabia	[43]
5-Hydroxymethyl-2,2′:5′,2′′- terthiophene (α-Terthienylmethanol) (**77**)	*Eclipta prostrata*, aerial parts (Asteraceae)	MeOH/EtOAc-soluble fraction	278	C_13_H_10_OS_3_	Seoul, Korea	[39]
	*Eclipta prostrata*, whole plant (Asteraceae)	EtOH/*n*-hexane-soluble fraction	-	-	Seoul, Korea	[47]
	*Eclipta prostrata*, whole plant (Asteraceae)	EtOH/*n*-hexane-soluble fraction	-	-	Seoul, Korea	[20]
3′-Methoxy-2,2′:5′,2′′-terthiophene (**78**)	*Eclipta prostrata*, aerial parts (Asteraceae)	MeOH/EtOAc-soluble fraction	278	C_13_H_10_OS_3_	Seoul, Korea	[39]
5-Hydroxymethyl-(2,2′:5′,2′′)-terthienyl angelate (**79**)	*Eclipta prostrata*, whole plant (Asteraceae)	EtOH/*n*-hexane-soluble fraction	360	C_18_H_16_O_2_S_3_	Seoul, Korea	[47]
5-Hydroxymethyl-(2,2′:5′,2′′)-terthienyl tiglate (**80**)	*Eclipta prostrata*, whole plant (Asteraceae)	EtOH/*n*-hexane-soluble fraction	360	C_18_H_16_O_2_S_3_	Seoul, Korea	[47]
5-Methoxy-(2,2′:5′,2′′)-terthiophene (**81**)	*Eclipta prostrata*, whole plant (Asteraceae)	EtOH/*n*-hexane-soluble fraction	292	C_14_H_12_OS_3_	Seoul, Korea	[47]
3′-Hydroxy-2,2′:5′,2′′-terthiophene-3′-O-β-D-glucopyranoside (**82**)	*Eclipta prostrata*, aerial parts (Asteraceae)	MeOH/EtOAc-soluble fraction	426	C_18_H_18_O_6_S_3_	Seoul, Korea	[39]
IV. Quinquethiophenes						
Thiotagetin A (**83**)	Tagetes minuta, aerial parts, (Asteraceae)	MeOH/*n*-hexane-soluble fraction	540	C_27_H_24_O_2_S_5_	Al-Baha city, Saudi Arabia	[48]
V. Miscellaneous						
Sibiricumthionol (**84**)	*Xanthium sibiricum*, fruits (Asteraceae)	MeOH/CH_2_Cl_2_-soluble fraction	197	C_9_H_11_NO_2_S	Helen town, Heilongjiang, China	[49]
(+)-Xanthienopyran (**85**)	*Xanthium sibiricum*, fruits (Asteraceae)	MeOH/CH_2_Cl_2_-soluble fraction	316	C_17_H_16_O_4_S	Helen town, Heilongjiang, China	[49]
Rupestriene A (**86**)	*Artemisia rupestris*, whole plant (Asteraceae)	EtOH/CH_3_Cl-soluble fraction	280	C_15_H_20_O_3_S	Xinjiang Uygur Autonomous, China	[36]
7-[1-(Thiophene-5-yl)-1-formamido]-3-propylenyl-3-cephem-4-carboxylic acid (CAx1) (**87**)	Endolithic *Streptomyces* sp. AL51	Spore MeOH extract	350	C_15_H_14_N_2_O_4_S_2_	Granite rock, Mylliem, Meghalaya, India	[50]
2,5-Bis(5-tert-butyl-2-benzoxazolyl)thiophene (**88**)	Marine-derived actinomycete *Streptomyces* sp. G278 isolated from echinoderm *Holothuria edulis*	Fermentation broth	430	C_26_H_26_N_2_O_2_S	Cu Lao Cham- Quang Nam, Vietnam	[51]
Thiocarboxylic A (**89**)	*Penicillium* sp. sb62 was isolated from soil collected near the fibrous roots of *Schisandra bicolor* var. *tuberculata* (Schisandraceae)	EtOAc of fermented material	332	C_18_H_20_O_4_S	Xinning, Hunan, China	[16]
Thiocarboxylic B (**90**)	*Penicillium* sp. sb62 was isolated from soil collected near the fibrous roots of *Schisandra bicolor* var. *tuberculata* (Schisandraceae)	EtOAc of fermented material	376	C_19_H_20_O_6_S	Xinning, Hunan, China	[16]
Thiocarboxylic C1 (**91**)	*Penicillium* sp. sb62 was isolated from soil collected near the fibrous roots of *Schisandra bicolor* var. *tuberculata* (Schisandraceae)	EtOAc of fermented material	348	C_18_H_20_O_5_S	Xinning, Hunan, China	[16]
Thiocarboxylic C2 (**92**)	*Penicillium* sp. sb62 was isolated from soil collected near the fibrous roots of *Schisandra bicolor* var. *tuberculata* (Schisandraceae)	EtOAc of fermented material	348	C_18_H_20_O_5_S	Xinning, Hunan, China	[16]
Thiocarboxylic D1 (**93**)	*Penicillium* sp. sb62 was isolated from soil collected near the fibrous roots of *Schisandra bicolor* var. *tuberculata* (Schisandraceae)	EtOAc of fermented material	350	C_18_H_22_O_5_S	Xinning, Hunan, China	[16]
Thiocarboxylic D2 (**94**)	*Penicillium* sp. sb62 was isolated from soil collected near the fibrous roots of *Schisandra bicolor* var. *tuberculata* (Schisandraceae)	EtOAc of fermented material	350	C_18_H_22_O_5_S	Xinning, Hunan, China	[16]
Rupestriene D (**95**)	*Artemisia rupestris*, whole plant (Asteraceae)	EtOH/PE-soluble fraction	234	C_13_H_14_O_2_S	Xinjiang Uygur Autonomous, China	[15]
Rupestriene E (**96**)	*Artemisia rupestris*, whole plant (Asteraceae)	EtOH/EtOAc-soluble fraction	294	C_15_H_18_O_4_S	Xinjiang Uygur Autonomous, China	[15]

**Table 3:** The probability and model accuracy prediction for thiophene derivatives against cathepsin D using the SuperPred target prediction webserver.

Compound	Probability *	Model Accuracy **
9	96.04%	96.09%
20	93.69%	96.09%
28	82.30%	98.95%
29	85.79%	98.95%
30	82.76%	98.95%
33	87.63%	98.95%
43	82.00%	98.95%
46	94.79%	98.95%
57	89.12%	98.95%
67	91.20%	98.95%
68	77.77%	90.17%
70	88.21%	98.95%
75	79.91%	98.95%
76	87.50%	98.95%
77	85.75%	98.95%
80	90.87%	98.95%
81	90.32%	98.95%
82	88.71%	98.95%

* The input structure binds with the specific target, as determined by the respective target machine learning model. ** Since the model performance varies across different targets, additionally, the 10-fold cross-validation score of the respective logistic regression model is displayed.

**Table 4:** In silico predicted ADME properties of the thiophenes derivatives.

Title	mol MW	# Stars	Dipole	SASA	DonorHB	AccptHB	QPlogPo/w	QPlogS	QPlogKhsa	#Metab	QPlogBB	% HumOral Absorption	QPlogHERG	CNS	#RtvFG
Recommended range	(130–725)	(0.0–5.0)	(1–12.50)	(300–1000)	(0–6)	(2.0–20.0)	(−2–6.5)	(−6.5–0.5)	(−1.5–1.5)	(1–8)	(−3–1.2)	(<25% poor; >80% high)	concern below −5	(−2 inactive) (+2 active)	(0–2)
1	170.23	1	2.8	375.85	1	2	2.15	−2.47	−0.34	4	−0.19	83.82	−1.25	−1	-
2	218.33	-	7.35	456.52	-	4	2.1	−1.26	−0.4	6	0.01	75.45	−3.63	1	-
3	186.33	5	3.11	416.69	-	0.5	3.73	−4.49	0.38	5	0.61	100	−3.22	2	-
4	310.53	7	0.48	576.92	-	0.5	5.35	−8.24	1.34	10	0.96	100	−3.9	2	-
5	202.33	-	5.95	441.45	-	4	2.01	−1.08	−0.44	6	0.24	78.76	−3.53	1	-
6	188.35	5	2.31	409.89	-	1	3.11	−3.82	0.3	3	0.45	100	−2.88	1	-
7	166.19	-	3.67	386.76	1	2	1.96	−1.92	−0.42	2	−0.4	80.8	−2.35	−1	-
8	180.22	3	6.7	417.32	-	1.75	2.21	−2.75	−0.11	3	−0.39	95.04	−4.18	-	-
9	230.28	-	3.09	547.44	2	3.4	2.52	−4.12	−0.08	4	−0.8	95.42	−5.92	−1	-
10	214.28	1	2.07	536.85	1	1.7	3.7	−4.76	0.3	4	−0.22	100	−5.9	-	-
11	256.32	-	3.01	596.59	-	2.7	3.92	−5.33	0.4	4	−0.73	100	−6.01	−1	1
12	272.32	-	4.76	640.28	1	3.7	3.61	−5.82	0.34	3	−1.03	100	−6.49	−2	1
13	286.35	-	4.06	664.62	-	3.7	4.35	−6.09	0.44	4	−0.45	100	−6.48	-	1
14	290.76	1	5.25	642.37	-	2	5.2	−7.01	0.83	3	−0.18	100	−6.36	-	2
15	248.73	1	5.08	543.19	1	1.7	4.16	−5.53	0.46	3	0.24	100	−5.84	1	-
16	290.76	1	3.84	623.15	-	2	5.14	−6.93	0.85	3	0.1	100	−6.22	1	1
17	398.52	2	2.11	825.87	-	4	6.39	−8.4	1.32	5	−0.97	100	−6.6	−1	2
18	230.28	-	3.09	548.17	2	3.4	2.52	−4.13	−0.08	4	−0.8	95.44	−5.93	−1	-
19	232.3	-	3.49	538.9	2	3.4	2.64	−3.94	−0.06	4	−0.61	100	−5.6	-	-
20	232.3	-	3.7	536.62	2	3.4	2.57	−3.9	−0.06	4	−0.67	100	−5.55	-	-
21	244.31	-	2.85	572.08	1	3.4	3.53	−4.68	0.15	5	−0.27	100	−5.91	-	-
22	256.32	-	3.42	600.19	-	2.7	3.91	−5.41	0.4	4	−0.77	100	−6.08	−1	1
23	230.28	-	3.18	547.88	2	3.4	2.52	−4.13	−0.08	4	−0.8	95.43	−5.93	−1	-
24	272.32	-	4.71	639.63	1	3.7	3.61	−5.81	0.34	3	−1.03	100	−6.48	−2	1
25	258.33	-	2.66	619.03	1	3.4	4	−5.37	0.3	5	−0.35	100	−6.24	-	-
26	300.37	2	4.36	710.36	-	3.7	4.83	−6.8	0.61	4	−0.55	100	−6.73	-	1
27	234.31	-	3.3	548.83	-	2.7	3.45	−4.13	0.11	5	−0.82	100	−5.6	−1	1
28	234.31	1	6.27	543.84	-	2.7	3.44	−4.03	0.09	5	−0.77	100	−5.53	−1	1
29	414.47	2	4.68	754.8	6	13.6	−0.11	−2.92	−1.06	8	−3.29	40.05	−5.89	−2	1
30	386.42	1	1.2	710.72	6	13.6	−0.73	−2.7	−1.18	7	−3.22	35.33	−5.91	−2	1
31	232.3	-	2.7	541.33	2	3.4	2.43	−3.82	−0.08	4	−0.79	95.57	−5.54	−1	-
32	264.36	-	1.93	538.34	1	3.4	3.54	−4.4	0.13	5	−0.08	100	−5.56	-	-
33	250.33	-	2.38	509.39	2	3.4	2.66	−3.76	−0.09	4	−0.56	96.23	−5.48	-	-
34	208.29	-	4.09	422.53	-	2	3.01	−3.34	0.01	2	0.23	100	−4.42	1	-
35	194.27	-	4.61	394.08	-	2	2.75	−2.53	−0.14	2	0.03	100	−4.24	1	-
36	224.29	-	3.29	451.72	-	2	3.15	−3.89	0.14	2	0.14	100	−4.8	1	-
37	216.32	5	0.44	474.39	-	-	5.8	−5.65	0.72	2	0.35	100	−5.56	1	-
38	318.45	1	3.17	674.19	-	2	5.85	−7.32	1.08	4	−0.2	100	−6.41	-	1
39	432.63	9	0.65	807.6	-	-	11.73	−12.03	2.41	6	0.5	100	−8.14	2	-
40	432.63	8	0.93	700.3	-	-	9.78	−11.17	2.17	6	0.52	100	−6.51	2	-
41	334.45	-	4.91	630.13	1	3.7	4.38	−5.51	0.58	4	−0.77	100	−5.67	−1	1
42	334.45	-	4.4	673.42	1	3.7	4.8	−6.38	0.68	5	−0.64	100	−6.24	-	1
43	234.33	1	2.01	498.72	1	1.7	3.71	−4.39	0.27	4	−0.03	100	−5.45	-	-
44	276.37	-	2.76	592.07	-	2	4.28	−6.08	0.67	3	−0.21	100	−6.15	-	1
45	210.27	-	3.5	401.73	1	2	2.72	−3.04	−0.23	2	−0.22	85.2	−2.48	−1	-
46	388.97	1	6.57	609.65	-	2	6.37	−6.02	1.17	8	0.12	100	−4.58	1	2
47	252.35	-	4.52	495.92	1	3.7	2.67	−3.53	−0.07	4	−0.5	96.52	−4.88	-	-
48	278.38	-	2.94	560.83	1	3.4	3.75	−4.74	0.27	6	−0.12	100	−5.36	-	-
49	264.36	-	2.49	532.94	2	3.4	2.98	−4.12	0.04	5	−0.44	100	−5.28	-	-
50	246.34	-	5.04	515.59	-	2	3.88	−4.92	0.41	3	0.1	100	−5.25	1	-
51	248.31	-	4.21	494.76	1	2	3.56	−4.39	0.13	3	−0.42	90.16	−3.34	−1	-
52	232.32	-	5.54	487.09	-	2	3.35	−4.29	0.26	3	−0.13	100	−5.11	-	-
53	234.33	1	1.8	496.6	1	1.7	3.64	−4.49	0.3	4	0.05	100	−5.21	1	-
54	248.31	-	4.21	495.1	1	2	3.57	−4.39	0.13	3	−0.42	90.17	−3.35	−1	-
55	262.34	-	4.22	544.7	-	2	4.12	−5.47	0.54	3	0	100	−5.55	-	-
56	304.42	4	1.89	591.79	-	1.5	5.59	−6.89	1.06	4	0.15	100	−5.48	1	1
57	278.34	1	3.43	547.55	2	5.4	1.7	−3.69	−0.27	4	−1.47	77.15	−5.45	−2	-
58	266.37	-	4.99	531.75	1	3.7	2.98	−4.13	0.1	5	−0.56	100	−4.91	-	-
59	294.34	1	1.92	555.27	3	5.4	1.93	−3.98	−0.45	4	−1.78	62.81	−3.68	−2	-
60	248.36	1	2.19	534.06	1	1.7	4.03	−5.03	0.44	5	−0.06	100	−5.44	-	-
61	298.37	-	5.85	542.89	3	7.1	1.07	−2.98	−0.5	5	−1.4	74.44	−4.8	−2	-
62	366.92	3	3.08	712.65	-	2	6.67	−8.41	1.37	5	0.21	100	−6.17	1	1
63	432.59	4	5.23	855.53	-	4	7	−9.13	1.52	6	−0.86	100	−6.63	−1	2
64	282.8	1	5.1	546.49	1	1.7	4.64	−5.5	0.57	4	0.34	100	−5.39	1	-
65	348.47	-	4.77	685.58	1	3.7	4.9	−6.42	0.76	5	−0.85	100	−5.81	−1	1
66	348.47	1	5.79	721.29	1	3.7	5.08	−7.07	0.82	6	−0.93	100	−6.37	−1	1
67	678.93	10	5.34	1180.96	-	5.7	10.73	−13.34	2.73	11	−1.63	100	−8.35	−2	2
68	492.68	5	2.25	916.97	-	3.4	9.48	−11.13	2.07	8	−0.68	100	−8.28	-	1
69	492.68	5	2.32	856.86	-	3.4	8.46	−10.47	1.88	8	−0.68	100	−7.68	-	1
70	492.68	6	1.67	848.1	-	3.4	8.4	−10.35	1.93	10	−0.68	100	−7.54	-	-
71	364.47	-	4.17	731.03	2	5.4	3.97	−6.33	0.43	6	−1.58	95.78	−6.38	−2	1
72	262.34	1	4.69	537.04	1	3.7	2.72	−4.24	0.05	4	−0.92	91.26	−5.42	−1	-
73	246.34	1	2.19	521.45	1	1.7	3.88	−4.78	0.38	3	−0.03	100	−5.63	-	-
74	264.36	-	2.47	545.82	2	3.4	2.92	−4.18	0.02	5	−0.73	96.95	−5.5	−1	-
75	248.38	6	0.96	464.31	-	-	5.71	−7.4	0.83	3	0.19	100	−5.33	1	-
76	276.39	1	4.61	502.07	-	2	3.95	−4.98	0.46	3	0.03	100	−5.34	1	-
77	278.4	1	2.58	511.2	1	1.7	4.22	−5.06	0.5	4	0.2	100	−5.44	1	-
78	278.4	3	1.84	505.75	-	0.75	5.62	−5.96	0.83	4	0.03	100	−5.34	1	-
79	360.5	1	2.73	671.68	-	2	6.27	−7.84	1.29	6	0.18	100	−6.45	1	1
80	360.5	1	3.82	678.33	-	2	6.24	−7.98	1.3	6	0.08	100	−6.53	1	1
81	292.43	2	2.1	542.23	-	1.7	5.44	−5.81	0.72	5	−0.31	100	−5.61	-	-
82	426.52	-	3.15	644.26	4	9.25	2.04	−4.08	−0.27	7	−1.16	83.97	−5.71	−2	1
83	540.79	6	4.22	945.88	-	2	9.78	−12.95	2.68	6	−0.12	100	−8.05	-	-
84	197.25	-	6.72	419.53	1	4.7	1.09	−2.13	−0.51	4	−0.42	86.7	−3.64	-	-
85	316.37	1	3.62	593.36	2	5.45	2.42	−4.27	0.05	6	−1.29	85.52	−5.02	−2	1
86	280.38	-	4.82	549.83	1	5.4	2.64	−3.42	−0.09	5	−0.73	95.86	−4.44	−1	-
87	350.41	-	9.97	600.63	1.25	6.25	3.04	−4.55	−0.14	3	−1.21	76.31	−3.41	−2	1
88	432.58	2	2.52	761.06	1	3.75	6.09	−7.38	1.59	3	0.48	100	−6.96	2	-
89	332.41	-	3.73	648.07	-	4.75	4.13	−5.57	0.45	3	−0.34	100	−5.16	-	-
90	376.42	-	5.82	693.39	1	6.75	3.51	−5.75	0.2	3	−1.6	76.93	−3.34	−2	-
91	348.41	-	2.67	673.35	1	6.45	3.34	−5.55	0.28	3	−0.88	100	−5.43	−1	-
92	348.41	-	5.79	664.93	1	6.45	3.2	−5.39	0.28	3	−1.03	94.16	−5.22	−2	-
93	350.43	-	2.82	679.38	1	6.45	3.41	−5.5	0.28	4	−0.95	100	−5.29	−1	-
94	350.43	-	4.86	665.38	1	6.45	3.35	−5.25	0.26	4	−0.91	100	−5.09	−1	-
95	234.31	-	6.4	449.04	-	3.7	2.41	−2.95	−0.18	5	0.28	100	−3.85	1	-
96	294.37	-	6.75	555.68	1	5.7	2.37	−3.57	−0.1	5	−0.9	90.35	−4.57	−1	1

# Stars: number of property or descriptor values that fall outside the 95% range of similar values for known drugs. Many stars suggest that a molecule is less drug-like compared to molecules with few stars. Dipole: computed dipole moment of the molecule. SASA: total solvent accessible surface area (SASA) in square angstroms using a probe with a 1.4 Å radius. Donor H-bond: estimated number of hydrogen bonds that the solute would donate to water molecules in an aqueous solution. Acceptor H-bond: estimated number of hydrogen bonds that the solute would accept from water molecules in an aqueous solution. QPlogPo/w: predicted octanol/water partition coefficient. QPlogS: predicted aqueous solubility, log S. QPlogkhsa: prediction of binding to human serum albumin. # Metab: number of likely metabolic reactions.; QplogBB: Predicted brain/blood partition coefficient; % Human Oral Absorption: Predicted human oral absorption on 0 to 100% scale; QPlogHERG: predicted IC_50_ value for blockage of HERG K^+^ channels. CNS: predicted central nervous system activity on a −2 (inactive) to +2 (active) scale. # rtvFG: Number of reactive functional groups; the specific groups are listed in the jobname out file. The presence of these groups can lead to false positives in HTS assays and to decomposition, reactivity, or toxicity problems in vivo.

**Table 5:** In silico screening results of thiophene derivatives against cathepsin D (PDB: 4OD9).

Compd	Docking Score	XP GScore	Glide GScore	Glide Emodel
29	−9.439	−9.439	−9.439	−62.9
30	−9.178	−9.178	−9.178	−61.357
82	−8.09	−8.09	−8.09	−64.014
42	−6.971	−6.971	−6.971	−45.831
Ref_4OD9	−6.895	−7.306	−7.306	−71.557
66	−6.889	−6.889	−6.889	−56.795
64	−6.772	−6.772	−6.772	−47.815
61	−6.677	−6.677	−6.677	−45.925
39	−6.567	−6.567	−6.567	−68.455
40	−6.567	−6.567	−6.567	−68.455
57	−6.484	−6.484	−6.484	−44.895
33	−6.478	−6.478	−6.478	−37.519
46	−6.385	−6.385	−6.385	−54.121
19	−6.374	−6.374	−6.374	−39.754
81	−6.373	−6.373	−6.373	−40.88
92	−6.297	−6.297	−6.297	−50.1
9	−6.119	−6.119	−6.119	−37.184
52	−6.056	−6.056	−6.056	−39.105
2	−5.946	−5.946	−5.946	−35.558
89	−5.913	−5.913	−5.913	−41.976
68	−5.91	−5.91	−5.91	−58.146
59	−5.879	−5.879	−5.879	−43.627
58	−5.789	−5.789	−5.789	−39.479
41	−5.775	−5.778	−5.778	−50.687
84	−5.761	−5.761	−5.761	−35.037
31	−5.624	−5.624	−5.624	−39.484
17	−5.589	−5.589	−5.589	−58.566
91	−5.563	−5.563	−5.563	−39.688
65	−5.534	−5.534	−5.534	−52.028
74	−5.522	−5.522	−5.522	−50.032
49	−5.439	−5.439	−5.439	−37.501
53	−5.431	−5.431	−5.431	−42.062
60	−5.413	−5.413	−5.413	−48.483
32	−5.359	−5.359	−5.359	−36.918
37	−5.344	−5.344	−5.344	−38.255
95	−5.323	−5.733	−5.733	−31.554
12	−5.317	−5.32	−5.32	−48.638
93	−5.294	−5.294	−5.294	−41.169
71	−5.234	−5.234	−5.234	−56.25
77	−5.182	−5.182	−5.182	−47.296
80	−5.175	−5.175	−5.175	−52.89
20	−5.032	−5.032	−5.032	−38.495
47	−4.988	−4.988	−4.988	−41.313
48	−4.959	−4.959	−4.959	−45.598
79	−4.959	−4.959	−4.959	−53.558
87	−4.892	−4.892	−4.892	−42.972
70	−4.788	−4.788	−4.788	−66.838
16	−4.761	−4.761	−4.761	−39.898
22	−4.708	−4.708	−4.708	−43.587
76	−4.681	−4.681	−4.681	−42.185
50	−4.616	−4.616	−4.616	−42.266
62	−4.615	−4.615	−4.615	−53.205
38	−4.587	−4.587	−4.587	−44.277
21	−4.479	−4.479	−4.479	−34.302
96	−4.434	−4.844	−4.844	−43.679
27	−4.427	−4.427	−4.427	−32.461
54	−4.4	−4.4	−4.4	−42.328
36	−4.372	−4.372	−4.372	−31.206
34	−4.367	−4.367	−4.367	−32.958
10	−4.36	−4.36	−4.36	−37.183
4	−4.325	−4.325	−4.325	−44.054
55	−4.289	−4.289	−4.289	−41.301
86	−4.263	−4.673	−4.673	−34.699
67	−4.242	−4.242	−4.242	−71.461
72	−4.221	−4.221	−4.221	−45.333
56	−4.166	−4.166	−4.166	−45.866
18	−4.159	−4.159	−4.159	−38.902
28	−4.131	−4.131	−4.131	−38.886
51	−4.127	−4.127	−4.127	−38.518
73	−4.109	−4.109	−4.109	−41.377
88	−4.09	−4.09	−4.09	−46.666
94	−4.085	−4.085	−4.085	−40.186
35	−4.079	−4.079	−4.079	−30.767
44	−4.047	−4.047	−4.047	−41.625
75	−4.012	−4.012	−4.012	−39.751
69	−3.964	−3.964	−3.964	−65.916
85	−3.899	−3.899	−3.899	−35.518
83	−3.778	−3.778	−3.778	−63.675
13	−3.769	−3.769	−3.769	−38.792
9	−3.688	−3.688	−3.688	−41.071
23	−3.688	−3.688	−3.688	−41.071
14	−3.65	−3.65	−3.65	−38.638
11	−3.64	−3.64	−3.64	−42.16
43	−3.528	−3.528	−3.528	−37.644
25	−3.481	−3.481	−3.481	−39.665
24	−3.48	−3.483	−3.483	−44.761
78	−3.406	−3.406	−3.406	−37.385
45	−3.403	−3.403	−3.403	−30.006
3	−3.303	−3.303	−3.303	−26.546
15	−3.253	−3.253	−3.253	−35.921
90	−3.239	−3.239	−3.239	−43.733
26	−3.185	−3.185	−3.185	−40.847
8	−2.058	−2.117	−2.117	−23.999
63	−1.85	−1.85	−1.85	−70.342
7	−1.55	−1.55	−1.55	−18.635
5	−0.841	−0.841	−0.841	−10.861

## Data Availability

No new data were created or analyzed in this study. Data sharing is not applicable to this article.

## Supplementary Materials

The following supporting information can be downloaded at: https://www.mdpi.com/article/10.3390/plants11040539/s1, Table S1: Physical and spectral data of newly reported naturally occurring thiophenes from 2015 to 2021.

## List of Abbreviations

A2780: human ovarian cancer cell lines; 1,3-DPBF: 1,3-Diphenylisobenzofuran: 5-LOX: 5-Lipoxygenase; AChE: Acetylcholinesterase; ACS: American Chemical Society; ADMET: absorption/distribution/metabolism/excretion/and toxicity; AI: artificial intelligence; ATC: anatomical therapeutic chemical; Bax: Bcl-2-associated X protein; 5-BBT: 5-(But-3-en-1-ynyl)-2,2′-bithiophene; Bcl-2: B-cell lymphoma 2; BGC-823: human gastric cancer cell line; BuOH: butanol; CCRF-CEM: human leukemic cell line; CEM/ADR5000: human T-cell lymphoblast-like cell line; COSY: homonuclear correlation spectroscopy; CH_2_Cl_2_: Dichloromethane; CTLC: preparative centrifugal thin layer chromatography; CYP2A6: cytochrome P450 2A6; CYP2A13: cytochrome P450 2A13; DPPH: 2,2′-Diphenyl-2-picrylhydrazyl; EC_50_: effective concentration 50%; ECCD: exciton-coupled circular dichroism; ECD: electronic circular dichroism; ED_50_: effective dose 50; EIMS: electron impact mass spectrometry; ELISA: enzyme-linked immunosorbent assay; ERK1/2: extracellular signal regulated kinase 1/2; ES2*:* human ovarian cancer cell lines; ET-743: ecteinascidin 743; EtOH: ethanol; EtOAc: ethyl acetate; GSH: glutathione: HCT-116: human colon cancer cell line; Hec1A: human endometrial cancer cell line: HepG2: human liver cancer cell line; HERG K^+^: human ether-a-go-go-related gene potassium; HIV-1: human immunodeficiency virus-1; HMBC: heteronuclear multiple bond correlation; HPLC: high pressure liquid chromatography; HSQC: heteronuclear single quantum coherence; HRDARTMS: high-resolution direct analysis real time mass spectrometry; HRESIMS: high-resolution electrospray ionization mass spectrometry; 5-IBT: 5-(4-Isovaleroyloxybut-1-ynyl)-2,2′-bithiophene; IC_50_: half-maximal inhibitory concentration; IR: infrared; INT: *p*-iodonitrotetrazolium: IL-6: Interleukin-6; IR: infrared; Ishikawa: human endometrial cancer cell line: J2s: Second-stage juveniles; JNK: c-Jun N-terminal kinase; K562: human erythroleukemic cell line; KB: human oral epithelial-like cell; NFκB: nuclear factor kappa-light-chain-enhancer of activated B cells; LC_50_: lethal concentration 50%; LD_50_ is the amount of a material, which causes the 50% death; LC_95_: lethal dose 95%, which causes the death of 95% of a group of test animals; 5-LOX: 5-Lipoxygenase; LPS: Lipopolysaccharide; MCF-7: human breast cancer cell line; MFC: minimal fungicide concentration; MeOH: methanol; MIC: minimum inhibitory concentration; MBC: minimum bactericidal concentration; MDA-MB-231: human breast cancer cell line; NCI-H1650: human lung cancer cell line; MD: molecular dynamics; MDR: multidrug resistant; MTT: (3-(4,5-Dimethylthiazol-2-yl)-2,5-diphenyltetrazolium bromide); NADPH: nicotinamide–adenine dinucleotide phosphate: NBT: nitro blue tetrazolium; NRC: nitrite relative concentration; NMR: nuclear magnetic resonance; NO: nitric oxide; NP: natural protein; NSAIDs: non-steroidal anti-inflammatory drugs; OVCAR3*:* human ovarian cancer cell lines; PARP: poly ADP ribose polymerase; PCB: precursor-based combinatorial biosynthesis PDB: Protein Data Bank; PE: petroleum ether; p-H2AX: H2A histone family member X; PHA: phytohemagglutinin; PTLC: preparative thin layer chromatography: PYDDT: 2-(Pro-1-ynyl)-5-(5,6-dihydroxypenta-1,3-diynyl) thiophene; ROS: reactive oxygen species; RP CC: reversed phase column chromatography; RMSD: root mean square deviation; ROESY: rotating frame Overhauser effect spectroscopy; SAM: S-adenosylmethionine; SKOV3: human ovarian cancer cell lines; siRNAs: small interfering ribonucleic acid; SiO_2_ CC: silica gel column chromatography; SW620: human colon cancer cells line; SP600125: potent and reversible inhibitor of JNK1-3; α-T: α-Terthienyl; TNF-α: tumor necrosis factor-α; UV: ultraviolet; VS: virtual screening.
