# Correction: Ibrahim et al. Thiophenes—Naturally Occurring Plant Metabolites: Biological Activities and In Silico Evaluation of Their Potential as Cathepsin D Inhibitors. *Plants* 2022, *11*, 539

**DOI:** 10.3390/plants15152298

**Published:** 2026-07-27

**Authors:** Sabrin R. M. Ibrahim, Abdelsattar M. Omar, Alaa A. Bagalagel, Reem M. Diri, Ahmad O. Noor, Diena M. Almasri, Shaimaa G. A. Mohamed, Gamal A. Mohamed

**Affiliations:** 1 Department of Chemistry, Preparatory Year Program, Batterjee Medical College, Jeddah 21442, Saudi Arabia; 2 Department of Pharmacognosy, Faculty of Pharmacy, Assiut University, Assiut 71526, Egypt; 3 Department of Pharmaceutical Chemistry, Faculty of Pharmacy, King Abdulaziz University, Jeddah 21589, Saudi Arabia; asmansour@kau.edu.sa; 4 Center for Artificial Intelligence in Precision Medicines, King Abdulaziz University, Jeddah 21589, Saudi Arabia; 5 Department of Pharmaceutical Chemistry, Faculty of Pharmacy, Al-Azhar University, Cairo 11884, Egypt; 6 Department of Pharmacy Practice, Faculty of Pharmacy, King Abdulaziz University, Jeddah 21589, Saudi Arabia; abagalagel@kau.edu.sa (A.A.B.); rdiri@kau.edu.sa (R.M.D.); aonoor@kau.edu.sa (A.O.N.); dalmasri@kau.edu.sa (D.M.A.); 7 Faculty of Dentistry, British University, Suez Desert Road, Cairo 11837, Egypt; shaimaag1973@gmail.com; 8 Department of Natural Products and Alternative Medicine, Faculty of Pharmacy, King Abdulaziz University, Jeddah 21589, Saudi Arabia; gahussein@kau.edu.sa

**Error in Figure 1**

In the original publication [1], there was a mistake in Figure 1 as published. The genus ‘Atractylodes’ appeared twice on the x-axis due to an unintentional labeling repetition during figure preparation. The compound counts were not duplicated. The corrected Figure 1 appears below.

**Text Correction**

The original publication contained errors requiring correction, together with minor editorial issues requiring textual refinement. The following corrections and editorial revisions have been made.

A correction has been made to Section 5.4 (Molecular Dynamics Simulation) to clarify the RMSD description and statistics. The Cα-backbone RMSD transiently exceeded 3 Å toward the end of the 100 ns simulation, reaching approximately 4.0 Å. The backbone RMSD ranged from approximately 0.5 Å (minimum) to approximately 4.0 Å (maximum), with an overall mean of approximately 2.3 ± 0.6 Å. During the final 20 ns of the simulation, the backbone RMSD averaged approximately 2.2 ± 0.3 Å. The text has been revised to state that the complex remained mostly stable within 1–3 Å RMSD, with a transient rise to ~4 Å toward the end of the 100 ns simulation.

In addition, the description of the RMSD stability range has been revised to clarify that RMSD thresholds are system-dependent and not fixed universal cutoffs.

Furthermore, in Section 5.4, the ligand was incorrectly referred to as compound **29**. This typographical error has been corrected to compound **30**, which corresponds to the ligand used in the molecular dynamics simulation and presented in Figures 17 and 18.

In addition, minor editorial revisions have been made throughout the manuscript, including language polishing, English editing, formatting improvements, terminology standardization, stylistic refinements, and clarification of several sentences to improve readability and consistency. These editorial revisions do not affect the scientific data, interpretation of the results, or the conclusions of the review.

The authors apologize for any inconvenience caused and state that the scientific conclusions are unaffected. This correction was approved by the Academic Editor. The original publication has also been updated.

## Figures and Tables

**Figure 1:**
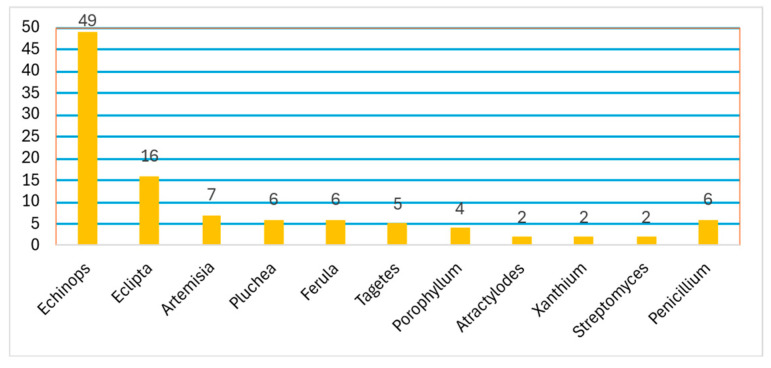
Number of reported thiophenes from various sources.
