# Supplementary material for: Thiophenes—Naturally Occurring Plant Metabolites: Biological Activities and In Silico Evaluation of Their Potential as Cathepsin D Inhibitors
Source: Plants (Basel). 2022 Feb 17;11(4):539. doi: 10.3390/plants11040539 (PMC8877444; doi:10.3390/plants11040539)
Supplement: Supplementary file 1 [file plants-11-00539-s001.zip › plants-1577412-supplementary.pdf]

plants

Review

Thiophenes Plant Constituents with Great Promises: Sources, Characterization, Biosynthesis, Biological Activities, and *In silico* Evaluation of Their Potency as Cathepsin D Inhibitors

Sabrin R.M. Ibrahim [1,2*], Abdelsattar M. Omar [3,4,5], Alaa A. Bagalagel [6], Reem M. Diri [6], Ahmad O. Noor [6], Diena M. Almasri [6], Shaimaa G.A. Mohamed [7], Gamal A. Mohamed [8]

[1] Department of Chemistry, Preparatory Year Program, Batterjee Medical College, Jeddah 21442, Saudi Arabia; sabrin.ibrahim@bmc.edu.sa
[2] Department of Pharmacognosy, Faculty of Pharmacy, Assiut University, Assiut 71526, Egypt; sabreen.ibrahim@pharm.aun.edu.eg
[3] Department of Pharmaceutical Chemistry, Faculty of Pharmacy, King Abdulaziz University, Jeddah 21589, Saudi Arabia; asmansour@kau.edu.sa
[4] Center for Artificial Intelligence in Precision Medicines, King Abdulaziz University, Jeddah 21589, Saudi Arabia.
[5] Department of Pharmaceutical Chemistry, Faculty of Pharmacy, Al-Azhar University, Nasr City, Cairo, Egypt.
[6] Department of Pharmacy Practice, Faculty of Pharmacy, King Abdulaziz University, Jeddah 21589, Saudi Arabia; abagalagel@kau.edu.sa (A.A.B.), rdiri@kau.edu.sa (R.M.D), Aonoor@kau.edu.sa (A.O.N.); dalmasri@kau.edu.sa (D.M.A.)
[7] Faculty of Dentistry, British University, El Sherouk City, Suez Desert Road, Cairo 11837, Egypt; shaimaag1973@gmail.com
[8] Department of Natural Products and Alternative Medicine, Faculty of Pharmacy, King Abdulaziz University, Jeddah 21589, Saudi Arabia; gahussein@kau.edu.sa

* Correspondence: Email: sabrin.ibrahim@bmc.edu.sa; Tel: (+966)581183034.

Table S1. Physical and spectral data of newly reported naturally occurring thiophenes from 2015 to 2021.

Compound name	Spectroscopic data	Reference
Foetithiophene C (**3**)		[25]
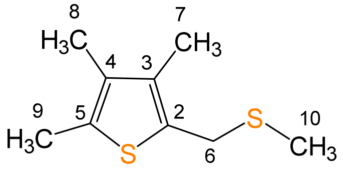<br>Foetithiophene C (**3**)<br>Yellow oil; UV λ_{max} (MeOH) (log ε): 248 (2.79) nm; IR (KBr) ν_{max}: (CH_2Cl_2) 2916, 2851, 1503, 1466, 1250, and 1210 cm^{-1}; ^1H NMR ($CDCl_3$, 500 MHz): 3.75 (2H, s, 6-CH_2), 2.03 (3H, s, 7-CH_3), 1.97 (3H, s, 8-CH_3), 2.28 (3H, s, 9-CH_3), 2.07 (3H, s, 10-CH_3); ^{13}C NMR ($CDCl_3$, 125 MHz): 129.5 (C-2), 134.8 (C-3), 133.0 (C-4), 130.2 (C-5), 30.6 (C-6), 12.9 (C-7), 12.4 (C-8), 13.2 (C-9), 15.2 (C-10); HREIMS $[M]^+$, *m/z* 186.0533, calcd 186.0537.		
Foetithiophene D (**4**)		[25]
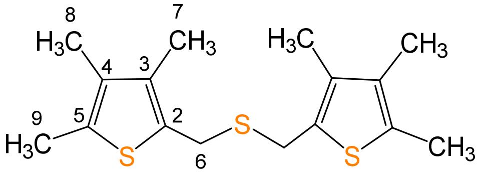<br>Foetithiophene D (**4**)<br>Yellow oil; UV λ_{max} (MeOH) (log ε): 248 (3.17) nm; IR (KBr) ν_{max} (CH_2Cl_2): 2922, 2853, 1652, 1559, 1458, and 1252 cm^{-1}; ^1H NMR ($CDCl_3$, 500 MHz): 3.84 (2H, s, 6-CH_2), 2.04 (3H, s, 7-CH_3), 1.98 (3H, s, 8-CH_3), 2.28 (3H, s, 9-CH_3); ^{13}C NMR ($CDCl_3$, 125 MHz): 127.9 (C-2), 136.3 (C-3), 133.6 (C-4), 131.5 (C-5), 36.5 (C-6), 13.3 (C-7), 12.8 (C-8), 13.5; HREIMS ($[M]^+$, *m/z* 310.0873, calcd 310.0884).		
Foetithiophene E (**5**)		[25]

<table>
<tr><td colspan="2">
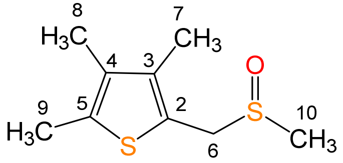

Foetithiophene E (**5**)
Needle crystals; MP. 91–93 °C; UV λ_{max} (MeOH) (log ε): 248 (3.94) nm; IR (KBr) ν_{max} (CH_2Cl_2): 2914, 2854, 1644, 1413, 1003, and 950 cm^{-1}; ^1H NMR ($CDCl_3$, 500 MHz): 4.18 (1H, d, J = 13.8 Hz, H-6A), 4.00 (1H, d, J = 13.8 Hz, H-6B), 2.08 (3H, s, 7-CH_3), 1.99 (3H, s, 8-CH_3), 2.29 (3H, s, 9-CH_3), 2.48 (3H, s, 10-CH_3); ^{13}C NMR ($CDCl_3$, 125 MHz): 119.3 (C-2), 137.9 (C-3), 134.0 (C-4), 132.8 (C-5), 53.5 (C-6), 13.7 (C-7), 12.8 (C-8), 13.4 (C-9), 37.6 (C-10); HRESIMS ($[M+H]^+$; m/z 203.0554, calcd 203.0558).
</td></tr>
<tr><td>Foetithiophene F (6)</td><td>[25]</td></tr>
<tr><td colspan="2">
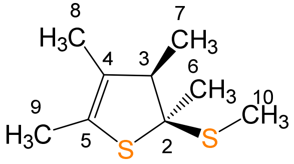

Foetithiophene F (**6**)
Yellow oil; $[\alpha]^{25}_D$ +76.9 (c 0.39, CH_2Cl_2); UV λ_{max} (MeOH) (log ε): 248 (2.29) nm; IR (KBr) ν_{max}: 2920, 1684, 1653, 1559, 1436, 1202, 1132, and 1029 cm^{-1}; ^1H NMR ($CDCl_3$, 500 MHz): 3.36 (1H, q, J = 7.5 Hz, H-3), 1.68 (3H, s, 6-CH_3), 1.14 (3H, d, J = 7.5 Hz, H-7), 1.71 (3H, s, 8-CH_3), 2.08 (3H, s, 9-CH_3), 2.13 (3H, s, 10-CH_3); ^{13}C NMR ($CDCl_3$, 125 MHz): 71.8 (C-2), 52.3(C-3), 147.05 (C-4), 135.84 (C-5), 17.3 (C-6), 12.9 (C-7), 14.1 (C-8), 12.2 (C-9), 12.7 (C-10); HREIMS $[M]^+$, m/z 188.0690, calcd 188.0693.
</td></tr>
<tr><td>5-Propinyl-thiophene-2-carboxylic acid (7)</td><td>[26]</td></tr>
<tr><td colspan="2">
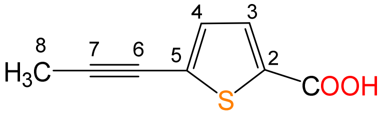

5-Propinyl-thiophene-2-carboxylic acid (**7**)
Colorless crystals; MP. 7982 °C; ^1H NMR (500 MHz, CD_3OD): 7.59 (1H, d, J = 3.8 Hz, H-3), 7.08 (1H, d, J = 3.8 Hz, H-4), 2.08 (3H, s, H-8). ^{13}C NMR (125 MHz, CD_3OD): 164.7 (COOH-2), 135.4 (C-2), 134.2 (C-3), 132.1 (C-4), 132.6 (C-5), 73.2 (C-6), 93.8 (C-7), 4.1 (C-8); HRESIMS: m/z 165.0013 $[M-H]^-$ (calcd for $C_8H_6O_2S$ 165.0010).
</td></tr>
<tr><td>3-Hydroxy-5-propinyl-2-acetyl-thiophene (8)</td><td>[26]</td></tr>
</table>

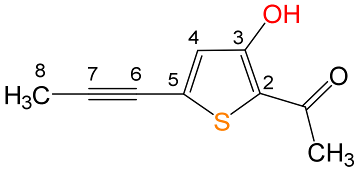<br>3-Hydroxy-5-propinyl-2-acetyl-thiophene (**8**)<br>White crystals; MP. 295296 °C; ^{1}H NMR (500 MHz, $CDCl_3$): 6.72 (1H, s, H-4), 2.39 (3H, s, $COCH_3$-2), 2.11 (3H, s, H-8); ^{13}C NMR (125 MHz, $CDCl_3$): 194.1 (C=O-2), 165.3 (C-3), 130.5 (C-5), 122.6 (C-4), 113.9 (C-2), 95.2 (C-7), 73.0 (C-6), 27.6 (CH_3-2), 4.8 (C-8); HRESIMS: *m/z* 181.0329 $[M+H]^+$ (calcd for $C_9H_8O_2S$ 181.0323).	
Echinothiophene A (**15**)	[19]
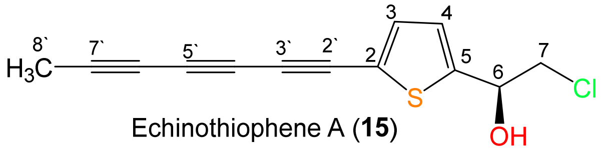<br>Echinothiophene A (**15**)<br>Yellow oil; $[\alpha]_D^{25}$ +16.4 (*c* 0.11, MeOH); ECD (CH_3CN) λ_{max} ($\Delta\varepsilon$) 259 (-6.0) nm; ^{1}H NMR (600 MHz, $CDCl_3$): 7.23 (1H, d, *J* = 3.6 Hz, H-3), 6.89 (1H, d, *J* = 3.7 Hz, H-4), 5.11 (1H, dd, *J* = 8.0, 3.3 Hz, H-6), 3.78 (1H, dd, *J* = 11.3, 3.6 Hz, H-7A), 3.69 (1H, dd, *J* = 11.3, 8.0 Hz, H-7B), 2.02 (3H, s, H-8\`); $^{13}$C NMR (150 MHz, $CDCl_3$): 121.6 (C-2), 135.2 (C-3), 124.5 (C-4), 147.0 (C-5), 70.3 (C-6), 50.1 (C-7), 67.8 (C-2\`), 79.4 (C-3\`), 58.5 (C-4\`), 64.9 (C-5\`), 70.1 (C-6\`), 79.7 (C-7\`), 4.7 (C-8\`); HRESIMS m/z, 270.9963 $[M+Na]^+$, calcd for $C_{13}H_9ONaSCl$, 270.9960.	
Echinothiophene B (**16**)	[19]
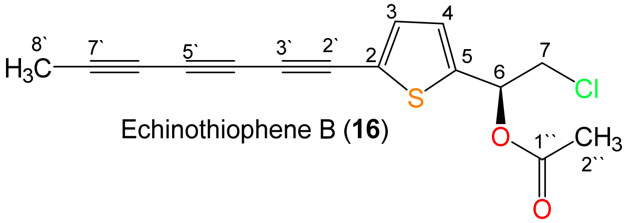<br>Echinothiophene B (**16**)<br>Yellow oil; $[\alpha]_D^{25}$ +18.2 (*c* 0.12, MeOH); ECD (CH_3CN) λ_{max} ($\Delta\varepsilon$) 261 (-5.9) nm; ^{1}H NMR (600 MHz, $CDCl_3$): 7.25 (1H, d, *J* = 3.5 Hz, H-3), 6.99 (1H, d, *J* = 3.6 Hz, H-4), 6.17 (1H, dd, *J* = 7.2, 4.8 Hz, H-6), 3.82 (1H, dd, *J* = 11.5, 7.3 Hz, H-7A), 3.78 (1H, dd, *J* = 11.5, 5.0 Hz, H-7B), 2.02 (3H, s, H-8\`), 2.16 (3H, s, H-2\`\`); $^{13}$C NMR (150 MHz, $CDCl_3$): 122.4 (C-2), 134.9 (C-3), 126.6 (C-4), 142.8 (C-5), 70.2 (C-6), 45.9 (C-7), 67.5 (C-2\`), 79.7 (C-3\`), 58.5 (C-4\`), 64.9 (C-5\`), 70.2 (C-6\`), 79.8 (C-7\`), 4.7 (C-8\`), 169.5 (C-1\`\`), 20.8 (C-2\`\`); HRESIMS m/z, 313.0066 $[M+Na]^+$, calcd for $C_{15}H_{11}O_2NaSCl$, 313.0066.	

Echinothiophene C (**17**)	[19]
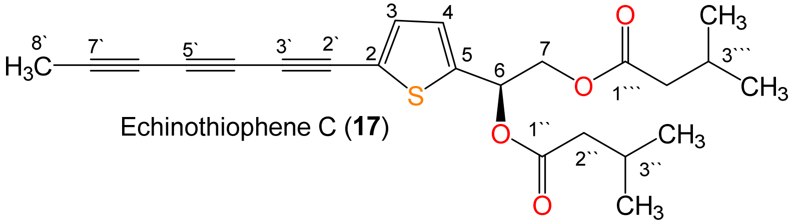<br>Echinothiophene C (**17**)<br>Yellow oil; $[\alpha]_D^{25}$ +32.2 (*c* 0.13, MeOH); ECD (CH_3CN) λ_{max} (Δε) 260 (-5.7) nm; ^{1}H NMR (600 MHz, $CDCl_3$): 7.24 (1H, d, *J* = 3.6 Hz, H-3), 6.97 (1H, d, *J* = 3.6 Hz, H-4), 6.25 (1H, dd, *J* = 6.8, 4.0 Hz, H-6), 4.45 (1H, dd, *J* = 11.7, 3.8 Hz, H-7A), 4.33 (1H, dd, *J* = 11.8, 7.1 Hz, H-7B), 2.05 (3H, s, H-8`), 2.24 (2H, d, *J* = 7.9 Hz, H-2``), 2.11 (1H, m, H-3``), 0.96 (3H, d, *J* = 6.6 Hz, H-4``), 0.96 (3H, d, *J* = 6.6 Hz, H-5``), 2.22 (2H, d, *J* = 7.9 Hz, H-2```), 2.11 (1H, m, H-3```), 0.96 (3H, d, *J* = 6.6 Hz, H-4```), 0.96 (3H, d, *J* = 6.6 Hz, H-5```); ^{13}C NMR (150 MHz, $CDCl_3$): 122.2 (C-2), 134.9 (C-3), 126.4 (C-4), 142.6 (C-5), 68.5 (C-6), 65.0 (C-7), 67.6 (C-2`), 79.5 (C-3`), 58.5 (C-4`), 64.9 (C-5`), 70.1 (C-6`), 79.7 (C-7`), 4.7 (C-8`), 171.7 (C-1``), 43.2 (C-2``); 25.7 (C-3``), 22.3 (C-4``), 22.3 (C-5``), 172.4 (C-1```), 43.1 (C-2```); 25.6 (C-3```), 22.3 (C-4```), 22.3 (C-5```); HRESIMS m/z, 421.1446 $[M+Na]^+$, calcd for $C_{23}H_{26}O_4NaS$, 421.1449.	
3``*R*-Pluthiophenol (**23**)	[35]
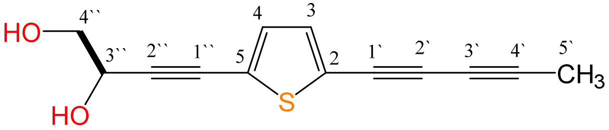<br>3``*R*-Pluthiophenol (**23**)<br>Yellow oil; $[\alpha]_D^{25}$ + 11.4 (*c* 0.04, MeOH); UV (MeOH) λ_{max} (log *ε*): 209 (4.45), 235 (3.92), 246 (4.07), 319 (4.49), 340 (4.47) nm; ; IR (KBr)ν_{max}: 3312, 3105, 2955, 2919, 2871, 2467, 2222, 2148, 1448, 1077, 1022, 804 cm^{-1}; ^{1}H NMR (500 MHz, CD_3OD): 7.15 (1H, d, *J* = 4.0 Hz, H-3), 7.08 (1H, d, *J* = 4.0 Hz, H-4), 2.02 (3H, s, H-5`); 4.55 (1H, dd, *J* = 7.0, 5.0 Hz, H-3``), 3.68 (1H, dd, *J* = 11.5, 5.0 Hz, H-4``A), 3.64 (1H, dd, *J* = 11.5, 7.0 Hz, H-4``B); $^{13}$C NMR (125 MHz, $CD_3OD$): 124.6 (C-2), 134.9 (C-3), 133.3 (C-4), 125.9 (C-5), 66.8 (C-1`), 80.1 (C-2`), 64.6 (C-3`), 84.5 (C-4`), 4.2 (C-5`), 78.1 (C-1``), 94.5 (C-2``), 64.6 (C-3``), 66.9 (C-4``); ESI-Q-Orbitrap MS: Positive-ion mode *m/z* 231.04726 $[M+H]^+$ (calcd for $C_{13}H_{11}O_2S$, 231.04743).	
3``*R*-Pluthiophenol-4``-acetate (**24**)	[35]

<table>
<tr><td colspan="2">
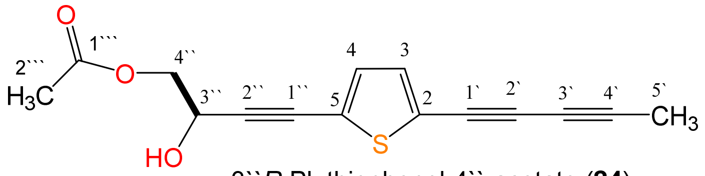

3``*R*-Pluthiophenol-4``-acetate (**24**)

Yellow oil; $[\alpha]_D^{25}$ + 7.3 (c = 0.06, MeOH); UV (MeOH) λ_{max} (log ε): 208 (4.54), 235 (4.01), 246 (4.16), 319 (4.58), 340 (4.56) nm; IR (KBr)ν_{max}: 3099, 2977, 2233, 1745, 1520, 1448, 1381, 1326, 1229, 1106, 1046, 807 cm^{-1}; ^{1}H NMR (500 MHz, CD_3OD): 7.17 (1H, d, *J* = 4.0 Hz, H-3), 7.10 (1H, d, *J* = 4.0 Hz, H-4), 2.03 (3H, s, H-5`); 4.76 (1H, dd, *J* = 6.5, 5.0 Hz, H-3``), 4.21 (1H, dd, *J* = 11.0, 5.0 Hz, H-4``A), 4.19 (1H, dd, *J* = 11.0, 6.5 Hz, H-4``B), 2.08 (3H, s, H-2```); ^{13}C NMR (125 MHz, CD_3OD): 125.0 (C-2), 135.0 (C-3), 133.6 (C-4), 125.5 (C-5), 67.0 (C-1`), 80.1 (C-2`), 64.7 (C-3`), 84.6 (C-4`), 4.1 (C-5`), 78.5 (C-1``), 93.3 (C-2``), 61.8 (C-3``), 68.1 (C-4``), 172.5 (C-1```), 20.7 (C-2```); ESI-Q-Orbitrap MS: Positive-ion mode *m*/*z* 273.05781 $[M+H]^+$ (calcd for $C_{15}H13O_3S$, 273.05799).
</td></tr>
<tr><td>3``-Ethoxy-3``S-pluthiophenol (25)</td><td>[35]</td></tr>
<tr><td colspan="2">
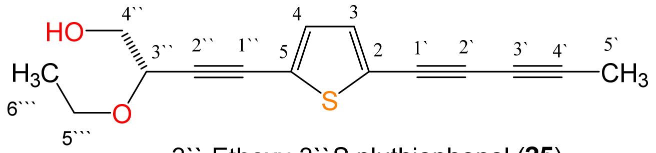

3``-Ethoxy-3``*S*-pluthiophenol (**25**)

Yellow oil; $[\alpha]_D^{25}$ – 16.7 (c = 0.06, MeOH); UV (MeOH) λ_{max} (log ε): 208 (4.47), 235 (3.99), 246 (4.11), 319 (4.50), 340 (4.47) nm; IR (KBr)ν_{max}: 3439, 3097, 2975, 2931, 2876, 2231, 1447, 1376, 1327, 1118, 807 cm^{-1}; ^{1}H NMR (500 MHz, CD_3OD): 7.16 (1H, d, *J* = 4.0 Hz, H-3), 7.10 (1H, d, *J* = 4.0 Hz, H-4), 2.03 (3H, s, H-5`); 4.34 (1H, t, *J* = 5.5 Hz, H-3``), 3.69 (1H, d, *J* = 5.5 Hz, H-4``), 3.83 (1H, dq, *J* = 9.0, 7.0 Hz, H-5``A), 3.55 (1H, dq, *J* = 9.0, 7.0 Hz, H-5``B), 1.24 (3H, t, *J* = 7.0 Hz, H-6``); $^{13}C$ NMR (125 MHz, $CD_3OD$): 124.8 (C-2), 135.0 (C-3), 133.6 (C-4), 125.6 (C-5), 66.7 (C-1`), 80.2 (C-2`), 64.5 (C-3`), 84.6 (C-4`), 4.1 (C-5`), 79.5 (C-1``), 92.5 (C-2``), 72.7 (C-3``), 65.6 (C-4``), 66.1 (C-5``), 15.5 (C-6``); ESI-Q-Orbitrap MS: Positive-ion mode *m*/*z* 259.07875 $[M+H]^+$ (calcd for $C_{15}H_{15}O_2S$, 259.07873).
</td></tr>
<tr><td>3``-Ethoxy-3``S-pluthiophenol-4``-acetate (26)</td><td>[35]</td></tr>
</table>

<table>
<tr><td colspan="2">

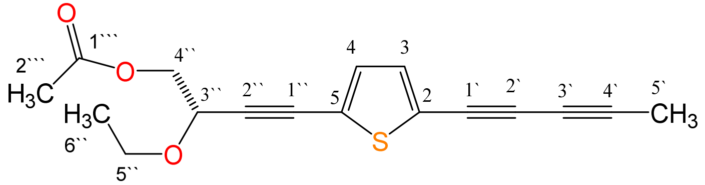

3``-Ethoxy-3``*S*-pluthiophenol-4``-acetate (**26**)

Yellow oil; $[\alpha]_D^{25}$ – 8.9 (c = 0.04, MeOH); UV (MeOH) λ_{max} (log ε): 208 (4.72), 235 (4.18), 246 (4.34), 319 (4.76), 340 (4.73) nm; IR (KBr)ν_{max}: 3098, 2976, 2228, 1745, 1445, 1377, 1330, 1231, 1105, 1048, 808 cm^{-1}; ^{1}H NMR (500 MHz, CD$_3$OD): 7.17 (1H, d, *J* = 4.0 Hz, H-3), 7.12 (1H, d, *J* = 4.0 Hz, H-4), 2.03 (3H, s, H-5`); 4.57 (1H, dd, *J* = 6.0, 4.5 Hz, H-3``), 4.26 (1H, dd, *J* = 11.5, 6.0 Hz, H-4``A), 4.23 (1H, dd, *J* = 11.5, 4.5 Hz, H-4``B), 3.81 (1H, dq, *J* = 9.0, 7.0 Hz, H-5``A), 3.55 (1H, dq, *J* = 9.0, 7.0 Hz, H-5``B), 1.24 (3H, t, *J* = 7.0 Hz, H-6``), 2.07 (3H, s, H-2```); ^{13}C NMR (125 MHz, CD$_3$OD): 125.2 (C-2), 135.1 (C-3), 133.9 (C-4), 125.2 (C-5), 66.6 (C-1`), 80.3 (C-2`), 64.5 (C-3`), 84.7 (C-4`), 4.1 (C-5`), 79.9 (C-1``), 91.2 (C-2``), 69.5 (C-3``), 66.5 (C-4``), 66.1 (C-5``), 15.4 (C-6``), 172.4 (C-1```), 20.7 (C-2```); ESI-Q-Orbitrap MS: Positive-ion mode *m*/*z* 301.08969 [M+H]$^+$ (calcd for $C_{17}H_{17}O_3S$, 301.08929).

</td></tr>
<tr><td>Rupestriene B (27)</td><td>[36]</td></tr>
<tr><td colspan="2">

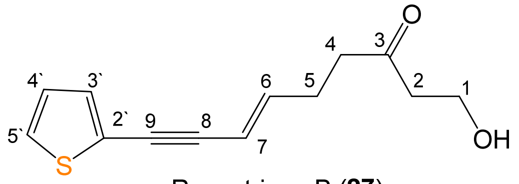

Rupestriene B (**27**)

Brown oil; IR (KBr) ν_{max}: 3399, 2932, 1708, 1046, 955, 705 cm^{-1}; ^{1}H NMR (CD$_3$OD, 500 MHz): 3.80 (2H, t, *J* = 6.1, Hz, H-1), 2.65 (2H, t, *J* = 6.1 Hz, H-2), 2.64 (2H, t, *J* = 7.5 Hz, H-4), 2.40 (2H, tdd, *J* = 7.5, 7.0, 2.0 Hz, H-5), 6.18 (1H, dt, *J* = 15.9, 7.0 Hz, H-6), 5.75 (1H, dt, *J* = 15.9, 7.0 Hz, H-7), 7.13 (1H, d, *J* = 3.5 Hz, H-3`), 6.97 (1H, dd, *J* = 5.1, 3.5 Hz, H-4`), 7.34 (1H, d, *J* = 5.1 Hz, H-5`); $^{13}$C NMR (CD$_3$OD, 500 MHz): 58.2 (C-1), 46.1 (C-2), 210.7 (C-3), 42.7 (C-4), 27.9 (C-5), 144.5 (C-6), 111.1 (C-7), 92.5 (C-8), 82.5 (C-9), 124.7 (C-2`), 132.5 (C-3`), 128.1 (C-4`), 128.1 (C-5`); positive HRESIMS: *m*/*z* 257.0612 [M+Na]$^+$ (calcd for $C_{13}H^{14}SO_2Na$, 257.0607).

</td></tr>
<tr><td>Rupestriene C (28)</td><td>[36]</td></tr>
</table>

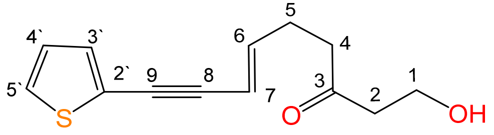 Rupestriene C (**28**) Brown oil; IR (KBr) ν_{max}: 3306, 2959, 1707, 1115, 980, 755 cm^{-1}; ^{1}H NMR (CD_3OD, 500 MHz): 3.80 (2H, t, *J* = 6.1, Hz, H-1), 2.65 (2H, t, *J* = 6.1 Hz, H-2), 2.64 (2H, t, *J* = 7.2 Hz, H-4), 2.60 (2H, t, *J* = 7.2, Hz, H-5), 6.18 (1H, dt, *J* = 10.7, 7.2 Hz, H-6), 5.75 (1H, d, *J* = 10.7 Hz, H-7), 7.18 (1H, d, *J* = 3.6 Hz, H-3`), 7.00 (1H, dd, *J* = 5.2, 3.6 Hz, H-4`), 7.38 (1H, d, *J* = 5.2 Hz, H-5`); $^{13}$C NMR ($CD_3OD$, 500 MHz): 58.2 (C-1), 46.1 (C-2), 211.0 (C-3), 42.9 (C-4), 25.6 (C-5), 143.4 (C-6), 110.5 (C-7), 90.4 (C-8), 88.1 (C-9), 124.3 (C-2`), 132.7 (C-3`), 128.2 (C-4`), 128.4 (C-5`); positive HRESIMS: *m/z* 257.0612 [M+Na]$^{+}$ (calcd for $C_{13}H_{14}SO_2Na$, 257.0607).	
Atracthioenyneside A (**29**)	[37]
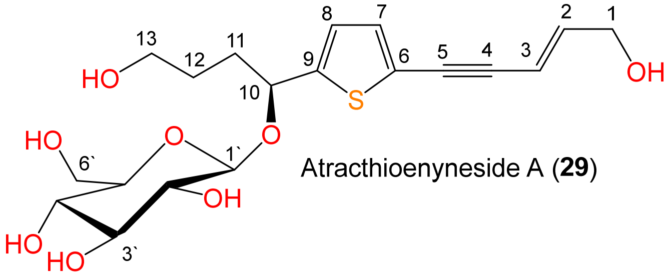 Atracthioenyneside A (**29**) Pale brown amorphous powder; $[\alpha]_D^{20}$ -106.8 (*c* 0.08, MeOH); UV (MeOH) λ_{max} (log ε): 298 (4.19), 314 (4.11) nm; ECD (MeOH) $\Delta\varepsilon_{217\,nm}$ -0.94, $\Delta\varepsilon_{294\,nm}$ -2.30; IR (KBr)ν_{max}: 3379, 2931, 2190, 1703, 1078 cm^{-1}; ^{1}H NMR (500 MHz, DMSO-*d*$_6$): 4.06 (2H, dd, *J* = 4.5, 2.0 Hz, H-1), 6.34 (1H, dt, *J* = 16.0, 4.5 Hz, H-2), 5.95 (1H, dt, *J* = 16.0, 2.0 Hz, H-3), 7.16 (1H, d, *J* = 3.5 Hz, H-7), 7.02 (1H, d, *J* = 3.5 Hz, H-8), 5.04 (1H, d, *J* = 6.5 Hz, H-10), 1.81-1.83 (1H, m, H-11A), 1.70-1.71 (1H, m, H-11B), 1.44-1.46 (1H, m, H-12A), 1.35-1.37 (1H, m, H-12B), 3.37 (2H, m, H-13), 3.99 1H, d, *J* = 7.5 Hz, H-1`), 2.98 (1H, m, H-2`), 2.97 (1H, m, H-3`), 3.00 (1H, m, H-4`), 3.02 (1H, m, H-5`), 3.67 (1H, dd, *J* = 11.5, 5.5 Hz, H-6`A), 3.43 (1H, dd, *J* = 11.5, 5.5 Hz, H-6`B),; $^{13}$C NMR (150 MHz, DMSO-*d*$_6$): 60.9 (C-1), 145.3 (C-2), 107.3 (C-3), 92.2 (C-4), 82.6 (C-5), 121.9 (C-6), 131.6 (C-7), 126.6 (C-8), 147.5 (C-9), 73.3 (C-10), 34.5 (C-11), 28.6 (C-12), 60.4 (C-13), 99.5 (C-1`), 73.4 (C-2`), 77.1 (C-3`), 70.3 (C-4`), 76.8 (C-5`), 61.3 (C-6`); HRESIMS: *m/z* 459.1339 [M+COOH]$^{-}$ (calcd for $C_{20}H_{27}O_{10}S$, 459.1325)	
Atracthioenyneside A (**30**)	[37]

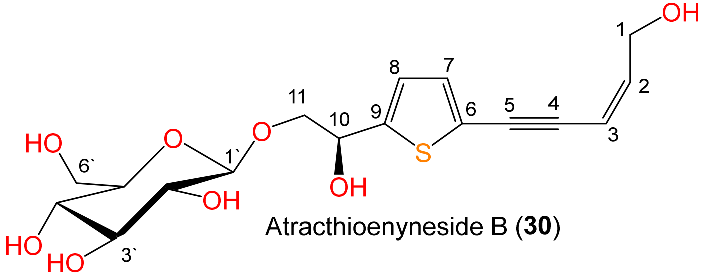

Atracthioenyneside B (**30**)

Pale brown amorphous powder; $[\alpha]_D^{20}$ -66.2 (*c* 0.08, MeOH); UV (MeOH) λ_{max} (log ε): 301 (4.10), 316 (4.03) nm; ECD (MeOH) $\Delta\varepsilon_{220\,nm}$ -2.08, $\Delta\varepsilon_{295\,nm}$ -0.62; IR (KBr)ν_{max}: 3359, 2881, 2192, 1596, 1078, 1044 $^{cm-1}$; ^{1}H NMR (500 MHz, DMSO-d_6): ^{1}H NMR (500 MHz, DMSO-d_6): 4.61 (2H, m, H-1), 5.98 (1H, dd, *J* = 11.0, 8.5 Hz, H-2), 5.88 (1H, brd, *J* = 11.0 Hz, H-3), 7.18 (1H, d, *J* = 3.5 Hz, H-7), 6.91 (1H, d, *J* = 3.5 Hz, H-8), 4.62 (1H, m, H-10), 3.71 (1H, dd, *J* = 11.0, 7.0 Hz, H-11A), 3.51 (1H, dd, *J* = 11.0, 3.5 Hz, H-11B), 4.18 1H, d, *J* = 7.5 Hz, H-1`), 2.98-3.00 (1H, d, *J* = 7.5 Hz, H-2`), 3.04 (1H, m, H-3`), 3.05 (1H, m, H-4`), 3.13-3.15 (1H, m, H-5`), 3.64 (1H, dd, *J* = 11.5, 5.5 Hz, H-6`A), 3.41-3.43 (1H, m, H-6`B); $^{13}$C NMR (150 MHz, DMSO-$d_6$): 58.4 (C-1), 143.6 (C-2), 109.4 (C-3), 89.3 (C-4), 88.0 (C-5), 120.4 (C-6), 132.3 (C-7), 124.3 (C-8), 149.6 (C-9), 68.4 (C-10), 72.7 (C-11), 103.2 (C-1`), 73.5 (C-2`), 77.0 (C-3`), 70.0 (C-4`), 76.5 (C-5`), 61.0 (C-6`); HRESIMS: *m/z* 431.1026 $[M+COOH]^-$ (calcd for $C_{18}H_{23}O_{10}S$, 431.1012).

(*Z*)-6-(5-(prop-1-yn-1-yl)thiophen-2-yl)hex-3-en-5-yne-1,2-diol (**31**)	[38]

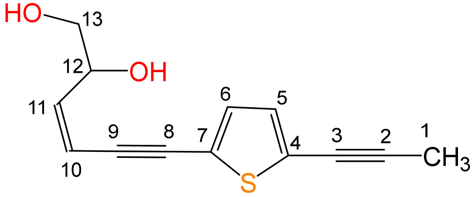

(*Z*)-6-(5-(prop-1-yn-1-yl)thiophen-2-yl)hex-3-en-5-yne-1,2-diol (**31**)

Yellow powder; UV (MeOH) λ_{max} (log ε) 338 (3.85) nm; ^{1}H NMR ($CDCl_3$, 600 MHz); 2.08 (3H, s, H-1), 6.96 (1H, d, *J* = 3.8 Hz, H-5), 7.02 (1H, d, *J* = 3.8 Hz, H-6), 5.85 (1H, d, *J* = 11.0 Hz, H-10), 5.98 (1H, dd, *J* = 11.0, 8.0 Hz, H-11), 4.81 (1H, ddd, *J* = 8.0, 7.4, 3.3 Hz, H-12), 3.77 (1H, dd, *J* = 11.1, 3.3 Hz, H-13A), 3.60 (1H, dd, *J* = 11.1, 7.4 Hz, H-13B); ^{13}C NMR (150 MHz, $CDCl_3$): 5.0 (C-1), 92.0 (C-2), 72.8 (C-3), 126.5 (C-4), 131.1 (C-5), 132.0 (C-6), 122.7 (C-7), 88.7 (C-8), 89.0 (C-9), 111.7 (C-10), 141.2 (C-11), 71.2 (C-12), 65.8 (C-13); (+)-ESIMS: *m/z* 255.0 $[M+Na]^+$; (+)-HRESIMS: *m/z* 255.0446 $[M+Na]^+$ (calcd for $C_{13}H_{12}O_2SNa$, 255.0450).

5-(4-Hydroxy-3-methoxy-1-butyny)-2,2`-bithiophene (32)	[28]

<table>
<tr><td colspan="2">
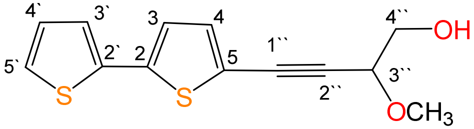

5-(4-Hydroxy-3-methoxy-1-butyny)-2,2`-bithiophene (**32**)

Yellow oil; $[\alpha]^{25}_{D}$: +0.78 (*c* 0.23, $CHCl_3$); IR (KBr) ν_{max}: 3431, 2214, 802, 696 cm^{-1}; UV λ_{max} (MeOH) (log ε): 234 (3.80), 280 (4.52), 324 (3.45) nm; ^{1}H NMR ($CDCl_3$, 400 MHz): 7.13 (1H, d, *J* = 3.8 Hz, H-3), 7.00 (1H, d, *J* = 3.8 Hz, H-4), 7.17 (1H, d, *J* = 3.6 Hz, H-3`), 7.02 (1H, dd, *J* = 5.1, 3.6 Hz, H-4`), 7.24 (1H, d, *J* = 5.1 Hz, H-5`), 4.31 (1H, t, *J* = 5.7 Hz, H-3``), 3.82 (2H, d, *J* = 5.7 Hz, H-4``), 3.52 (3H, s, 3``-$OCH_3$); $^{13}C$ NMR ($CDCl_3$, 50 MHz): 139.5 (C-2), 133.8 (C-3), 128.2 (C-4), 122.3 (C-5), 136.5 (C-2`), 124.6 (C-3`), 123.6 (C-4`), 125.4 (C-5`), 90.0 (C-1``), 80.7 (C-2``), 73.0 (C-3``), 65.3 (C-4``), 57.3 (3``-OCH_3): HRESIMS: *m*/*z* 262.9758 $[M-2H]^{+}$, calcd for $C_{13}H_{12}O_2S_2$.
</td></tr>
<tr><td>Thiotagetin B: [(Z)-1``-([2,2`-bithiophen]-5-yl)-8``-chloro-6``,11``-dimethylundeca-6``,10``-dien-2``-yn-9``-one] (46)</td><td>[44]</td></tr>
<tr><td colspan="2">
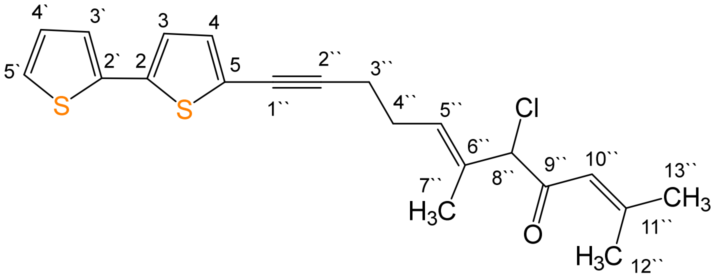

Thiotagetin B: [(*Z*)-1``-([2,2`-bithiophen]-5-yl)-8``-chloro-6``,11``-dimethylundeca-6``,10``-dien-2``-yn-9``-one] (**46**)

Yellow amorphous powder, $[\alpha]_{D}^{20}$ + 19.3 (*c* 0.06, $CHCl_3$); UV (MeOH) λ_{max}: 216, 268, 370 nm; IR (KBr)ν_{max}: 3425, 2879, 2200, 1683, 1618, 805 cm^{-1}; ^{1}H NMR (600 MHz, $CDCl_3$): 7.00 (1H, d, *J* = 4.2 Hz, H-3), 7.05 (1H, d, *J* = 4.2 Hz, H-4), 7.22 (1H, dd, *J* = 4.8, 1.2 Hz, H-3`), 7.01 (1H, dd, *J* = 4.8, 3.6 Hz, H-4`), 7.16 (1H, dd, *J* = 3.6, 1.2 Hz, H-3`), 2.34 (1H, m, H-3``A), 1.62 (1H, m, H-3``B), 2.36 (1H, m, H-4``A), 2.33 (1H, m, H-4``B), 5.70 (1H, brt, *J* = 5.8 Hz, H-5``), 1.64 (3H, d, *J* = 1.2 Hz, H-7``), 3.15 (1H, s, H-8``), 6.21 (1H, brs, H-10``), 2.17 (3H, d, *J* = 1.2 Hz, H-12``), 1.95 (3H, d, *J* = 1.2 Hz, H-13``); ^{13}C NMR (150 MHz, $CDCl_3$): 138.6 (C-2), 123.3 (C-3), 132.4 (C-4), 122.5 (C-5), 137.5 (C-2`), 124.7 (C-3`), 127.9 (C-4`), 123.5 (C-5`), 75.2 (C-
</td></tr>
</table>

1``), 89.0 (C-2``), 27.0 (C-3``), 22.4 (C-4``), 123.5 (C-5``), 129.7 (C-6``), 22.5 (C-7``), 61.4 (C-8``), 198.7 (C-9``), 125.0 (C-10``), 159.8 (C-11``), 21.2 (C-12``), 28.1 (C-13``); HRESIMS *m/z* 389.0807 (calcd for 389.0801 $[M+H]^+$, $C_{21}H_{22}{}^{35}ClOS_2$), 391.0954 (calcd for 391.0957 $[M+H]^+$, $C_{21}H_{22}{}^{37}ClOS_2$).	
6-Methoxy-arctinol-b (**48**)	[34]
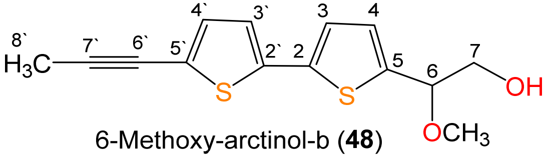 6-Methoxy-arctinol-b (**48**) Yellow amorphous powder; ^{1}H NMR (500 MHz, $CDCl_3$): 7.05 (1H, d, *J* = 3.5 Hz, H-3), 7.02 (1H, d, *J* = 3.5 Hz, H-4`), 6.99 (1H, d, *J* = 3.5 Hz, H-3`), 6.94 (1H, d, *J* = 3.5 Hz, H-4), 4.51 (1H, dd, *J* = 7.0, 4.0 Hz, H-6), 3.82 (1H, dd, *J* = 12.0, 7.0 Hz, H-7a), 3.76 (1H, dd, *J* = 12.0, 4.0 Hz, H-7b), 3.40 (3H, s, 6-OCH_3), 2.11 (3H, s, 8`-$CH_3$); $^{13}$C NMR (125 MHz, $CDCl_3$): 140.8 (C-5), 137.2 (C-2, C-20), 131.7 (C-40), 126.9 (C-4), 123.3 (C-50, C-3), 123.2 (C-30), 91.3 (C-70), 79.9 (C-6), 72.8 (C-60), 67.0 (C-7), 56.9 ($OCH_3$), 4.76 (C-8`); HRESIMS *m/z* 301.0336 $[M+Na]^+$, calcd for $C_{14}H_{14}S_2NaO_2$ 301.0327.	
2,2-Dimethyl-4-[5`-(prop-1-ynyl)-2,2`-bithiophen-5-yl]-1,3-dioxolane (**56**)	[45]
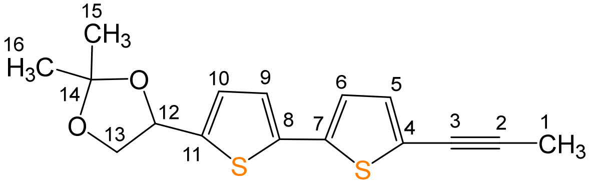 2,2-Dimethyl-4-[5`-(prop-1-ynyl)-2,2`-bithiophen-5-yl]-1,3-dioxolane (**56**) White powder.; $[\alpha]_D^{20}$ -20 (*c* 0.5, $CHCl_3$); UV (MeOH) λ_{max}: 306, 290 nm; ^{1}H NMR (600 MHz, $CDCl_3$): 2.07 (3H, s, CH_3-3), 6.98 (1H, d, *J* = 3.8 Hz, H-5), 6.95 (1H, d, *J* = 3.8 Hz, H-6), 6.98 (1H, d, *J* = 3.8 Hz, H-9), 6.89 (1H, d, *J* = 3.5 Hz, H-10), 5.26 (1H, brt, *J* = 6.8 Hz, H-12), 4.30 (1H, dd, *J* = 8.4, 6.7 Hz, H-13A), 3.87 (1H, dd, *J* = 8.4, 6.7 Hz, H-13B), 1.53 (3H, s, CH_3-15), 1.45 (3H, s, CH_3-16); ^{13}HC NMR (150 MHz, $CDCl_3$): 4.6 (C-1), 91.1 (C-2), 72.7 (C-13), 121.1 (C-4), 131.5 (C-5), 123.1 (C-6), 137.4 (C-7), 138.8 (C-8), 123.5 (C-9), 125.6 (C-10), 141.9 (C-11), 73.9 (C-12), 71.3 (C-13), 110.1 (C-14), (C-1),26.4 (C-15), 25.7 (C-16); HRESIMS *m/z* $[M+Na]^+$ 327.0482 (calcd for $C_{16}H_{16}O_2S_2Na$, 327.0484).	
5`-(3,4-Dihydroxybut-1-yn-1-yl)-[2,2`-bithiophene]-5-carbaldehyde (**57**)	[27]

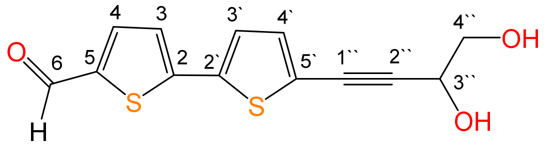<br>5`-(3,4-Dihydroxybut-1-yn-1-yl)-[2,2`-bithiophene]-5-carbaldehyde (**57**)<br>Primrose-yellow amorphous powder; IR (KBr)v_{max} 3282 (OH), 3087, 2963, 2927, 2217, 1650, 1455, 1436, 1233, 1086, 1057 cm^{-1}; ^{1}H NMR (500 MHz, CD_3OD): 7.41 (1H, d, *J* = 4.0 Hz, H-3), 7.85 (1H, d, *J* = 4.0 Hz, H-4), 9.85 (1H, s, H-6), 7.37 (1H, d, *J* = 3.8 Hz, H-3`), 7.22 (1H, d, *J* = 3.8 Hz, H-4`), 4.56 (1H, t, *J* = 6.5 Hz, H-3``), 3.67 (2H, m, H-4``); ^{13}C NMR (125 MHz, CD_3OD): 146.9 (C-2), 126.4 (C-3), 139.6 (C-4), 143.6 (C-5), 184.8 (C-6), 138.3 (C-2`), 127.3 (C-3`), 134.7 (C-4`), 125.4 (C-5`), 78.2 (C-1``), 95.8 (C-2``), 64.7 (C-3``), 67.0 (C-4``); EIMS (*m*/*z*): 278 $[M]^+$, 260 $[M\text{-}H_2O]^-$, 247, 231, 218, 190, 171, 158, 145, 95, 69; HRESIMS (*m*/*z*): 279.0145 $[M+H]^+$, Calc. for $[C_{13}H_{11}O_3S_2+H]^+$ 279.0149.	
4-Hydroxy-1-(5`-methyl-[2,2`-bithiophen]-5-yl)butan-1-one (**58**)	[27]
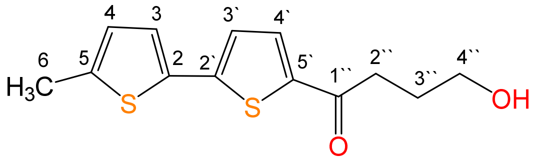<br>4-Hydroxy-1-(5`-methyl-[2,2`-bithiophen]-5-yl)butan-1-one (**58**)<br>Yellowish-white amorphous powder; IR (KBr,)v_{max} 3277 (OH), 3065, 2953, 2863, 1651, 1509, 1453, 1207, 1052, 1014, 807 cm^{-1}; ^{1}H NMR (500 MHz, DCl_3): 7.12 (1H, d, *J* = 3.6 Hz, H-3), 6.71 (1H, d, *J* = 3.6 Hz, H-4), 2.50 (3H, s, H-6), 7.09 (1H, d, *J* = 4.0 Hz, H-3`), 7.61 (1H, d, *J* = 4.0 Hz, H-4`), 3.04 (2H, t, *J* = 7.0 Hz, H-2``), 2.05-1.98 (2H, m H-3``), 3.75 (2H, t, *J* = 6.0 Hz, H-4``); $^{13}$C NMR (125 MHz, $CDCl_3$): 134.0 (C-2), 125.7 (C-3), 126.5 (C-4), 141.3 (C-5), 15.4 (C-6), 146.3 (C-2`), 123.4 (C-3`), 133.0 (C-4`), 141.7 (C-5`), 193.0 (C-1``), 35.5 (C-2``), 27.2 (C-3``), 62.3 (C-4``); EIMS (*m*/*z*): 266 $[M]^+$, 248 $[M\text{-}H_2O]^-$, 222, 207, 179, 149, 135, 91, 69; HRESIMS (*m*/*z*): 267.0506 $[M+H]^+$, calcd for $[C_{13}H_{14}O_2S_2+H]^+$ 267.0513.	
5`-(3,4-Dihydroxybut-1-yn-1-yl)-[2,2`-bithiophene]-5-carboxylic acid (**59**)	[27]

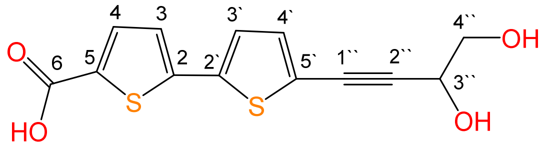<br>5`-(3,4-Dihydroxybut-1-yn-1-yl)-[2,2`-bithiophene]-5-carboxylic acid (**59**)<br>Primrose-yellow needles crystals, MP. 216–220 °C; IR (KBr,)ν_{max} 3426 (OH), 2311, 1630, 1552, 1434, 1379, 1320, 1149, 1105, 766 cm^{-1}; ^{1}H NMR (500 MHz, CD_3OD): 7.19-7.16 (1H, m, H-3), 7.19-7.16 (1H, m, H-4), 7.19-7.16 (1H, m, H-3`), 7.19-7.16 (1H, m, H-4`), 4.40 (1H, t, *J* = 6.0 Hz, H-3``); 3.47 (2H, d, *J* = 6.0 Hz, H-4``); ^{13}C NMR (125 MHz, CD_3OD): 138.6 (C-2), 123.7 (C-3), 133.4 (C-4), 135.5 (C-5), 164.3 (C-6), 128.5 (C-2`), 124.6 (C-3`), 120.4 (C-5`), 76.7 (C-1``), 96.1 (C-2``), 63.1 (C-3``), 65.5 (C-4``); ESI-MS (*m*/*z*): 293 [M-H]$^+$, 277, 249; HRESIMS (*m*/*z*): 292.9958 [M-H]$^+$, calcd for [$C_{13}H_9O_4S_2$-H]$^+$ 292.9942.	
Echinothiophene D (**61**)	[19]
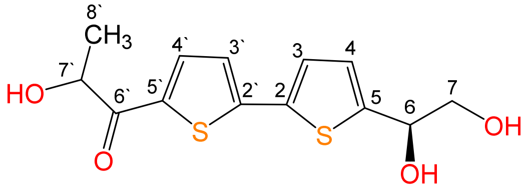<br>Echinothiophene D (**61**)<br>Yellow oil; $[\alpha]_D^{25}$ +3.8 (*c* 0.12, MeOH); ECD (CH_3CN) λ_{max} ($\Delta\varepsilon$) 260 (-5.9) nm; ^{1}H NMR (600 MHz, CD_3OD): 7.30 (1H, d, *J* = 3.6 Hz, H-3), 7.00 (1H, d, *J* = 3.5 Hz, H-4), 4.92 (1H, m, H-6), 3.71 (1H, m, H-7A), 3.70 (1H, m, H-7B), 7.28 (1H, d, *J* = 3.4 Hz, H-3`), 7.90 (1H, d, *J* = 3.2 Hz, H-4`), 4.93 (1H, m, H-7`), 1.45 (3H, s, H-8`); ^{13}C NMR (150 MHz, CD_3OD): 134.9 (C-2), 125.2 (C-3), 124.9 (C-4), 148.2 (C-5), 70.3 (C-6), 66.9 (C-7), 138.1 (C-2`), 123.8 (C-3`), 134.8 (C-4`), 146.5 (C-5`), 195.1 (C-6`), 70.4 (C-7`), 20.4 (C-8`); HRESIMS *m*/*z*, 299.0403 [M+H]$^+$, calcd for $C_{13}H_{15}O_4S_2$, 299.0412.	
Echinothiophene E (**62**)	[19]
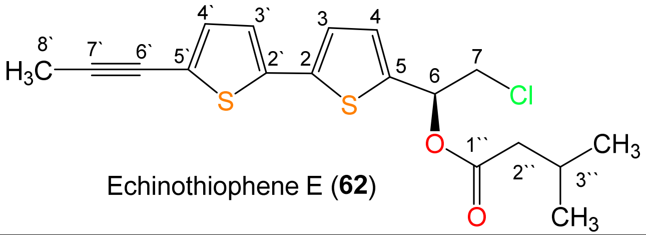<br>Echinothiophene E (**62**)	

Yellow oil; $[\alpha]_D^{25}$ +76.3 (c 0.11, MeOH); ECD (CH_3CN) λ_{max} ($\Delta\varepsilon$) 260 (-6.3) nm; ^{1}H NMR (600 MHz, $CDCl_3$): 7.04 (1H, d, *J* = 3.4 Hz, H-3), 7.01 (1H, d, *J* = 3.5 Hz, H-4), 6.21 (1H, dd, *J* = 7.5, 4.9 Hz, H-6), 3.88 (1H, dd, *J* = 11.5, 7.8 Hz, H-7A), 3.83 (1H, dd, *J* = 11.6, 4.8 Hz, H-7B), 6.99 (1H, d, *J* = 3.5 Hz, H-3`), 7.02 (1H, d, *J* = 3.5 Hz, H-4`), 2.11 (3H, s, H-8`), 2.30 (2H, d, *J* = 6.8 Hz, H-2``), 2.01 (1H, m, H-3``), 0.99 (3H, d, *J* = 6.6 Hz, H-4``), 0.99 (3H, d, *J* = 6.6 Hz, H-5``); ^{13}C NMR (150 MHz, $CDCl_3$): 137.7 (C-2), 123.6 (C-3), 127.4 (C-4), 138.7 (C-5), 70.3 (C-6), 46.0 (C-7), 136.8 (C-2`), 123.3 (C-3`), 131.7 (C-4`), 123.4 (C-5`), 72.8 (C-6`), 91.5 (C-7`), 4.7 (C-8`), 171.8 (C-1``), 43.3 (C-2``); 25.7 (C-3``), 22.4 (C-4``), 22.4 (C-5``); HRESIMS *m/z*, 389.0414 $[M+Na]^+$, calcd for $C_{18}H_{19}O_2NaS_2Cl$, 389.0413.	
Echinothiophene F (**63**)	[19]
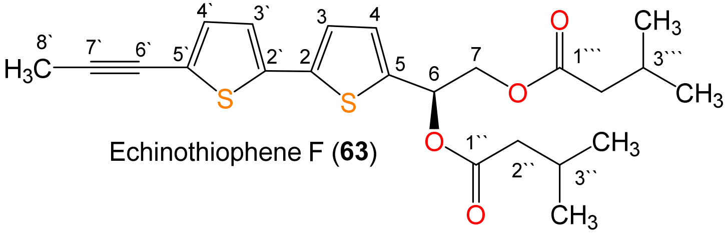 Echinothiophene F (**63**) Yellow oil; $[\alpha]_D^{25}$ +52.4 (*c* 0.11, MeOH); ECD (CH_3CN) λ_{max} ($\Delta\varepsilon$) 261 (-6.0) nm; ^{1}H NMR (600 MHz, $CDCl_3$): 7.02 (1H, d, *J* = 3.5 Hz, H-3), 7.01 (1H, d, *J* = 3.6 Hz, H-4), 6.28 (1H, dd, *J* = 7.5, 4.1 Hz, H-6), 4.46 (1H, dd, *J* = 11.6, 3.9 Hz, H-7A), 4.39 (1H, dd, *J* = 11.7, 7.6 Hz, H-7B), 6.99 (1H, d, *J* = 3.6 Hz, H-3`), 7.01 (1H, d, *J* = 3.6 Hz, H-4`), 2.11 (3H, s, H-8`), 2.25 (2H, d, *J* = 7.9 Hz, H-2``), 2.13 (1H, m, H-3``), 0.97 (3H, d, *J* = 6.6 Hz, H-4``), 0.97 (3H, d, *J* = 6.6 Hz, H-5``), 2.24 (2H, d, *J* = 7.9 Hz, H-2```), 2.15 (1H, m, H-3```), 0.97 (3H, d, *J* = 6.6 Hz, H-4```), 0.97 (3H, d, *J* = 6.6 Hz, H-5```); ^{13}C NMR (150 MHz, $CDCl_3$): 137.6 (C-2), 123.5 (C-3), 127.3 (C-4), 138.1 (C-5), 68.5 (C-6), 65.2 (C-7), 136.9 (C-2`), 123.5 (C-3`), 131.7 (C-4`), 126.3 (C-5`), 72.8 (C-6`), 91.4 (C-7`), 4.7 (C-8`), 171.9 (C-1``), 43.3 (C-2``); 25.7 (C-3``), 22.4 (C-4``), 22.4 (C-5``), 172.5 (C-1```), 43.2 (C-2```); 25.7 (C-3```), 22.4 (C-4```), 22.4 (C-5```); HRESIMS *m/z*, 455.1318 $[M+Na]^+$, calcd for $C_{23}H_{28}O_4NaS_2$, 455.1327.	
Ecliprostin A (**65**)	[18]
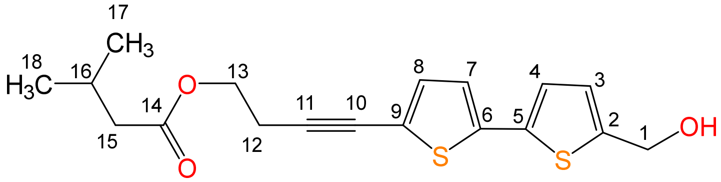 Ecliprostin A (**65**) Yellow powder; UV (MeOH) λ_{max} (log ε) 339 (4.33) nm; ^{1}H NMR (600 MHz, $CDCl_3$): 4.80 (2H, s, H-1), 6.90 (1H, d, *J* = 3.6 Hz, H-3), 7.01 (1H, d, *J* = 3.6 Hz, H-4), 6.97 (1H, d, *J* = 3.8 Hz, H-7), 7.02 (1H, d, *J* = 3.8 Hz, H-8), 2.78 (2H, t, *J* = 6.8 Hz, H-12), 4.25 (2H, t, *J* = 6.8 Hz, H-13), 2.23 (2H, d, *J*	

= 7.1 Hz, H-15), 2.13 (1H, m, H-16), 0.98 (3H, d, *J* = 6.6 Hz, H-17), 0.98 (3H, d, *J* = 6.6 Hz, H-18); ^{13}C NMR (150 MHz, $CDCl_3$): 60.4 (C-1), 143.7 (C-2), 126.4 (C-3), 123.9 (C-4), 137.2 (C-5), 138.2 (C-6), 123.5 (C-7), 132.6 (C-8), 122.5 (C-9), 75.3 (C-10), 91.1 (C-11), 20.6 (C-12), 62.0 (C-13), 173.2 (C-14), 43.6 (C-15); 26.0 (C-16), 22.6 (C-17), 22.6 (C-18); (+)-ESIMS: *m/z* 349.0 $[M+H]^+$, 371.0 $[M+Na]^+$; (+)-HRESIMS: *m/z* 349.0929 $[M+H]^+$ (calcd for $C_{18}H_{21}O_3S_2$, 349.0927).	
Ecliprostin B (**66**)	[18]
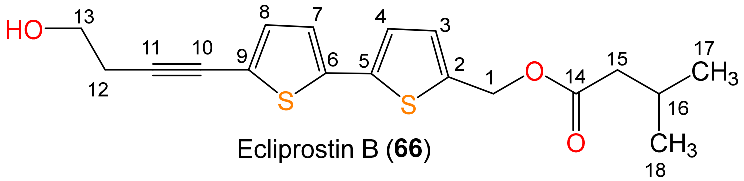 Ecliprostin B (**66**)	
Yellow powder; UV (MeOH) λ_{max} (log ε) 338 (4.26) nm; ^{1}H NMR (600 MHz, $CDCl_3$): 5.22 (2H, s, H-1), 6.98 (1H, d, *J* = 3.6 Hz, H-3), 7.01 (1H, d, *J* = 3.6 Hz, H-4), 6.99 (1H, d, *J* = 3.8 Hz, H-7), 7.05 (1H, d, *J* = 3.8 Hz, H-8), 2.73 (2H, t, *J* = 6.2 Hz, H-12), 3.83 (2H, t, *J* = 6.2 Hz, H-13), 2.23 (2H, d, *J* = 7.1 Hz, H-15), 2.12 (1H, m, H-16), 0.95 (3H, d, *J* = 6.7 Hz, H-17), 0.95 (3H, d, *J* = 6.7 Hz, H-18); ^{13}C NMR (150 MHz, $CDCl_3$): 60.5 (C-1), 137.9 (C-2), 129.1 (C-3), 123.7 (C-4), 138.3 (C-5), 137.8 (C-6), 123.7 (C-7), 132.7 (C-8), 122.6 (C-9), 75.7 (C-10), 92.0 (C-11), 24.4 (C-12), 61.2 (C-13), 173.1 (C-14), 43.5 (C-15); 25.9 (C-16), 22.6 (C-17), 22.6 (C-18); (+)-ESIMS: *m/z* 349.0 $[M+H]^+$, 371.0 $[M+Na]^+$; (+)-HRESIMS: *m/z* 371.0747 $[M+Na]^+$ (calcd for $C_{18}H_{20}O_3S_2Na$, 371.0746).	
Ecliprostin C (**67**)	[18]
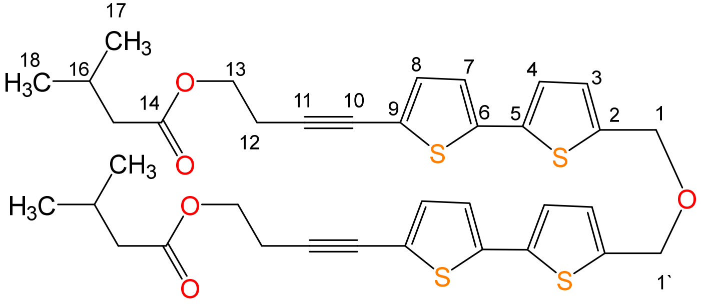 Ecliprostin C (**67**)	
Yellow powder; UV (MeOH) λ_{max} (log ε) 338 (4.15) nm; ^{1}H NMR (600 MHz, $CDCl_3$): 4.68 (4H, s, H-1, 1\`), 6.91 (2H, d, *J* = 3.5 Hz, H-3, 3\`), 7.02 (2H, d, *J* = 3.5 Hz, H-4, 4\`), 6.98 (2H, d, *J* = 3.8 Hz, H-7, 7\`), 7.03 (2H, d, *J* = 3.8 Hz, H-8, 8\`), 2.79 (4H, t, *J* = 6.8 Hz, H-12, 12\`), 4.25 (4H, t, *J* = 6.8 Hz, H-13, 13\`), 2.23 (4H, d, *J* = 7.2 Hz, H-15, 15\`), 2.13 (1H, m, H-16, 16\`), 0.98 (3H, d, *J* = 6.7 Hz, H-17, 17\`), 0.98 (3H, d, *J* = 6.7 Hz, H-18, 18\`); ^{13}C NMR	

(150 MHz, $CDCl_3$): 66.2 (C-1, 1), 140.3 (C-2, 2`), 127.8 (C-3, 3`), 123.8 (C-4, 4`), 137.2 (C-5, 5`), 138.2 (C-6, 6`), 123.5 (C-7, 7`), 132.6 (C-8, 8`), 122.5 (C-9, 9`), 75.3 (C-10, 10`), 91.1 (C-11, 11`), 20.6 (C-12, 12`), 62.0 (C-13, 13`), 173.1 (C-14, 14`), 43.6 (C-15, 15`); 26.0 (C-16, 16`), 22.7 (C-17, 17`), 22.7 (C-18, 18`);(+)-ESIMS: *m/z* 679.5 $[M+H]^+$, 701.5 $[M+Na]^+$; (+)-HR-ESIMS: *m/z* 679.1677 $[M+H]^+$ (calcd for $C_{36}H_{39}O_5S_4$, 679.1675).	
Echinbithiophenedimer A (**68**)	[17]
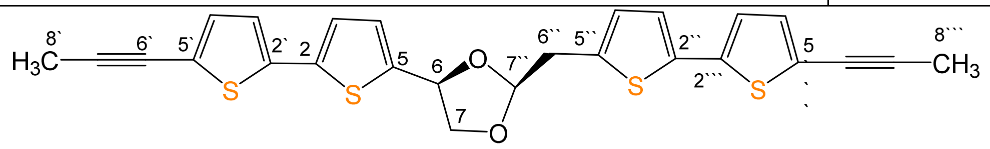<br>Echinbithiophenedimer A (**68**)<br>Yellow, amorphous powder; $[\alpha]_D^{25}$ +4.8 (c 0.1, MeOH); UV (CH3CN) λmax(log ε) 243 (0.53), 332 (1.75) nm; ECD (CH3CN) λmax(Δε) 222 (-7.9), 258 (+2.1) nm; IR (KBr)ν_{max}: 1633 and 1027 cm^{-1}; ^{1}H NMR (600 MHz, $CDCl_3$): 6.97 (1H, d, *J* = 3.5 Hz, H-3), 6.88 (1H, d, *J* = 3.5 Hz, H-4), 5.28 (1H, t, *J* = 6.5 Hz, H-6), 4.20 (1H, t, *J* = 7.9 Hz, H-7A), 3.91 (1H, dd, *J* = 7.9, 6.5 Hz, H-7B), 6.90 (1H, d, *J* = 3.8 Hz, H-3`), 6.98 (1H, overlapped, H-4`), 2.09 (3H, s, H-8`), 7.01 (1H, d, *J* = 3.7 Hz, H-3``), 6.84 (1H, d, *J* = 3.7 Hz, H-4``), 3.27 (2H, t, *J* = 4.3 Hz, H-6``), 5.27 (1H, t, *J* = 4.3 Hz, H-7``), 7.01 (1H, d, *J* = 3.3 Hz, H-3```), 6.98 (1H, overlapped, H-4```), 2.09 (3H, s, 8```); ^{13}C NMR (150 MHz, $CDCl_3$): 137.2 (C-2), 123.2 (C-3), 126.1 (C-4), 141.9 (C-5), 74.6 (C-6), 72.1 (C-7), 137.5 (C-2`), 123.4 (C-3`), 131.7 (C-4`), 122.6 (C-5`), 72.9 (C-6`), 91.3 (C-7`), 4.7 (C-8`), 136.3 (C-2``), 123.7 (C-3``); 127.9 (C-4``), 136.6 (C-5``), 35.2 (C-6``), 104.5 (C-7``), 137.8 (C-2```), 122.8 (C-3```), 131.7 (C-4```), 122.6 (C-5```), 73.0 (C-6```), 90.9 (C-7```), 4.7 (C-8```); HRESIMS *m/z*: 515.0236 ($[M+Na]^+$ calcd for $C_{26}H_{20}NaO_2S_4$, 515.0238).	
Echinbithiophenedimer B (**69**)	[17]
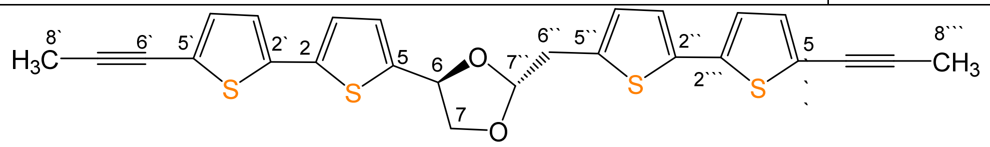<br>Echinbithiophenedimer B (**69**)<br>Yellow, amorphous powder; $[\alpha]_D^{25}$ +3.2 (*c* 0.1, MeOH); UV (CH_3CN) λ_{max} (log ε) 243 (0.49), 340 (1.47) nm; ECD (CH_3CN) λ_{max} (Δε) 231 (-15.4), 250 (+4.3) nm; IR (KBr)ν_{max}: 1653 and 1055 cm^{-1}; ^{1}H NMR (600 MHz, $CDCl_3$): 7.00 (1H, overlapped, H-3), 6.90 (1H, d, *J* = 3.4 Hz, H-4), 5.24 (1H, t, *J* = 6.6 Hz, H-6), 4.36 (1H, dd, *J* = 8.1, 6.6 Hz, H-7A), 3.89 (1H, t, *J* = 8.1 Hz, H-7B), 6.94 (1H, d, *J* = 3.6 Hz, H-3`), 6.98 (1H, overlapped, H-4`), 2.08 (3H, s, H-8`), 6.99 (1H, overlapped, H-3``), 6.82 (1H, d, *J* = 3.3 Hz, H-4``), 3.20 (2H, dd, *J* = 9.0, 4.0 Hz, H-6``), 5.45 (1H, t, *J* = 4.0 Hz, H-7``), 6.96 (1H, d, *J* = 3.6 Hz, H-3```), 6.98 (1H, overlapped, H-4```), 2.08 (3H, s, 8```); ^{13}C NMR (150 MHz, $CDCl_3$): 137.1 (C-2), 123.4 (C-3), 126.3 (C-4), 141.6 (C-5), 74.1 (C-6), 72.2 (C-7), 137.3 (C-2`), 123.6 (C-3`), 131.8 (C-4`), 122.6 (C-5`), 72.9 (C-6`), 91.3 (C-7`), 4.7 (C-8`), 136.4 (C-2``), 123.6 (C-3``); 127.7 (C-	

4``), 136.7 (C-5``), 35.3 (C-6``), 103.8 (C-7``), 137.8 (C-2```), 122.8 (C-3```), 131.8 (C-4```), 123.4 (C-5```), 73.0 (C-6```), 91.0 (C-7```), 4.7 (C-8```); HRESIMS *m/z*: 493.0420 ($[M+H]^+$ calcd for $C_{26}H_{21}O_2S_4$, 493.0419).	
Echinbithiophenedimer C (**70**)	[17]
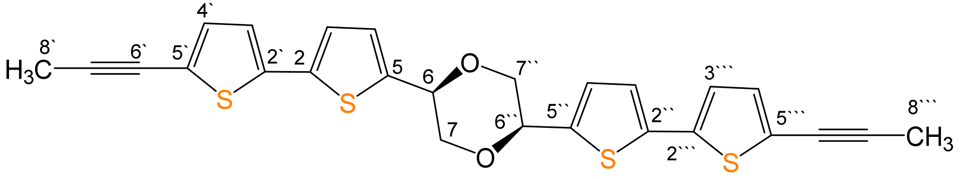 Echinbithiophenedimer C (**70**) Yellow, amorphous powder; $[\alpha]_D^{25}$ -2.3 (*c* 0.1, MeOH); UV (CH_3CN) λ_{max} (log ε) 245 (0.17), 338 (0.63) nm; ECD (CH_3CN) λ_{max} (Δε) 208 (-13.4), 255 (+3.5) nm; IR (KBr)ν_{max}: 1650 and 1026 cm^{-1}; ^{1}H NMR (600 MHz, $CDCl_3$): 7.07 (1H, d, *J* = 3.3 Hz, H-3), 7.05 (1H, d, *J* = 3.3 Hz, H-4), 4.96 (1H, t, *J* = 3.63 Hz, H-6), 4.16 (1H, dd, *J* = 11.9, 3.3 Hz, H-7A), 4.00 (1H, d, *J* = 11.9 Hz, H-7B), 7.01 (1H, d, *J* = 3.5 Hz, H-3`), 7.03 (1H, d, *J* = 3.5 Hz, H-4`), 2.11 (3H, s, H-8`), 7.07 (1H, d, *J* = 3.3 Hz, H-3``), 7.05 (1H, d, *J* = 3.3 Hz, H-4``), 4.96 (1H, t, *J* = 3.3 Hz, H-6``), 4.16 (1H, dd, *J* = 11.9, 3.3 Hz, H-7``A), 4.00 (1H, d, *J* = 11.9 Hz, 7``B), 7.01 (1H, d, *J* = 3.5 Hz, H-3```), 7.03 (1H, d, *J* = 3.5 Hz, H-4```), 2.11 (3H, s, 8```); ^{13}C NMR (150 MHz, $CDCl_3$): 137.2 (C-2), 123.5 (C-3), 127.1 (C-4), 139.8 (C-5), 74.4 (C-6), 67.1 (C-7), 137.4 (C-2`), 123.5 (C-3`), 131.8 (C-4`), 123.2 (C-5`), 72.9 (C-6`), 91.2 (C-7`), 4.7 (C-8`), 137.2 (C-2``), 123.5 (C-3``); 127.1 (C-4``), 139.8 (C-5``), 71.4 (C-6``), 67.1 (C-7``), 137.8 (C-2```), 123.2 (C-3```), 131.8 (C-4```), 123.2 (C-5```), 72.9 (C-6```), 91.2 (C-7```), 4.7 (C-8```); HRESIMS *m/z* 515.0240 ($[M+Na]^+$ calcd for $C_{26}H_{20}NaO_2S_4$, 515.0238).	
(*R*)-(5`-(3,4-dihydroxybut-1-yn-1-yl)-[2,2`-bithiophen]-5-yl)methyl 3-methylisovalerate (**71**)	[38]
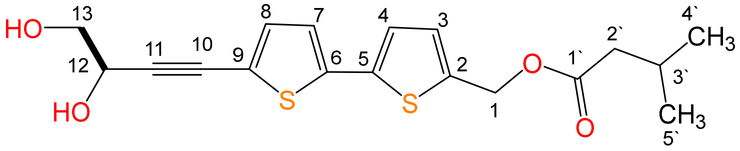 (*R*)-(5`-(3,4-dihydroxybut-1-yn-1-yl)-[2,2`-bithiophen]-5-yl)methyl 3-methylisovalerate (**71**) Yellow powder; UV (MeOH) λ_{max} (log ε) 338 (4.31) nm; ^{1}H NMR ($CDCl_3$, 600 MHz); 5.22 (2H, s, H-1), 6.98 (1H, d, *J* = 3.7 Hz, H-3), 7.03 (1H, d, *J* = 3.7 Hz, H-4), 7.00 (1H, d, *J* = 3.8 Hz, H-7), 7.11 (1H, d, *J* = 3.8 Hz, H-8), 4.71 (1H, dd, *J* = 6.6, 3.8 Hz, H-12), 3.84 (1H, dd, *J* = 11.3, 3.8 Hz, H-13A), 3.79 (1H, dd, *J* = 11.3, 6.6 Hz, H-13B), 2.23 (2H, d, *J* = 7.2 Hz, H-2`), 2.19-2.08 (1H, m, H-3`), 0.95 (3H, d, *J* = 7.2 Hz, H-4`), 0.95 (3H, d, *J* = 7.2 Hz, H-5`); ^{13}C NMR (150 MHz, $CDCl_3$): 60.5 (C-1), 138.2 (C-2), 129.1 (C-3), 124.1 (C-4), 138.0 (C-5), 139.2 (C-6), 123.7 (C-7), 133.8 (C-8), 120.9 (C-9),	

79.6 (C-10), 91.7 (C-11), 64.1 (C-12), 66.5 (C-13), 173.0 (C-1`), 43.5 (C-2`); 25.9 (C-3`), 22.6 (C-4`), 22.6 (C-5`); (+)-ESIMS: *m/z* 387.0 [M+Na]$^+$; (+)-HRESIMS: m/z 347.0776 [M–H_2O+H]$^+$ (calcd for $C_{18}H_{19}O_3S_2$, 347.0770).	
3`-Hydroxy-2,2`:5`,2``-terthiophene-3`-O-β-D-glucopyranoside (82)	[39]
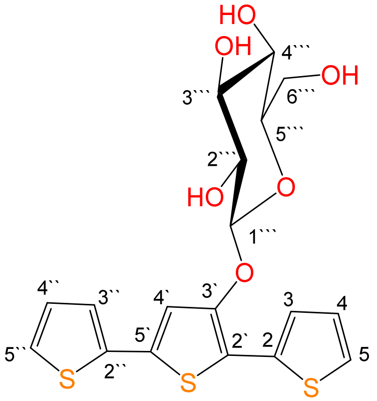<br>3`-Hydroxy-2,2`:5`,2``-terthiophene-3`-*O*-*β*-D-glucopyranoside (**82**)<br>Yellowish amorphous powder; $[\alpha]_D^{24}$ -69.08 (*c* 0.02, MeOH); UV λ_{max} (MeOH) nm (log *ε*): 208 (2.72), 260 (2.22), 340 (3.48), 345 (3.61), 351 (3.76), 356 (3.76), 364 (3.84), 379 (2.74); IR (ATR): 3360, 2923, 1733, 1650, 1566, 1508, 1426, 1380, 1253, 1074 cm^{-1}; ^{1}H NMR ($CDCl_3$, 500 MHz): 7.37 (1H, dd, *J* = 3.5, 1.0 Hz, H-3), 7.03 (1H, dd, *J* = 5.0, 3.5 Hz, H-4), 7.30 (1H, dd, *J* = 5.0, 1.0 Hz, H-5), 7.25 (1H, s, H-4`), 7.25 (1H, dd, *J* = 3.5, 1.0 Hz, H-3``), 7.04 (1H, dd, *J* = 5.0, 3.5 Hz, H-4``), 7.35 (1H, dd, *J* = 5.0, 1.0 Hz, H-5``), 5.04 (1H, d, *J* = 7.5Hz, H-1```), 3.56 (1H, dd, *J* = 9.0, 7.5 Hz, H-2```), 3.49 (1H, t, *J* = 9.0 Hz, H-3```), 3.41 (1H, t, *J* = 9.0 Hz, H-4```), 3.48 (1H, ddd, *J* = 9.5, 6.0, 2.5 Hz, H-5```), 3.91 (1H, dd, *J* = 12.0, 2.0 Hz, H-6```A), 3.71 (1H, dd, *J* = 12.0, 6.0 Hz, H-6```B); ^{13}C NMR ($CDCl_3$, 125 MHz): 134.0 (C-2), 123.5 (C-3), 126.7 (C-4), 123.8 (C-5), 115.9 (C-2`), 149.7 (C-3`), 115.6 (C-4`), 132.4 (C-5`), 136.9 (C-2``), 123.3 (C-3``), 127.7 (C-4``), 124.6 (C-5``), 102.3 (C-1```), 73.7 (C-2```), 76.9 (C-3```), 70.0 (C-4```),77.1 (C-5```), 61.1 (C-6```); HRDARTMS (positive mode) *m/z*: 427.0347 [M+H]$^+$ (Calcd for $C_{18}H_{19}O_6S_3$: 427.0344).	
Thiotagetin A (**83**)	[48]
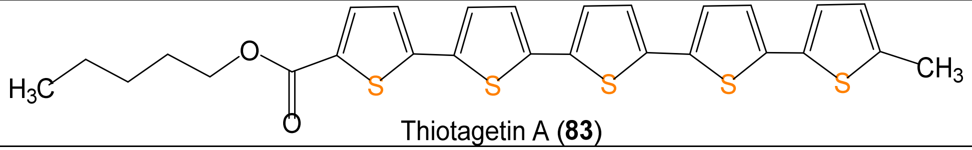<br>Thiotagetin A (**83**)	

Yellow amorphous powder; UV (MeOH) λ_{max} (log ε): 335 (3.45), 389 (4.11) nm; IR (KBr) ν_{max}: 2896, 1729, 839, 801, 717 cm^{-1}; ^1H NMR (500 MHz, $CDCl_3$): 2.42 (3H, s, H-1), 6.64 (1H, brs, H-3), 6.93 (1H, d, J = 3.5 Hz, H-4), 6.96 (1H, d, J = 3.5 Hz, H-7), 7.04 (1H, brs, H-8), 6.97 (1H, d, J = 3.5 Hz, H-11), 7.12 (1H, d, J = 3.5 Hz, H-12), 6.98 (1H, d, J = 3.5 Hz, H-15), 7.14 (1H, d, J = 3.5 Hz, H-16), 7.02 (1H, d, J = 3.5 Hz, H-19), 7.17 (1H, brs, H-20), 4.22 (2H, t, J = 6.8 Hz, H-23), 1.67 (2H, m, H-24), 1.43 (2H, m, H-25), 1.24 (2H, m, H-26), 0.98 (3H, t, J = 6.7 Hz, H-27); ^{13}C NMR (125 MHz, CDCl3): 15.7 (C-1), 137.0 (C-2), 124.3 (C-3), 125.9 (C-4), 133.7 (C-5), 134.2 (C-6), 121.2 (C-7), 122.3 (C-8), 128.9 (C-9), 134.7 (C-10), 121.5 (C-11), 122.3 (C-12), 126.8 (C-13), 135.1 (C-14), 125.8 (C-15), 121.6 (C-16), 132.5 (C-17), 135.5 (C-18), 122.5 (C-19), 121.7 (C-20), 137.0 (C-21), 165.8 (C-22), 66.1 (C-23), 28.4 (C-24), 26.9 (C-25), 21.0 (C-26), 13.4 (C-27); HRESIMS m/z 541.0459 $[M+H]^+$ (calcd for $C_{27}H_{25}O_2S_5$, 541.0452).

Sibiricumthionol (**84**)	[49]

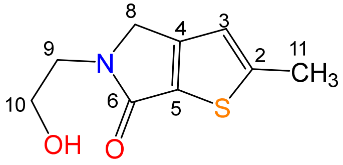

Sibiricumthionol (**84**)

White amorphous powder; UV λ_{max} (MeOH) (log ε): 203 (4.17), 268 (3.91) nm; IR (KBr) ν_{max}: 3371, 2939, 1651, 1512, 1454, 1266 cm^{-1}; ^1H NMR (CD_3OD, 600 MHz): 6.81 (1H, s, H-3), 4.41 (2H, brs, H-8), 3.58 (2H, t, J = 5.5 Hz, H-9), 3.70 (2H, t, J = 5.5 Hz, H-10), 2.53 (3H, brs, 11-CH_3); ^{13}C NMR (CD_3OD, 150 MHz): 152.8 (C-2), 121.3 (C-3), 154.8 (C-4), 133.5 (C-5), 167.5 (C-6), 51.9 (C-8), 46.9 (C-9), 61.7 (C-10), 16.4 (C-11); HRESIMS: m/z 198.0581 $[M+H]^+$ (calcd for $C_9H_{12}NO_2S$, 198.0583).

Rupestriene A (**86**)	[36]

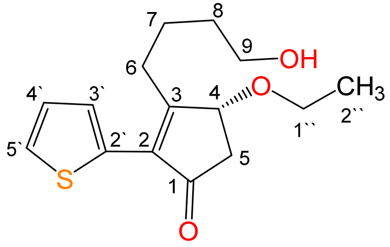

Rupestriene A (**86**)

Brown oil; $[\alpha]_D^{21}$ + 16 (c 0.1, MeOH); IR (KBr) ν_{max}: 3399, 2934, 1703, 1133, 837, 722 cm^{-1}; ^1H NMR (CD_3OD, 500 MHz): 4.70 (1H, dd, J = 6.0, 2.0 Hz, H-4), 2.82 (1H, dd, J = 18.2, 6.0 Hz, H-5A), 2.39 (1H, dd, J = 18.2, 2.0 Hz, H-5B), 2.86-2.84 (1H, m, H-6A), 2.78-2.76 (1H, m, H-6B), 1.75-1.73 (2H, m, H-7), 1.65-1.63 (2H, m, H-8), 3.58 (2H, t, J = 6.4 Hz, H-9), 7.48 (1H, d, J = 3.7 Hz, H-3`), 7.11 (1H, dd, $J$ = 5.2, 3.7 Hz, H-4`), 7.50 (1H, d, J = 5.6

Hz, H-5\`), 3.65 (2H, q, *J* = 7.0 Hz, H-1\`\`), 1.25 (3H, t, *J* = 7.0 Hz, H-2\`\`); $^{13}$C NMR ($CD_3OD$, 500 MHz): 205.1 (C-1), 135.0 (C-2), 172.5 (C-3), 77.7 (C-4), 42.7 (C-5), 29.8 (C-6), 24.7 (C-7), 33.8 (C-8), 62.5 (C-9), 129.1 (C-2\`), 132.7 (C-3\`), 127.9 (C-4\`), 127.9 (C-5\`), 66.7 (C-11\`), 15.7 (C-2\`\`); positive HRESIMS: *m*/*z* 303.1030 [M+Na]$^+$ (calcd for $C_{15}H_{20}SO_3$, 303.1025).	
7-[1-(Thiophene-5-yl)-1-formamido]-3-propylenyl-3-cephem-4-carboxylic acid (CAx1) (**87**)	[50]
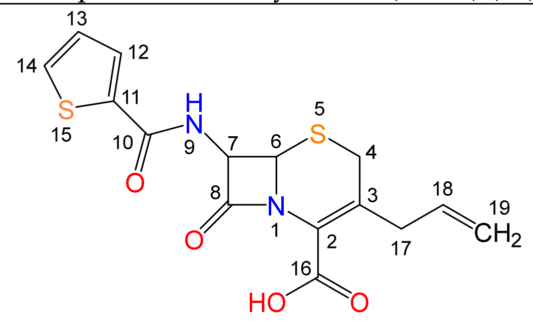 7-[1-(Thiophene-5-yl)-1-formamido]-3-propylenyl-3-cephem-4-carboxylic acid (CAx1) (**87**)	
White amorphous powder; UV (MeOH) λ_{max}: 369, 348, 258 nm; IR (KBr)ν_{max}: 3502, 3443, 3041, 2969, 1770, 1686, 1605, 1573, 1457, 1078, 847, 720, 707, 696 cm^{-1}; ^{1}H NMR (400 MHz, DMSO-d_6): 2.49 (2H, s, –CH_2–), 3.41 (2H, s, –S–CH_2–), 4.80 (2H, s, =CH_2), 4.89 (1H, s, Ar–H), 5.29 (1H, s, Ar–H), 5.40 (1H, s, =CH–), 7.33 (1H, d, *J* = 13.2 Hz, =CH–), 7.44 (1H, d, *J* = 7.0 Hz, =CH–S–), 7.55 (1H, d, *J* = 7.08 Hz, =CH–); ^{13}C NMR (100 MHz, DMSO-d_6): (C-2), 128.34 (C-3), 30.82 (C-4), 57.57 (C-6), 58.43 (C-7), 169.49 (C-8), 161.87 (C-10), 144.35 (C-11), 128.06 (C-12), 127.33 (C-13), 128.34 (C-14), 169.64 (C-16), 26.87 (C-17), 136.38 (C-18) and 109.65 (C-19); HREIMS [M]$^+$, *m*/*z* 350.1180, $C_{15}H_{14}N_2O_4S_2$.	
2,5-Bis(5-tert-butyl-2-benzoxazolyl)thiophene (**88**)	[51]
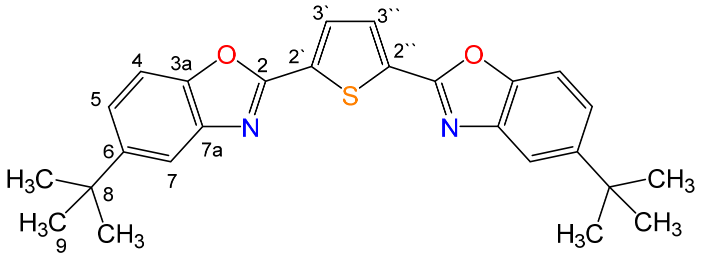 2,5-Bis(5-tert-butyl-2-benzoxazolyl)thiophene (**88**)	

White solid; MP. 202-203 °C; FT-IR (KBr)ν_{max}: 3442, 2959, 2867, 1635, 1581, 1504, 1478, 1362, 1348, 1266, 1195, 1027, 935, 810, 715 cm^{-1}; UV (MeOH) λ_{max} (log ε): 209 (1.08), 247 (0.38), 264 (0.37), 370 (1.77), 3.73 (1.78) nm; ^{1}H NMR (500 MHz, DMSO-d_6): 1.37 (9H, s, 3 x CH_3), 7.54 (1H, dd, *J* = 2.0, 8.0 Hz, H-5), 7.72 (1H, d, *J* = 8.0 Hz, H-4), 7.81 (1H, d, *J* = 2.0 Hz, H-7), 8.06 (1H, s, H-3`); $^{13}C$ NMR (125 MHz, DMSO-$d_6$): 31.5 (3 x CH3), 34.8 (C-8), 110.3 (C-4), 116.3 (C-7), 123.9 (C-5), 131.3 (C-3`), 132.4 (C-2`), 141.3 (C-3a), 148.3 (C-6), 148.6 (C-7a), 157.5 (C-2); HR-ESI-MS: *m/z* 431.1785 $[M+H]^+$ (calcd. for $C_{26}H_{27}N_2O_2S$, 431.1793).	
Thiocarboxylic A (**89**)	[16]
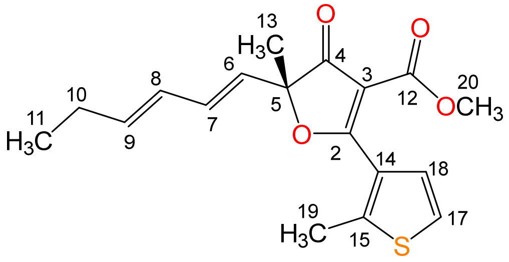 Thiocarboxylic A (**89**) Yellow oil; $[\alpha]_D^{20}$ -43 (*c* 0.2, $CHCl_3$); UV (CH_3OH) λ_{max} (log ε) 235 (3.20), 325 (2.23) nm; ECD (*c* 0.3 mg/mL, CH_3OH) λ_{max} ($\Delta\varepsilon$) 246 (5.64), 268 (1.03), 280 (1.53), 332 (-2.59) nm; IR (KBr)ν_{max} 1709, 1588, 1444, 1403, 1064 cm^{-1}; ^{1}H NMR ($CDCl_3$, 400 MHz); 5.62 (1H, d, *J* = 15.5 Hz, H-6), 6.34 (1H, dd, *J* = 15.5, 10.4 Hz, H-7), 6.00 (1H, dd, *J* = 15.3, 10.3 Hz, H-8), 5.83 (1H, dt, *J* = 15.2, 7.4 Hz, H-9), 2.09 (2H, m, H-10), 0.99 (3H, t, *J* = 7.4 Hz, H-11), 1.62 (3H, m, H-13), 7.12 (1H, d, *J* = 5.4 Hz, H-17), 7.36 (1H, d, *J* = 5.4 Hz, H-18), 2.64 (3H, s, H-19), 3.82 (3H, s, H-20); ^{13}C NMR ($CDCl_3$, 100 MHz): 184.4 (C-2), 111.2 (C-3), 198.5 (C-4), 90.8 (C-5), 125.9 (C-6), 132.2 (C-7), 127.9 (C-8), 139.7 (C-9), 25.8 (C-10), 13.4 (C-11), 163.2 (C-12), 23.0 (C-13), 128.6 (C-14), 141.9 (C-15), 122.2 (C-17), 129.5 (C-18), 15.8 (C-19), 52.0 (C-20); (+)-HRESIMS $[M+Na]^+$ *m/z* 355.0981 (calcd for $C_{18}H_{20}O_4SNa$, 355.0980).	
Thiocarboxylic B (**90**)	[16]

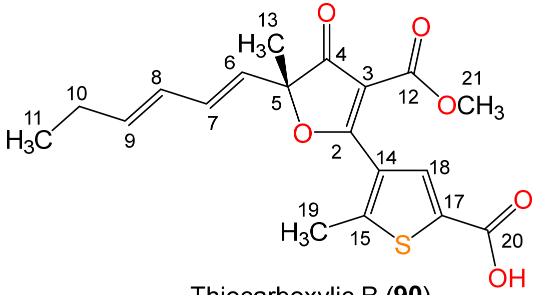<br>Thiocarboxylic B (**90**)<br>Yellow oil; $[\alpha]_D^{20}$ - 46 (*c* 0.1, $CHCl_3$); UV (CH3OH) λ_{max} (log ε) 233 (3.20), 315 (2.24) nm; ECD (*c* 0.3 mg/mL, CH3OH) λ_{max} (Δε) 242 (3.70), 259 (1.00), 277 (2.55), 317 (-1.98) nm; IR (film) ν_{max} 1712, 1587, 1441, 1390, 1128 cm^{-1}; ^{1}H NMR ($CDCl_3$, 400 MHz); 5.64 (1H, d, *J* = 15.5 Hz, H-6), 6.38 (1H, dd, *J* = 15.5, 10.4 Hz, H-7), 5.97 (1H, dd, *J* = 15.1, 10.4 Hz, H-8), 5.78 (1H, dt, *J* = 15.2, 6.5 Hz, H-9), 2.08 (2H, m, H-10), 0.97 (3H, t, *J* = 7.4 Hz, H-11), 1.61 (3H, m, H-13), 7.70 (1H, s, H-18), 2.40 (3H, s, H-19), 3.62 (3H, s, H-21), 3.82 (3H, s, H-20); ^{13}C NMR ($CDCl_3$, 100 MHz): 190.4 (C-2), 108.5 (C-3), 198.8 (C-4), 91.5 (C-5), 125.8 (C-6), 132.1 (C-7), 127.5 (C-8), 139.4 (C-9), 25.8 (C-10), 13.4 (C-11), 162.6 (C-12), 22.5 (C-13), 128.2 (C-14), 141.5 (C-15), 128.0 (C-17), 128.0 (C-18), 14.3 (C-19), 167.9 (C-20), 51.4 (C-21); HRESIMS $[M+Na]^+$ *m/z* 399.0875 (calcd for $C_{19}H_{20}O_6SNa$, 399.0878).	
Thiocarboxylic C1 (**91**)	[16]
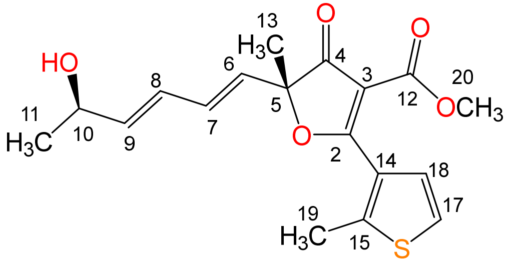<br>Thiocarboxylic C1 (**91**)<br>Yellow oil; $[\alpha]_D^{20}$ -44 (*c* 0.1, CHCl3); UV (CH_3OH) λ_{max} (log ε) 235 (3.19), 325 (2.14) nm; ECD (*c* 0.3 mg/mL, CH_3OH) λ_{max} (Δε) 246 (5.92), 268 (1.26), 280 (1.61), 332 (-2.71) nm; IR (film) ν_{max} 3462, 1710, 1588, 1444, 1402, 1063 cm^{-1}; 5.75 (1H, d, *J* = 15.4 Hz, H-6), 6.36 (1H, dd, *J* = 15.4, 10.5 Hz, H-7), 6.18 (1H, dd, *J* = 15.3, 10.5 Hz, H-8), 5.83 (1H, dd, *J* = 15.3, 6.1 Hz, H-9), 4.36 (2H, m, H-10), 1.28 (3H, t, *J* = 6.4 Hz, H-11), 1.63 (3H, m, H-13), 7.13 (1H, d, *J* = 5.5 Hz, H-17), 7.36 (1H, d, *J* = 5.4 Hz, H-18), 2.64 (3H, s, H-19), 3.82 (3H, s, H-20); ^{13}C NMR ($CDCl_3$, 100 MHz): 184.4 (C-2), 106.2 (C-3),	

198.5 (C-4), 90.5 (C-5), 128.7 (C-6), 131.0 (C-7), 127.8 (C-8), 140.1 (C-9), 68.3 (C-10), 23.4 (C-11), 163.1 (C-12), 23.0 (C-13), 126.6 (C-14), 147.9 (C-15), 122.3 (C-17), 129.4 (C-18), 15.9 (C-19), 52.0 (C-20); (+)-HRESIMS [M + Na]+ *m/z* 371.0926 (calcd for $C_{18}H_{20}O_5SNa$, 371.0929).	
Thiocarboxylic C2 (**92**)	[16]
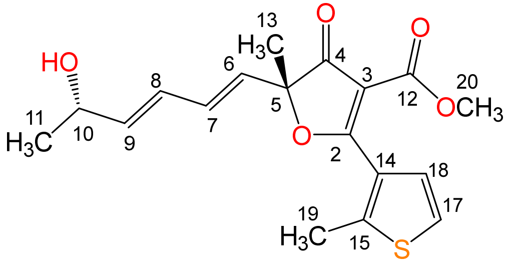 Thiocarboxylic C2 (**92**) Yellow oil; $[\alpha]_D^{20}$ -40 (c 0.1, $CHCl_3$); UV (CH_3OH) λ_{max} (log ε) 235 (3.19), 325 (2.14) nm; ECD (*c* 0.3 mg/mL, CH_3OH) λ_{max} (Δε) 246 (6.60), 268 (1.38), 280 (1.91), 333 (-2.94) nm; IR (film) ν_{max} 3462, 1710, 1588, 1444, 1402, 1063 cm^{-1}; ^1H NMR ($CDCl_3$, 400 MHz); 5.75 (1H, d, *J* = 15.4 Hz, H-6), 6.36 (1H, dd, *J* = 15.4, 10.5 Hz, H-7), 6.18 (1H, dd, *J* = 15.3, 10.5 Hz, H-8), 5.83 (1H, dd, *J* = 15.3, 6.1 Hz, H-9), 4.36 (2H, m, H-10), 1.28 (3H, t, *J* = 6.4 Hz, H-11), 1.63 (3H, m, H-13), 7.13 (1H, d, *J* = 5.5 Hz, H-17), 7.36 (1H, d, *J* = 5.4 Hz, H-18), 2.64 (3H, s, H-19), 3.82 (3H, s, H-20); ^{13}C NMR ($CDCl_3$, 100 MHz): 184.4 (C-2), 106.2 (C-3), 198.5 (C-4), 90.5 (C-5), 128.7 (C-6), 131.0 (C-7), 127.8 (C-8), 140.1 (C-9), 68.3 (C-10), 23.4 (C-11), 163.1 (C-12), 23.0 (C-13), 126.6 (C-14), 147.9 (C-15), 122.3 (C-17), 129.4 (C-18), 15.9 (C-19), 52.0 (C-20); (+)-HRESIMS $[M+Na]^+$ *m/z* 371.0926 (calcd for $C_{18}H_{20}O_5SNa$, 371.0929).	
Thiocarboxylic D1 (**93**)	[16]
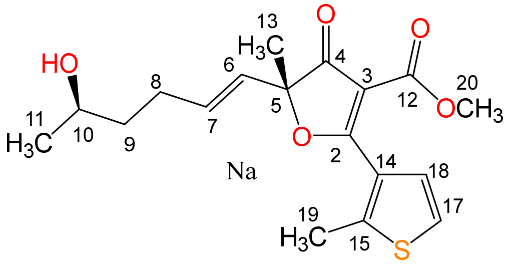 Thiocarboxylic D1 (**93**) Yellow oil; $[\alpha]_D^{20}$ -46 (*c* 0.1, $CHCl_3$); UV (CH_3OH) λ_{max} (log ε) 235 (3.19), 325 (2.14) nm; ECD (*c* 0.3 mg/mL, CH3OH) λ_{max} (Δε) 220 (2.60), 240 (-0.29), 282 (2.77), 336 (-5.23) nm; IR (film) ν_{max} 3410, 1712, 1579, 1443, 1385, 1104 cm^{-1}; ^1H NMR ($CDCl_3$, 400 MHz); ^1H NMR ($CDCl_3$, 400 MHz);	

5.58 (1H, dt, *J* = 15.7, 1.5 Hz, H-6), 5.89 (1H, dt, *J* = 15.6, 6.8 Hz, H-7), 2.16 (2H, m, H-8), 1.51 (2H, m, H-9), 3.79 (1H, m, H-10), 1.19 (3H, t, *J* = 6.2 Hz, H-11), 1.60 (3H, m, H-13), 7.12 (1H, d, *J* = 5.5 Hz, H-17), 7.35 (1H, d, *J* = 5.5 Hz, H-18), 2.63 (3H, s, H-19), 3.82 (3H, s, H-20); ^{13}C NMR ($CDCl_3$, 100 MHz): 184.5 (C-2), 106.1 (C-3), 199.0 (C-4), 90.8 (C-5), 126.4 (C-6), 133.2 (C-7), 38.2 (C-8), 28.8 (C-9), 67.6 (C-10), 23.7 (C-11), 163.2 (C-12), 22.8 (C-13), 126.7 (C-14), 147.7 (C-15), 122.3 (C-17), 129.4 (C-18), 15.8 (C-19), 52.0 (C-20); (+)-HRESIMS $[M+H]^+$ *m*/*z* 373.1083 (calcd for $C_{18}H_{22}O_5SNa$, 373.1086).	
Thiocarboxylic D2 (**94**)	[16]
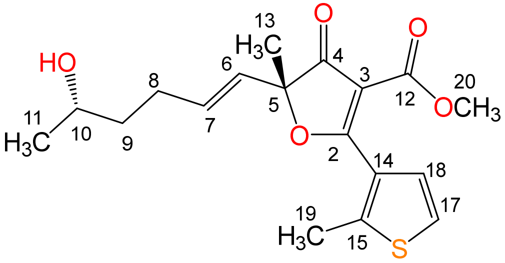 Thiocarboxylic D2 (**94**) yellow oil; $[\alpha]_D^{20}$ -41 (*c* 0.1, $CHCl_3$); UV (CH_3OH) λ_{max} (log ε) 235 (3.19), 325 (2.14) nm; ECD (*c* 0.3 mg/mL, CH_3OH) λ_{max} (Δε) 233 (-0.49), 278 (1.14), 338 (-4.38) nm; IR (film) ν_{max} 3410, 1712, 1579, 1443, 1385, 1104 cm^{-1}; ^{1}H NMR ($CDCl_3$, 400 MHz); 5.58 (1H, dt, *J* = 15.7, 1.5 Hz, H-6), 5.89 (1H, dt, *J* = 15.6, 6.8 Hz, H-7), 2.15 (2H, m, H-8), 1.52 (2H, m, H-9), 3.78 (1H, m, H-10), 1.18 (3H, t, *J* = 6.2 Hz, H-11), 1.59 (3H, m, H-13), 7.12 (1H, d, *J* = 5.4 Hz, H-17), 7.35 (1H, d, *J* = 5.4 Hz, H-18), 2.63 (3H, s, H-19), 3.82 (3H, s, H-20); ^{13}C NMR ($CDCl_3$, 100 MHz): 184.5 (C-2), 106.1 (C-3), 199.0 (C-4), 90.8 (C-5), 126.4 (C-6), 133.2 (C-7), 38.2 (C-8), 28.8 (C-9), 67.6 (C-10), 23.7 (C-11), 163.2 (C-12), 22.8 (C-13), 126.7 (C-14), 147.7 (C-15), 122.3 (C-17), 129.4 (C-18), 15.8 (C-19), 52.0 (C-20); (+)-HRESIMS $[M+H]^+$ *m*/*z* 373.1083 (calcd for $C_{18}H_{22}O_5SNa$, 373.1086).	
Rupestriene D (**95**)	[15]
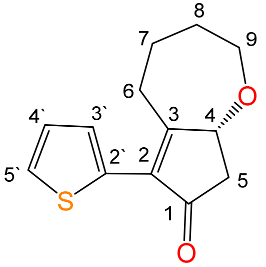 Rupestriene D (**95**)	

Brown oils; $[\alpha]_D^{25}$ +6.0 (*c* 0.30, MeOH); UV λ_{max} (MeOH) 254 nm; IR (KBr)ν_{max} 3090, 2936, 2855, 1704, 1101 cm^{-1}; ^{1}H NMR (CDCl$_3$, 400 MHz); 4.70 (1H, m, H-4), 2.94 (1H, m, H-5A), 2.46 (1H, dd, *J* = 18.4, 2.8 Hz, H-5B), 2.85 (2H, m, H-6), 1.95 (2H, m, H-7), 1.95 (1H, m, H-8A), 1.79 (1H, m, H-8B), 3.90 (1H, m, H-9A), 3.62 (1H, ddd, *J* = 12.2, 10.1, 2.1 Hz, H-9B), 7.61 (1H, d, *J* = 3.7 Hz, H-3\`), 7.12 (1H, dd, *J* = 5.1, 3.7 Hz, H-4\`), 7.41 (1H, d, *J* = 5.1 Hz, H-5\`); $^{13}$C NMR (CDCl$_3$, 100 MHz): 202.2 (C-1), 133.0 (C-2), 170.6 (C-3), 79.5 (C-4), 42.8 (C-5), 32.2 (C-6), 25.4 (C-7), 32.7 (C-8), 71.6 (C-9), 132.0 (C-2\`), 127.9 (C-3\`), 126.9 (C-4\`), 126.9 (C-5\`); ESIMS m/z 235 [M H]$^+$; HRESIMS *m*/*z* 235.0787 [M+H]$^+$ (calcd for $C_{13}H_{15}O_2S$, *m*/*z* 235.0787).	
Rupestriene E (**96**)	[15]
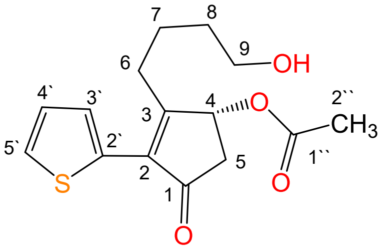<br>Rupestriene E (**96**)<br>Brown oils; $[\alpha]_D^{25}$ +9.0 (*c* 0.30, MeOH); UV λ_{max} (MeOH) 254 nm; IR (KBr) ν_{max} 3452, 2942, 1732, 1227, 1036 cm^{-1}; ^{1}H NMR (CD$_3$OD, 400 MHz); 4.91 (1H, dd, *J* = 6.4, 2.0 Hz, H-4), 2.40 (1H, dd, *J* = 18.4, 2.0 Hz, H-5A), 2.35 (1H, dd, *J* = 18.4, 2.0 Hz, H-5B), 2.93 (1H, m, H-6A), 2.88 (1H, m, H-6B), 1.77 (2H, m, H-7), 1.77 (2H, m, H-8), 4.12 (2H, t, *J* = 6.0 Hz, H-9), 7.46 (1H, d, *J* = 3.6 Hz, H-3\`), 7.14 (1H, dd, *J* = 5.2, 3.6 Hz, H-4\`), 7.53 (1H, d, *J* = 5.2 Hz, H-5\`); $^{13}$C NMR (CD$_3$OD, 100 MHz): 205.7 (C-1), 134.8 (C-2), 173.4 (C-3), 70.5 (C-4), 45.7 (C-5), 29.7 (C-6), 25.1 (C-7), 30.3 (C-8), 65.6 (C-9), 133.2 (C-2\`), 129.3 (C-3\`), 128.3 (C-4\`), 128.1 (C-5\`), 174.5 (C-1\`\`), 21.3 (C-2\`\`); ESI-MS m/z 293 [M-H]$^-$; HR-ESI-MS m/z 293.0856 [M-H]$^-$ (calcd for $C_{15}H_{17}O_4S$, *m*/*z* 293.0853).	
